# Supplementary material for: Family attitudes, actions, decisions and experiences following implementation of deemed consent and the Human Transplantation (Wales) Act 2013: mixed-method study protocol
Source: BMJ Open. 2017 Oct 12;7(10):e017287. doi: 10.1136/bmjopen-2017-017287 (PMC5652527; doi:10.1136/bmjopen-2017-017287)
Supplement: Supplementary data [file bmjopen-2017-017287supp001.pdf]

Appendix 1. Summary of datasets and evidence contributing to the mixed-method evaluation

	Dataset/Evidence	Content
		**Anonymous data shared by NHSBT under data sharing agreement**
1	**Anonymised NHSBT Log.**	The log records details of all approach conversations that Specialist Nurses in Organ Donation had with a potential donor family for whom the Act applied over the data collection period (18 months) 01/12/15 -31/05/17. It was created specially to capture specific details of the consent conversation after the law changed in Wales. The log includes:<br>1. A record of whether the deceased died via a Donation by Brain Stem Death (DBD) or Donation by Circulatory Death (DCD).<br>2. The deceased person's registered status on the Organ Donor Register (ODR) – Registered In/out or no registration found.<br>3. Type of Consent – Organ Donation Register In/Out, Expressed Consent In/Out, Deemed Consent and family consent (for those who did not fulfil the criteria to have their consent deemed).<br>4. Patients expressed decision – donate all organs, does not want to donate, no decision made.<br>5. Who the SNOD had the conversation with.<br>6. Did the family accept the known decision of the deceased person.<br>7. Reason why family objected to the known decision or the deemed consent.<br>8. If organ donation proceeded – the comments in number 11 will document if the donation stood down due to a medical reason or via the influences of the family, see number 11.<br>9. Who undertook the donation conversation.<br>10. Did family know about the Welsh Legislation.<br>11. Comments (to include evidence/information provided by families who are unable to support known decision/deemed consent).<br>12. Feedback/additional training requirements to staff – did this particular case highlight any areas for further professional development training.<br><br>Descriptive statistics report totals for categorical data.
2	**Summary statistics from NHSBT for the 18 month data collection window for Wales only.**	NHSBT summary of descriptive statistics specially prepared for the research team to cover the data collection window (01/12/15-31/05/17). Includes summary data on: organ donation registration; consent and deemed consent numbers; age range; ethnicity and reasons why donation not proceeded.
		**Routinely collected and publicly available NHSBT data**
3	**Publicly Available NHSBT Audit Data (Wales).**	NHSBT annual audit data runs from 01st April – 31st March and is available online for current and previous years. Relevant data mapped onto this study includes: Organ Donation Registration data; Number of deceased donors; Consent rates and deemed consent rates.

	**Continuous annual audit.**	
4	**Publicly Available NHSBT Audit Data (UK). Continuous annual audit.**	As above data also includes UK figures for:<br>Reasons why consent not given/decision not supported<br>Age, Ethnicity, gender of donors.
5	**Organ Donation Register UK.**	A new UK organ donor register was introduced in July 2015. People have the opportunity to Opt in, Opt Out and appoint a representative. Registration behaviour figures and trends will be used to contextualise study findings.
		**Publicly Available Welsh Government Commissioned Research**
6	**Focus groups with SNODS**	Welsh Government commissioned three sequential focus groups with SNODS, before, immediately after implementation and a year after the changes were introduced. Final focus group findings shared ahead of publication.
7	**Ombudsman Surveys**	Welsh Government commissioned 12 sequential public opinion surveys undertaken with the Welsh public in the years before and after the law changed. Wave 10 of the survey focused on monitoring awareness levels and understanding of the change in law and included additional questions to measure awareness and recall of publicity campaign material. Wave 11 and 12 focused on awareness and understanding as well as attitudes and behaviour.
8	**Literature reviews**	Systematic reviews of the literature on family attitudes to organ donation and reasons why donation is declined.
		**Additional data collection by the research team**
9	**Anonymous Family, Questionnaire FORM C.**	Families are sent a questionnaire capturing basic information on their understanding of the changes and their feelings about supporting their loved one's donation decision. Appendix 3. FORM C: Questionnaire completed by family members/close friends.
10	**Interviews with families**	Depth Interviews with families of a minimum of 50 cases to explore their views on organ donation, the Act, the media campaign and their donation experience. **Appendix 4**. Family interview schedule.
11	**Anonymous SNOD Questionnaire Form B.**	SNODS complete a questionnaire after each approach conversation to document information on the family's understanding of their role, their attitudes and behaviours and the outcome of the process. Appendix 1. FORM B: Completed by Specialist Nurse in Organ donation**.**
12<br>12.1<br>12.2	**Focus Groups with SNODS.**<br>**Interviews with Specialist Requesters.**<br>**Interviews with**	Focus groups with key SNODS, managers and specialist requesters in the North West team and South Wales team to explore SNODS experiences of implementing the act in practice. Minimum 23 participants.

	**team and regional managers**	
13	**Field Notes from interviews**	Researchers and transcribers document their thoughts and views from interview.
14	**Interim feedback from Patient and Public representatives, (PPI's), SNOD's, Managers, NHSBT, NHS, Clinical Leads Organ donation, and other key stakeholders.**	A two day interim findings conference was held in Birmingham on the 9th and 10th November 2016. The purpose was to present interim findings to a key group of 50 NHSBT staff, NHS staff, Welsh Government representatives and PPIs. Feedback was collected on 10 presentations reviewing the various datasets thus far.
15	**Research team perspectives**	Weekly team meetings and monthly data analysis meetings are recorded to capture the ongoing analysis and interpretation of data and to put findings into wider context and highlight issues needing further attention.
		**Additional contextual data produced by the research team to situate the evaluation findings**
16	**Update of the literature**	Update of the systematic reviews in 8.
17	**Discourse Analysis of the press & media**	The discourse analysis will include the public media campaign, press articles and news stories promoting the changes.
