# Supplementary material for: Family attitudes, actions, decisions and experiences following implementation of deemed consent and the Human Transplantation (Wales) Act 2013: mixed-method study protocol
Source: BMJ Open. 2017 Oct 12;7(10):e017287. doi: 10.1136/bmjopen-2017-017287 (PMC5652527; doi:10.1136/bmjopen-2017-017287)
Supplement: Supplementary data [file bmjopen-2017-017287supp002.pdf]

# FORM B Organ Donation Study: Questionnaire for SNOD to document outcome of all approach conversations

**Record of conversation with (tick all that apply): spouse or partner** ☐ **parent or child** ☐ **brother or sister** ☐ **grandparent or grandchild** ☐ **niece or nephew** ☐ **stepfather or stepmother** ☐ **half-brother or half-sister** ☐ **friend of long standing** ☐ **appointed representative** ☐ (referred to as 'family' hereafter)

**Age range of deceased person:** ☐ **0-18** ☐ **19-35** ☐ **36-50** ☐ **51-70** ☐ **>71**

**What region in Wales did family members/friends come from (tick all that apply):** ☐ Isle of Anglesey ☐ Conwy ☐ Gwynedd ☐ Denbighshire ☐ Flintshire ☐ Wrexham ☐ Powys ☐ Ceredigion ☐ Pembrokeshire ☐ Carmarthenshire ☐ Swansea ☐ Neath Port Talbot ☐ Bridgend ☐ Rhondda Cynon Taff ☐ Merthyr Tydfil ☐ Blaenau ☐ Torfaen ☐ Monmouthshire ☐ Caerphilly ☐ Newport ☐ Cardiff ☐ Vale of Glamorgan ☐ Outside of Wales

Clear Form

1. Was a SNOD involved in the donation conversation?
☐ Yes ☐ No

2. Did the patient die in hospital in:
☐ Wales (go to Q3b) ☐ England (go to Q3)

3. For Welsh patients that died in England what was the patient registered decision on ODR? ☐ Opt In ☐ Opt Out ☐ Appoint Representative ☐ Not Registered

Would the patient have fitted deemed consent criteria if they had died in Wales? ☐ Yes ☐ No

Did the family think that deemed consent would apply?
☐ Yes ☐ No ☐ Uncertain ☐ Non Applicable (N/A)

Was consent obtained ? ☐ Yes ☐ No ☐ N/A

Had the family heard or read about the Act from the media campaign (Radio, TV, newspapers, postal leaflet etc)?
☐ Yes ☐ No ☐ Uncertain ☐ N/A

Were the family positive about the new Act?
☐ Yes ☐ No ☐ Uncertain ☐ N/A (go to Q.19)

3b. For Welsh patients who died in Wales, what was their registered decision on the ODR? ☐ Opt In ☐ Opt Out Appoint Representative ☐ Not Registered ☐

4. If patient was under 18 was their decision recorded on ODR?
☐ Yes ☐ No ☐ Non Applicable (N/A)

5. For patients of any age, did the family know of any other expressed decision?
☐ Yes ☐ No ☐ Uncertain ☐ N/A

6. Did the family agree with the patient's expressed donation decision? ☐ Yes ☐ No ☐ Uncertain ☐ N/A

7. Was there disagreement between one or more family members about the patient's expressed decision?
☐ Yes ☐ No ☐ Uncertain ☐ N/A

8. Was the patient's registered or expressed donation decision supported? ☐ Yes ☐ No ☐ N/A

*9. Only for patients who appointed a representative (AR).*

*Did the AR give consent?* ☐ *Yes* ☐ *No*

*Did the AR support new Act?* ☐ *Yes* ☐ *No* ☐ *Uncertain*

*Were the family aware of AR?* ☐ *Yes* ☐ *No* ☐ *Uncertain*

*Did the family agree with the decision of the AR?*
☐ *Yes* ☐ *No* ☐ *Uncertain* ☐ *N/A*

10. Did deemed consent apply? ☐ Yes ☐ No ☐ N/A

11. Did the family agree with the deemed consent of the patient?
☐ Yes ☐ No ☐ N/A
If no, did you feel able to get the family to support the deemed consent? ☐ Yes ☐ No ☐ N/A

12. Was there disagreement within the family and close friends involved in the deemed consent conversation?
☐ Yes ☐ No ☐ Uncertain ☐ N/A
If yes, did you feel able to get the group to support the deemed consent? ☐ Yes ☐ No ☐ N/A

13. Was consent obtained? ☐ Yes ☐ No ☐ N/A

14. Who consented? ☐ family member ☐ friend ☐ other ☐ N/A

15. Had the family heard or read about the Act from the media campaign (Radio, TV, newspapers, postal leaflet etc)?
☐ Yes ☐ No ☐ Uncertain ☐ N/A

16. Did the family have a correct understanding of the Act BEFORE you spoke to them? ☐ Yes ☐ No ☐ Uncertain ☐ N/A

17. Did the family have a correct understanding of the Act AFTER you spoke to them? ☐ Yes ☐ No ☐ Uncertain ☐ N/A

18. Had the new Act changed the family views of organ donation?
☐ Yes ☐ No ☐ Uncertain ☐ N/A
If yes, were they positive about the new Act? ☐ Yes ☐ No

19. Did any person suggest that they would register their decision in light of your conversation?
☐ Yes ☐ No ☐ N/A
If yes, would they: ☐ Opt in ☐ Opt out ☐ Appoint Representative ☐ Do nothing

20. Has this Act influenced your approach conversation with this family? ☐ Yes ☐ No ☐ Uncertain ☐ N/A

Anything else that could be helpful (e.g. Was there a pre-approach, a complaint or anything in this case that impacted upon you getting consent under the new law?)

Return by email to organdonationstudys@nhsbt.nhs.uk (for South Wales team) as soon as possible.

Submit
