# Supplementary material for: Family attitudes, actions, decisions and experiences following implementation of deemed consent and the Human Transplantation (Wales) Act 2013: mixed-method study protocol
Source: BMJ Open. 2017 Oct 12;7(10):e017287. doi: 10.1136/bmjopen-2017-017287 (PMC5652527; doi:10.1136/bmjopen-2017-017287)
Supplement: Supplementary data [file bmjopen-2017-017287supp004.pdf]

Overview of the Interview protocol

The interview provides an opportunity for the ‘family member’ to talk about their decisions and experiences of organ donation and deemed consent (if applicable) at a time that suits them after their bereavement.

The new way of consenting to organ donation came into law in Wales on the 1st December 2015. Families who have experienced a bereavement after 1st of December are the most affected by this change. The interview with the family enables greater clarity of the very personal experience of the new way of consenting to organ donation. The stories the family provide and the meanings they attribute to their beliefs, decisions and actions will support overall understanding of the ways the new Act impacts upon organ donation and transplantation in Wales.

Context for interviews

The interview will be semi-structured and available to families in Welsh or English through any mechanism the family chooses for example face to face, telephone, skype or social media.

The topic guide (see below) provides a loose structure to anchor the interview on the implementation of the Act. Participants will be free to talk in detail about their views, experiences and personal meaning in greater depth. This will elicit what the Act and its implications (such as registration on the organ donor register) and their behaviours and actions meant for them.

The interview guide will need adapting for each unique situation and the questioning will change depending on if the interviewee and others involved in the conversation supported the donation decision of their loved one or close friend or not.

Data arising from the interview will support and enrich the data analyzed in the SNOD & family questionnaires. Outcomes from the interview will help focus the study as it begins to reach conclusions from early analysis of data, and steer the remaining phases and support key findings.

English and Welsh will be analyzed separately and then brought back into the overall analysis.

Interview arrangement process

1. An agreed convenient date, time and venue will be agreed by the family’s preference.

2. The family will choose what format the interview will take for example face to face, telephone, skype.

3. The researchers will follow the distress protocol (in your pack) when interviewing.

4. Check all kit is working prior to interview

Taking consent

5. The researcher will provide an overview of the study to the family using the ‘overview’ above as a guide and referring to the study information booklet.

6. The researcher will explain the recording equipment briefly and how it will be used.

7. The researcher will explain how the interview will proceed.

8. The participants will be given opportunity to ask questions.

9. The researcher will ask family to fill out two copies of the consent to interview form (in your pack).

10. The researcher will provide participants with a copy of the signed consent form to keep.

11. After the interview, if considered appropriate, the researcher will share a pamphlet of CRUSE Bereavement Care Literature and explain who they are and how they can help.

Beginning the interview

12. Following the Distress Protocol - continue to observe for signs of stress before, during and after the interview

13. Discuss options of pausing or stopping the interview and remind family of why you are here for example 'I am here to listen to your vitally important stories. My input will be minimal i.e. 'less talking and more listening'. The experience should be informal and relatively relaxed. If it becomes anything else than this then it is time to pause and you must let me know.'

14. Start recording

15. Begin by stating date / time / location / and people present

*For Phone or other remote interviews consent will be obtained through the post ideally before the interview or as soon as possible after the interview. We cannot use the data given until written consent is obtained.

Introductory conversation:

Researcher should offer their condolences. Ask a bit more about them and ask a bit more about the person that died.

Say up front that there are 4 specific areas that you would like to cover in the interview:
1. General views about organ donation.
2. The new way of consenting to organ donation in Wales.
3. The media campaign promoting the new way of consenting to organ donation.
4. Their personal experiences of the organ donation process.

1. Topic area 1, general views about organ donation. (contextual questions)

1.1 - Have you had any previous experiences of organ donation before your loved one or close friend recently passed away?
1.2 - Do you have a personal view about organ donation?
1.3 - Has your personal view changed over time?
1.4 - Are you registered on the ODR? If so – what decision is registered?
1.5 - How do you generally feel about Organ Donation now after your loved one or close friend recently passed away?

Prompts:
Tell me more about..?
Can we go back to and talk further about?

2. Topic area 2, the new way of consenting to organ donation in Wales. (core questions)

'Changes to consenting in Wales came in on 1st December 2015. The law has changed to make it easier for people to donate their organs.'

2.1 - Are you normally resident in Wales or England?
2.2 - Had you heard about the new way of consenting to organ donation before your friend or loved one passed away?
2.3 - Can you explain in your own words what are the new changes to consenting to organ donation in Wales?

Probe and prompt as appropriate.

Researcher then follow with gently providing a clear context to facilitate clarification and further discussion:

'The new Law gives the decision about organ donation to the person to make during their lifetime. During their lifetime, people normally resident in Wales are asked to opt out or opt in to donation by registering a decision on the organ donation register or by discussing it with family and friends, or they can do nothing. If they do nothing and they meet specific criteria - it is assumed that they have no objection to being an organ donor. Citizens of Wales are actively encouraged to discuss their organ donation decisions with their friends and families. They can also appoint a representative to convey their decision on their behalf.'

2.4 - Is registering a decision on the organ donor register important to you, why?
2.5 - Did you know that you can register your decision through a conversation with a family member, not just by registering on the ODR?
2.6 - What does 'doing nothing' mean to you?
2.7 - Did you realise that 'doing nothing' is actually a choice? That is if you 'do nothing' then you support organ donation?
2.8 - What does deemed consent mean to you?
2.9- How do you feel about these changes to consent to organ donation now?
2.10 (If the deceased person was a child – did the new Act impact on their views and decision-making? If so how?)
2.11 (If the deceased person was normally resident in Wales and died in England - did the new Act impact on their views and decision-making? If so, how?)
2.12 If appropriate (for example if family are very positive about donation, proactively support donation are enthusiastic to express their views on the changes etc…) explore further families understanding of decision making especially deemed consent through 'what if' scenario. E.g. If you didn't know

the decision of your loved one would you have accepted deemed consent? Having been through the donation process would it be easier or harder to go through the donation process if you didn't know your loved ones wishes and consent was deemed, why? In your opinion does deemed consent have an equal status with a registered or a verbal opt in decision, why? In your opinion what needs to be improved in the changes to legislation?

Prompts
Tell me more about that…?
What was your understanding of…?
What does that mean for you…?
How do you feel about….?
You mentioned…could you tell me a bit more about that?

3. Topic area 3, organ donation media campaigns in Wales (core questions)

'Thank you, can we move on and focus on hearing more about what you saw in the media about the changes to consenting to organ donation in Wales prior to 1st December.'

3.1 - Had you seen any of the organ donation advertising campaign (television advert, billboards, bus campaign, every home in Wales got several letters, emails etc.)?
    3.1(a) - If so which advertising material had you seen?
3.2 - Can you remember the key messages from the advertising material?
3.3 - What did you think about the messages?
3.4 - Did you understand the key messages?
3.5 - What did you do as a result of seeing the advertising campaign?'

Prompt
Focus specifically on their actions to the media: opt in, opt out on ODR, do nothing with assumption of agreement to organ donation, discuss donation and donation decision with family, appoint a representative.

Following this discussion about what they remembered the researcher should now gently show the interviewee some of the organ donation changes advertising materials (from interviewer pack). These props may help to further frame this question and further jog memory. Note which one interviewer showing to assist transcription).

Focus again specifically on their actions as a result - Opt in, opt out, do nothing, discuss with family, appoint a representative.

Prompts:
Do you remember seeing any of these?
What did you think when you saw it first?
What would have made that difference to make you notice it more?
What do the messages mean to you now?
Can we go back to and talk further about…?

4. Topic guide 4, their detailed experience of the organ donation process.

Specific questions for family members and close friends involved in the organ donation approach conversation with professionals.

4.1 Establish which elements of consent applied in this case:

Prompts
Had your loved one or close friend registered an organ donation decision on the ODR (ask if opt in or opt out?)
OR Did your loved one or close friend 'do nothing'?
Had your loved one or close friend expressed an organ donation decision during their lifetime?
If so what did they say and to whom?
Did Deemed consent apply? (explain deemed consent again if appropiate)

(Be specific as to what deceased persons donation decision was - Opt in opt out, expressed decision during lifetime, do nothing and assume consent, appoint AR)

4.2 - Do you know if (deceased person's) view changed over time?
4.2(a) If yes, explore fully when and why.
4.3 - What was the organ donation decision? Consent or no consent?
4.3(a) If decision different to deceased persons decision,
how did you go about overturning the decision?
4.4- How well prepared did you feel for how very ill your relative really was?
4.5 - Did anyone talk to you about palliative or end of life care for your relative or close friend?
4.6 - When did you realise that they may not survive?
4.7 - In terms of NHS processes what helped at this time?
4.8 - Is there anything else that might have helped you?

Prompts
How did that make you feel?
Can you tell me more about those moments?
You mentioned…can you tell me more about it?
Can we go back to and talk further about…?

4.9 - Can you remember your first thoughts when you were approached about organ donation when your loved one was very ill?
4.10 - Who first approached you about organ donation (doctor, nurse, SNOD)?
4.11 - How did the organ donation conversation play out in your mind?
4.12 - Who was involved in the donation discussion (e.g. Family members, friends, others)
Prompts, focus on role of SNOD, roles of local clinicians. Unpack how many meetings/conversations were had and with whom.

4.13 - Did you feel that you could express your views about your loved ones organ donation decision during the conversation?
4.14 - Did anyone or anything influence your view on organ donation and your relative/close friend?
4.15 - Other than the professionals - who did you talk to in detail about your relative/close friend and organ donation?

Prompts
Unpack other family/friend & other positive and negative influences

4.16 - Was there agreement amongst those involved?
4.16(a) - If there was disagreement – what was the disagreement about?
4.16(b) - Was the disagreement resolved and how?
4.17- In the end, did everyone support the donation decision of your loved one or close friend?
4.17(a) - If not, whose opinion counted most?
4.18 - Was it easier just to say no/yes?
4.19 - Who conveyed the decision to the professionals?

4.20 - Is there anything else you think that the professionals could have helped you with in this situation?

Prompts
How did you feel about it?
Can you tell me more about…?
Can we go back to where you mentioned and talk further about…? *(Unpack if there were any specific cultural influences that impacted on the role and influence of the interviewee on the decision-making process)*

4.21 - **If deceased persons decision was changed -**
– 'For you, was the primary consideration more about making things easier for you and the family looking forward, rather than honouring the donation decision of your loved one or close friend?'
4.22(a) - Do you think there are any negative consequences and impacts of not supporting the deceased person's decision.
4.22(b) - Do you think there are any positive consequences and impacts of not supporting the deceased person's decision.
(unpack if interviewee would act in the same way now as they did then)

Signal disengagement and end of interview coming up.

4.22 - Is there anything else you would like to share that hasn't been covered?
4.23 – Finally, would you accept a transplant if you needed one?

'This has been very valuable'
'Your insights and stories have really helped'

Leave time for an open discussion.

Bring discussion back to a positive place to conclude the interview.

Thank you very much. We have come to the end of the interview.

Check that you have covered/know the following details

Record of conversation with:
spouse or partner   parent or child   brother or sister   grandparent or grandchild   niece or nephew   stepfather or stepmother   half-brother or half-sister   friend of long standing
Age Range of Interviewee   0-18   19-35   36-50   51-70   >71
Age Range of Deceased Person   0-18   19-35   36-50   51-70   >71
Number of people present at interview
Time passed since death
Was death expected
Area where interviewee resides
Area where person died
Area where deceased person resided
Was deceased person BME

Post Interview Arrangements

16. Researcher should thank the interviewee for their time

17. Give the interviewee contact details (on the back of the information booklet) should they wish to follow anything up.

18. Ask if they can follow up the interview with a courtesy phone call in the near future

19. Reintroduce the support literature while preparing to leave.

Uploading and storing data

20. Researcher should upload data to ‘U: Drive’ ASAP after the interview
21. Ensure data is properly uploaded after the interview before deleting from recording device.
22. Begin transcribing ASAP after the interview.
23. Assign a study code at point of transcription.

Follow up protocol

24. Researcher should follow up with a courtesy call after 6 weeks from date of interview (if permission given).
25. Researcher should assess if family member feels satisfied that they have communicated everything they wanted to.
26 If family member still wants to give their views to researcher, researcher should explore other options such as a quick conversation there and then on the phone, or a follow up email correspondence. Researcher should also if appropriate direct family member to CRUSE bereavement care for long term professional support.

* Researchers should follow protocols where relevant set out in Safety Code of Practice, Lone Working Policy & Field Work Safety Documents available from Bangor University and the project folder on University U Drive.
