# Supplementary material for: Family attitudes, actions, decisions and experiences following implementation of deemed consent and the Human Transplantation (Wales) Act 2013: mixed-method study protocol
Source: BMJ Open. 2017 Oct 12;7(10):e017287. doi: 10.1136/bmjopen-2017-017287 (PMC5652527; doi:10.1136/bmjopen-2017-017287)
Supplement: Supplementary data [file bmjopen-2017-017287supp005.pdf]

Appendix 5. Ethical issues and strategies

Undertaking research with vulnerable participants: Grieving families, close friends and appointed representatives are vulnerable and considered to be most affected by the Act. It appears appropriate, with the right support and safeguards in place, to provide an opportunity for them to participate in a study to elicit their perspectives. Our patient representatives have written a letter of support indicating that families will want the option to be able to talk to someone outside of the NHSBT Team, to share their positive or negative views about how their role has changed under the new Act and whether they agreed with the decision of the deceased person or not.

Although they have been recently bereaved, potential donor families, close friends and appointed representatives may want an opportunity and may benefit from expressing their views, which in turn may inform development of practice in a continuous improvement cycle. To ensure that an appropriate approach is made to potential participants at an appropriate time, we will ask the Specialist Nurses in Organ Donation (SNODs) to use their judgement as to the most appropriate time to share information on the study. SNODs will have spent a lot of time with the people involved in the donation conversation and can use their professional judgement to select from a range of methods to recruit participants that are individually tailored for each situation, including: via direct contact with families by SNODs; by sending study invitation and information attached to routine follow up communication by SNODs; by direct mailing of study invitation and information by NHS Blood and Transplant. In addition we will place three adverts in the national media at staged intervals and recruit through snowball sampling. If a family member, close friend or appointed representative knows of another person involved in the organ donation process who may want to be interviewed, we will ask them to share a letter of invitation and study information with them and ask the person to contact us directly.

We will follow the sensitive ethical framework[2] and practical strategies shown in Table 1. Participants who return self complete anonymised questionnaires and who wish to themselves remain anonymous will be made aware that returning a completed questionnaire to the research team will constitute consent to use the data. For all other research procedures such as interviews informed written consent will be required. All participants will be over 16 years with mental capacity to consent. Participants can choose to be interviewed at any point that is appropriate and convenient for them after their bereavement up until the end point of data collection. If an individual receives more than one letter of invitation we will include a sentence to explain that, if they have already made their decision whether to participate in the study or not, to ignore the letter or to pass the invitation on to another family member or close friend of the deceased person, because NHSBT only have one contact name for each case. Researchers will follow a ‘Distress Protocol’ (see below) when conducting interviews and if participants become unduly distressed they will use the three step approach in the protocol to safeguard the wellbeing of participants.

CRUSE Bereavement Care Cymru aim to reach out and support all bereaved people in Wales. We will share their client information with participants and the contact numbers for CRUSE bereavement care and support. If the research officer has any serious concerns

about the safety of any participant in the study, they will follow the standard protocol of NHSBT for Safeguarding Vulnerable People.

Anonymity of Professionals: Interviews with professionals will be conducted at a venue of their choice, and they will be assigned a code. Where there is only one role in the organisation care will be taken not to identify participants by using their quotes without permission. Participants will be offered a choice of 1:1 or small group or focus group interviews if they do not wish their experiences to be shared with colleagues.

Support for researchers undertaking sensitive interviews: The research team is large (5 core members) to provide a mutually supportive context and debriefing will be offered after interviews. Researchers have been recruited with skills and experience of undertaking interviews on sensitive topics with vulnerable people. The research team is also working in partnership with NHSBT teams who provide an additional supportive environment and peer to peer support and mentorship concerning sensitive issues, should research team members need it. We have built in three joint professional development opportunities for support, shared learning and reflection. Researchers can also contact CRUSE bereavement care for additional confidential and independent support.

Good clinical practice. Core research team members have undertaken Good Clinical Practice in research training and those who will have contact with family members have been subject to screening under the Disclosure and Barring Service.

Data Protection and Data Sharing Agreement

The research requires anonymised data to be collected and shared between NHSBT and Bangor University. Bangor University has entered into a standard data sharing agreement with NHSBT. Data collected directly by the research team will be recorded on encrypted digital recorders, regularly downloaded, assigned a unique code and transcribed, anonymised and stored securely on password protected university servers and laptops. Data on digital recorders will then be deleted. Any paper based patient identifiable information will be stored under lock in a secure place with access controlled by the research team.

The following process has been previously adopted in other studies to share non-patient identifiable information. The process is designed to enable the research team at Bangor University to have access to anonymised information recorded by SNODs that sheds light on decision-making by families/appointed representative(s) (especially when they say no to organ donation). Patient and Public representatives from partner non-government organisations who support bereaved families have been consulted and support this approach. The structured communication process used by SNODs during the donation request stage will be amended for introduction of the new Act and documented in NHSBT Standard Operating Procedures and Management Process Descriptions. The ‘approach’ conversation is highly structured and follows a ladder of issues for families to think about, consider and discuss. The SNOD is trained to explore and clarify any issues or questions that are raised and to facilitate and listen to reasons and concerns when considering giving or declining consent to donation. To capture this information in a structured and anonymised way that falls outside of the Data Protection Act, we have agreed a plan with the two NHSBT donor teams covering Wales, to jointly develop a standard proforma for SNODs to record

key anonymised information from the ‘approach’ conversation for research purposes as soon as practical after it has concluded and once they have disengaged from the family. The anonymised information recorded on the proforma will be returned electronically to the researchers in batches so that it is not possible to link any single proforma with any specific death. We will also be respectful of professional anonymity and the proformas will be designed in such a way that the identity of the hospital or person who completed it will not be known to researchers.

Table 1. Study framework for ethical decision-making.[2]

Ethical considerations	Practical strategies
	**Participant identification and recruitment**
Access, confidentiality Regard	Formally obtain the support of a key person to undertake the role of identifying potential participants and disseminating pre-prepared recruitment packs on behalf of the research team.<br>Recruit potential participants in a serial manner, for example, send out a maximum of five recruitment packs at any one time so that participants are not kept waiting for long periods before the research interview.
Respect, relevance	Consider participant inclusion criteria of bereaved no less than 3 months and no more than 12 months at the time of recruitment to the study. * *We will ask Specialist Nurses in Organ Donation to share study information at the time of bereavement and will offer the option of a self-complete questionnaire that involves no face to face contact with researchers and an interview at a time when the participant feels that it is appropriate for them. We have built in a 3 month time lag to collect data after the last participant has been contacted.*
Compassion	Include a covering letter that introduces the study in a personalised way by taking familiarity into consideration.
Informed choice	Provide clear written and web-based information about the researchers and the study.<br>Include an invitation to contact the researcher.<br>Demonstrate timely responsiveness to any potential questions or queries.
Non-coercion	Provide a minimum of 10 days for participants to decide about joining the study.
	**The research interview**
Choice, respect	Agree a convenient date, time and venue for the research interview. Avoid dates that coincide with any significant family events or anniversaries.
Safety	Implement a study site policy for researchers working alone in advance of the interview encounter.
Safety, support	Competent researcher with experience of conducting sensitive research interviews and supporting the bereaved.
Choice, privacy	Provide the option of an interview face to face or remotely, for example, via telephone.
Informed consent	Provide an overview of the study and present opportunity for participants to ask questions. Explain how the interview will proceed. Obtain written agreement to audio-record the interview and to use anonymous quotes in any presentation of the research. Provide participants with a copy of the signed consent form to keep.
Support	Discuss and agree avenues of post-interview support prior to the interview commencing<br>Observe/listen for signs of distress during the interview.<br>Discuss the option of pausing the<br>recording or stopping the interview. Plan a natural break for refreshments.
Confidentiality, anonymity	Ensure audio-recordings and transcripts are securely stored and electronic data are password<br>protected. Assign a study code at the point of transcription.
	**Post-interview follow-up care**

Support	Arrange a convenient time to telephone the participant (normally in 24–48 h) to check on any issues the interview may have raised and to answer any questions.<br>Compile information about local support organisations. Offer this to participants if they consider it helpful and/or direct them to appropriate professionals to discuss any issues of concern.<br>Establish if participants wish their general practitioner (GP) to be informed about their participation in the study and obtain written consent to proceed. Provide GP with information about the study at the time of notification.
Appreciation	Send participants a personal thank-you letter and offer an executive summary of the research findings.
Involvement	Provide participants with an opportunity to evaluate their experience of participating in bereavement research.
Researcher Support	Determine support for the researcher from an individual with whom they feel comfortable and who is suitably qualified to provide support. Plan a debriefing session after each interview encounter. Utilise reflexive notes to guide the discussion.

Distress Protocol

During instances of bereaved participants becoming distressed during the interview process, the subsequent protocol will be followed.

Identifying Distress

The interviewer will be mindful of signs of distress in the participants throughout the interviews. Signs of distress to look out for will include:

- Exhibition of behaviours that indicate that the discussion has become too upsetting for them, including crying and an inability to continue for example.
- The participant verbally communicating that they are experiencing distress during the interview.

Response Stage 1

- The interview will be stopped.
- The participant will be offered a break, have a drink of water/ tea etc.
- The participant will then be asked if they would like to continue the interview or if they would prefer to discontinue. Should they wish to go on, the interview will resume.

Response Stage 2

If the participant elects to discontinue the interview,

- The interview will not continue.
- The interviewer will signpost where support can be obtained such as from the SNOD or the bereavement support service at the hospital where their relative/friend died, or from their GP.
- The participants will also be reminded again of the contact details for Cruse Bereavement Care,' an organisation which provides support for bereaved people.

Response Stage 3

- At a later date, the interviewer will follow up with the participant with a courtesy call (with the participant's consent). If the participant feels strongly that they would still like to have their views and experiences heard – the interviewer will go through options (wait a while before rearranging, explore other methods rather than face to face etc.

References

1. The Data Protection Act 1998. Reviewed March 2017 http://www.legislation.gov.uk/ukpga/1998/29/contents (Accessed March 2017).
2. Sque M. Walker W. and Long-Sutehall T. (2014) Research with bereaved families: A framework for ethical decision-making. Nursing Ethics doi:10.1177/0969733014521097.
