# Supplementary material for: Family attitudes, actions, decisions and experiences following implementation of deemed consent and the Human Transplantation (Wales) Act 2013: mixed-method study protocol
Source: BMJ Open. 2017 Oct 12;7(10):e017287. doi: 10.1136/bmjopen-2017-017287 (PMC5652527; doi:10.1136/bmjopen-2017-017287)
Supplement: Author's manuscript [file bmjopen-2017-017287.draft_revisions.pdf]

BMJ Open is committed to open peer review. As part of this commitment we make the peer review history of every article we publish publicly available.

When an article is published we post the peer reviewers' comments and the authors' responses online. We also post the versions of the paper that were used during peer review. These are the versions that the peer review comments apply to.

The versions of the paper that follow are the versions that were submitted during the peer review process. They are not the versions of record or the final published versions. They should not be cited or distributed as the published version of this manuscript.

BMJ Open is an open access journal and the full, final, typeset and author-corrected version of record of the manuscript is available on our site with no access controls, subscription charges or pay-per-view fees (http://bmjopen.bmj.com).

If you have any questions on BMJ Open's open peer review process please email editorial.bmjopen@bmj.com

BMJ Open

Family attitudes, actions, decisions and experiences following implementation of deemed consent and the HumanTransplantation (Wales) Act 2013: Mixed-method study protocol

Journal:	*BMJ Open*
Manuscript ID	bmjopen-2017-017287
Article Type:	Protocol
Date Submitted by the Author:	12-Apr-2017
Complete List of Authors:	Noyes, Jane; School of Social Sciences<br>Morgan, Karen ; Welsh Government , Major Health Conditions Policy Team, Directorate of Health Policy, Health and Social Services Group, Welsh Government, Cardiff, Wales, UK.<br>Walton, Philip; NHS Blood and Transplant , Organ Donation Department, Unit 3 Cae Gwyrdd, Greenmeadow Springs Business Park, Tongwynlais, Cardiff, UK.<br>Roberts, Abigail; NHS Blood and Transplant<br>Mc Laughlin, Leah; School of Social Sciences<br>Stephens, Micheal; Cardiff and Vale University Health Board, University Hospital of Wales, Department of Nephrology and Transplantation, Heath Park, Cardiff, UK.
**Primary Subject Heading**:	Health services research
Secondary Subject Heading:	Research methods, Surgery, Nursing
Keywords:	Change management < HEALTH SERVICES ADMINISTRATION & MANAGEMENT, Health policy < HEALTH SERVICES ADMINISTRATION & MANAGEMENT, Organisational development < HEALTH SERVICES ADMINISTRATION & MANAGEMENT, Transplant medicine < INTERNAL MEDICINE, LAW (see Medical Law), Renal transplantation < NEPHROLOGY

SCHOLARONE™
Manuscripts

TITLE PAGE

Family attitudes, actions, decisions and experiences following implementation of deemed consent and the HumanTransplantation (Wales) Act 2013: Mixed-method study protocol

Corresponding Author: Jane Noyes,
Address: Room 107, Neuadd Ogwen, School of Social Sciences, Bangor University, Wales, LL57 2DG, UK
Email: jane.noyes@bangor.ac.uk
Phone:+44 (0) 1248 388519

Authors
Jane Noyes[1]
Karen Morgan[2]
Phillip Walton[3]
Abigail Roberts[4]
Leah Mclaughlin [1]
Michael Stephens[5]

1. School of Social Sciences, Bangor University, Bangor, Wales, UK.
2. Major Health Conditions Policy Team, Directorate of Health Policy, Health and Social Services Group, Welsh Government, Cardiff, Wales, UK.
3. NHS Blood and Transplant, Organ Donation Department, Unit 3 Cae Gwyrdd, Greenmeadow Springs Business Park, Tongwynlais, Cardiff, UK.
4. NHS Blood and Transplant, North West Regional Office, 14 Estuary Banks, Speke, Liverpool, UK.
5. Cardiff and Vale University Health Board, University Hospital of Wales, Department of Nephrology and Transplantation, Heath Park, Cardiff, UK.

Abstract

Introduction

The Human Transplantation (Wales) Act 2013 (The Act) introduced a ‘soft opt-out’ system of organ donation on 1.12.15. Citizens are encouraged to make their organ donation decision known during their lifetime. In order to work, the Act and media campaign need to create a context whereby organ donation becomes the norm, and create a mechanism for people to behave as intended (formally register their decision; consider appointing a representative; convey their donation decision to their families and friends, or do nothing – deemed consent). In addition, family members/appointed representatives need to be able to put their own views aside to support the decision of their loved one. The aim of this study is to evaluate initial implementation, outcomes, and impact on families and appointed representatives who were approached about organ donation during the first 18 months.

Methods and analysis

Prospective mixed-method co-productive study undertaken with National Health Service Blood and Transplant (NHSBT), and multiple patient/public representatives. The study is designed to collect

For peer review only - http://bmjopen.bmj.com/site/about/guidelines.xhtml

information on all cases who meet specified criteria (≥18 yrs, deceased person normally resident in Wales and died in Wales or England) whose family were approached between 1/12/15-31/6/17). Data for analysis includes: NHSBT routinely collected anonymised audit data on all cases; Specialist Nurse in Organ Donation (SNOD) completed anonymised form for all cases documenting their perception of the families' understanding of the Act, media campaign, and outcome of the donation approach; Questionnaires and depth interviews with any family member or appointed representative (minimum 50 cases). Additional focus groups and interviews with SNODs. Anonymised donation outcomes and registration activity reports for Wales provide additional context.

Ethics and dissemination

The study is informed by an ethical framework for undertaking research in this context. CRUSE Bereavement Care Cymru are suppling information for bereaved families. Multiple dissemination strategies will be employed.

Registration

The protocol is registered on the Health and Care Research Wales Clinical Research Portfolio. Study ID number 34396, www.ukctg.nihr.ac.uk

Key Words: Law, Organ Donation, Implementation, Mixed-method evaluation, Deemed Consent, Wales

Word count 4726

Strengths and limitations of this study

- The study is a large scale prospective mixed-method evaluation of the immediate impact of the Act using multiple data sources.
- NHS Blood and Transplant is an equal partners in this co-productive study.
- Many Patient and Public representatives and organisations are supporting the study.
- CRUSE Bereavement Care Cymru is supplying bereavement support information for bereaved families who participate in the study.
- Numerous strategies will be used to disseminate findings.

For peer review only - http://bmjopen.bmj.com/site/about/guidelines.xhtml

Family attitudes, actions, decisions and experiences following implementation of deemed consent and the Human Transplantation (Wales) Act 2013: Mixed-method study protocol

INTRODUCTION

The Human Transplantation (Wales) Act 2013 introduced a 'soft opt-out' system of organ donation.[1] The purpose of the Act is to make it easier for people to donate their organs to benefit patients. The Act is central to the Wales Action Plan[2], which sets out a programme of continuous improvement on all aspects of organ donation and transplantation to deliver the NHS Blood and Transplant (NHSBT) strategy 'Taking Organ Transplantation to 2020'.[3] NHSBT is a Special Health Authority in England and Wales (accountable to the Department of Health) that is responsible for promoting tissue and organ donation to the public and managing organ donation and transplantation. The overall target of the strategy is to increase United Kingdom (UK) consent rates to 80% by 2020. Under the former 'opt-in' system, in 2012/13, 2013/14 and 2014/15 only 50.3%, 53.6% and 48.5% of families consented to deceased donation in Wales.[4] In contrast the consent rate in Spain, which operates an 'opt-out' system in which all citizens are automatically registered for organ donation unless they choose to state otherwise, ranged between 80-85%.[3,5.]

The Act constitutes one of the biggest changes to the partnership and social contract between the Welsh Government and the people of Wales. The Act is however controversial and not everyone consulted agreed with the 'soft opt-out' system and its principle of deemed consent.[6-7] Potential donor families are considered to be most affected by the Act as, unlike the old 'opt-in' system, their role in the 'soft opt-out' system remains essential but changed by deemed consent.[8] Under the previous 'opt-in' system, which came under the Human Tissue Act 2004[9], if the individual's consent had not been indicated by the deceased person or a nominated representative, consent was sought from the person who was in a 'qualifying relationship' with the deceased person immediately before their death (usually a family member). If the decision regarding donation was unknown then families were less likely to give consent.[8,10] If those close to the deceased person objected to organ donation, for whatever purpose, when the deceased person (or their nominated representative) had explicitly consented, they did not have the legal right to revoke the consent, however the existence of appropriate, valid consent permitted donation to proceed, but did not mandate that it must. The final decision about whether to proceed rested with the medical team when family members did not support donation.

For peer review only - http://bmjopen.bmj.com/site/about/guidelines.xhtml

How the intervention is intended to work

In a research context the Act and implementation strategy is conceptualised as a complex behaviour change intervention.[11] The Act changes the principles of consent to deceased organ donation from one of 'opt-in' to a 'soft opt-out' for adults who are 18 years or over; voluntarily resident for 12 months or more in Wales; who has not made an express decision regarding organ donation; and is competent to understand the notion of deemed consent. The individual must also die in Wales for the Act to apply.

NHSBT employ teams of Specialist Nurses in Organ Donation (SNODs) who work across regions to support the organ donation process. The choices individuals now have in either expressing their organ donation decision or choosing to do nothing and having their consent deemed (criteria apply) have impacted on the approach to the family by the SNODs. Once the SNODs have ascertained that the individual has not recorded their organ donation decision on the Organ Donor Register (ODR) and has not appointed a representative to make the decision on their behalf, the conversation with the family is presumptive in favour of organ donation, informing them if applicable their relatives consent will be deemed to have been given. During the conversation the family are able to inform the SNODs that their relative did not want to be an organ donor. In this circumstance the family are required to produce clear evidence that the person did not want to be an organ donor. The Act is permissive in the sense that it allows for consent to be deemed in certain circumstances, however it does not mandate that organ donation goes ahead in such cases. If an individual has registered a decision or informed someone that they did not want to donate organs prior to their death, their decision will be respected unless the family is able to produce clear evidence that the individual had changed their mind.

Intended behaviour change

The success of the Act depends on behaviour change (public and professional) to work as intended. The theory is that the neutral media campaigns supporting implementation will facilitate five behaviours:

(1) People will register to 'opt-in' on the organ donor register and appoint a patient representative,

(2) or they register to 'opt-out';

(3) People will discuss their donation decision with families and friends;

(4) People can do nothing and it will be assumed that they do not object to organ donation (deemed consent)

(5) in making the donation decision, families will put aside their own views on donation and respect the decision of the deceased person.

For peer review only - http://bmjopen.bmj.com/site/about/guidelines.xhtml

Overall this complex intervention addresses four components of behaviour change as outlined in the Nuffield Council of Bioethics ladder of intervention (Figure 1).[12] The Act and implementation strategy were designed to change the default position so that organ donation became the norm. The Government-led media campaign was however presented in a neutral way to provide people with information to make an informed choice. Nudge theory was also used to underpin behaviour change – such as exposing the population of Wales to a series 'nudge alerts' via email, Royal Mail, and the media to do specific things such as making their organ donation decision known and 'opting in' or 'out' on the organ donation register.[12] The media did however generally present organ donation as having positive benefits (eg: giving the gift of life).

In addition to the public media campaign, there was an accompanying implementation strategy for NHS and NHSBT staff, which required amending clinical protocols and procedures and retraining large numbers of staff and all SNODs covering Wales. The multiple elements of this complex intervention are shown visually in Figures 2,3,4.

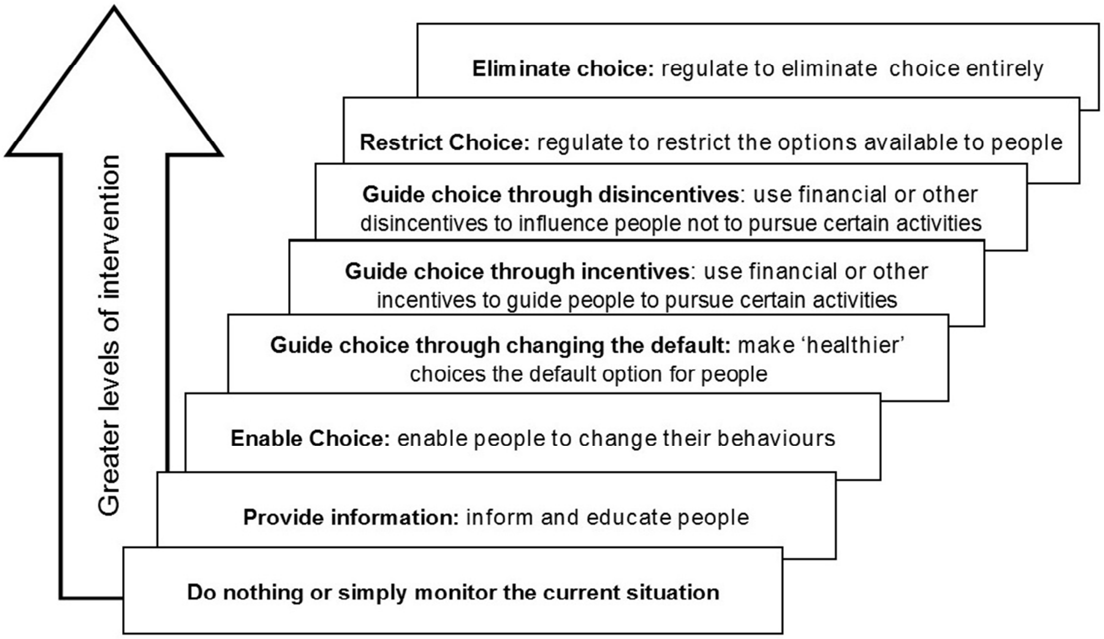

Figure 1. Redrawn from original. Nuffield Council of Bioethics ladder of intervention.[12]

For peer review only - http://bmjopen.bmj.com/site/about/guidelines.xhtml

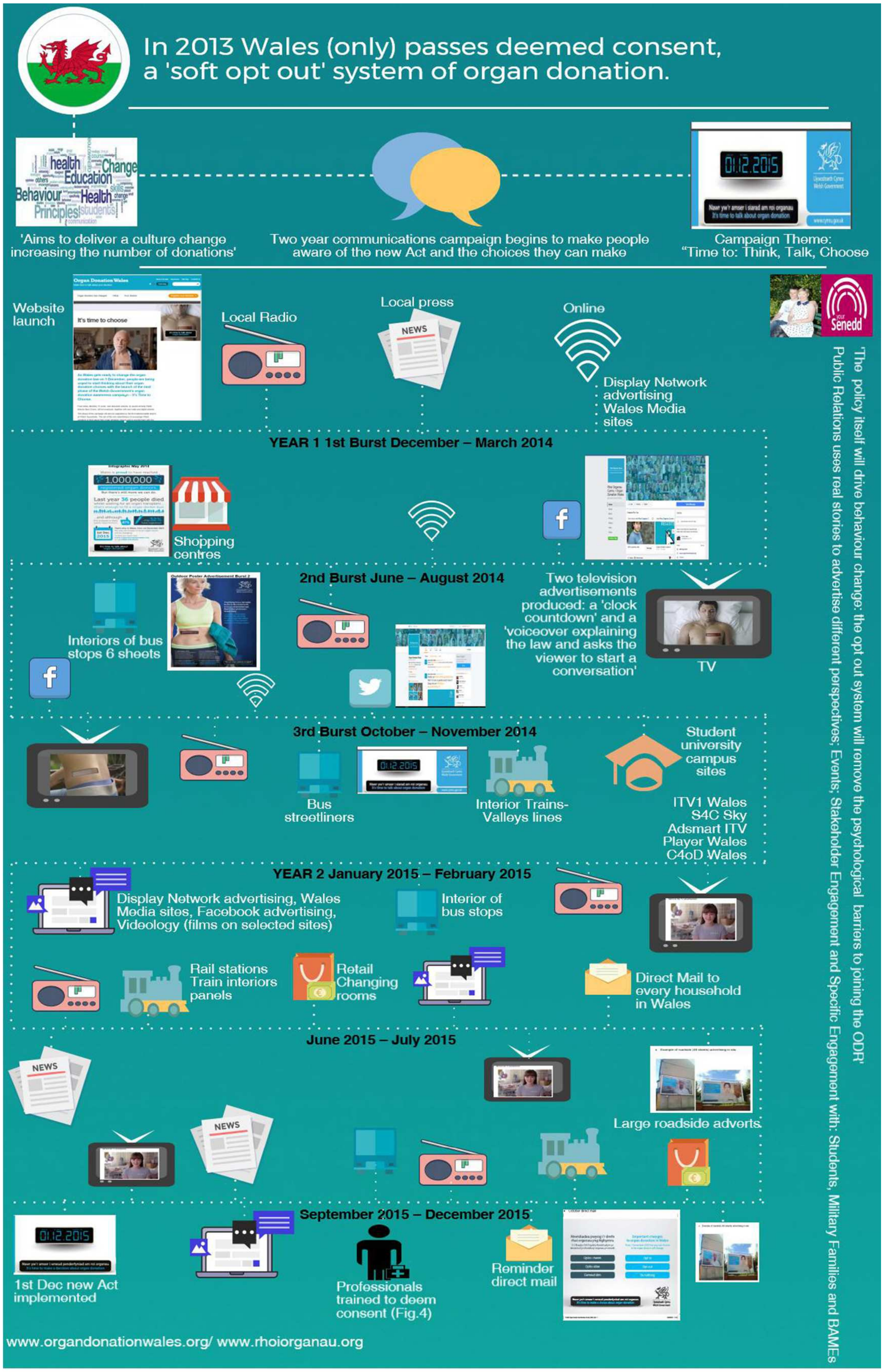

Figure 2. Intervention implementation: Multi-facetted media-based strategy to inform the public of the Act and changes to consenting to organ donation.

For peer review only - http://bmjopen.bmj.com/site/about/guidelines.xhtml

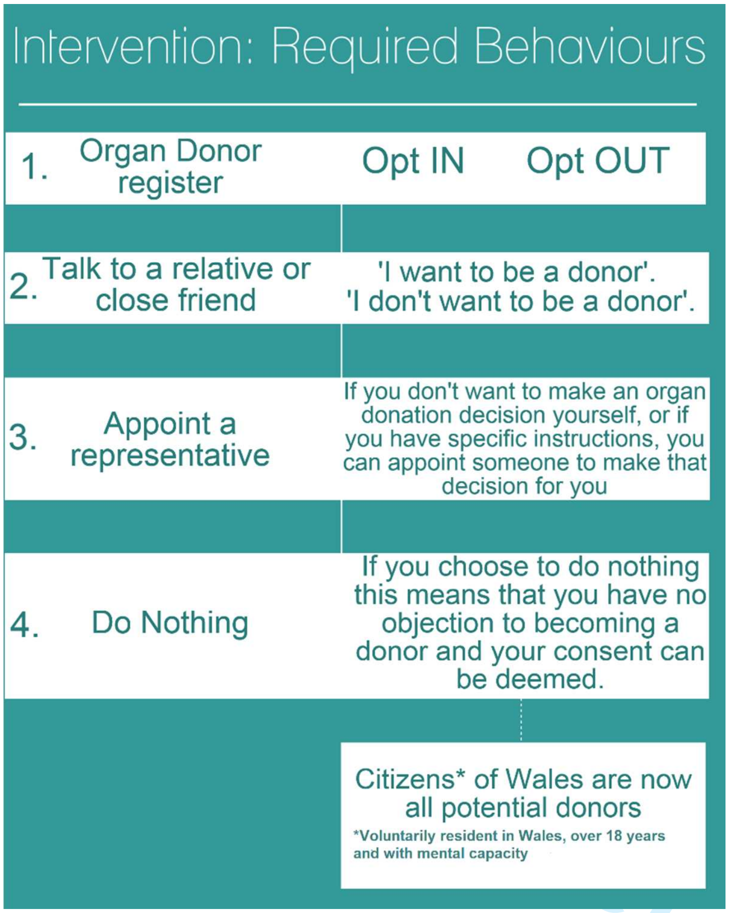

Figure 3. Intervention: Required behaviours of the public following introduction of the Act.

For peer review only - http://bmjopen.bmj.com/site/about/guidelines.xhtml

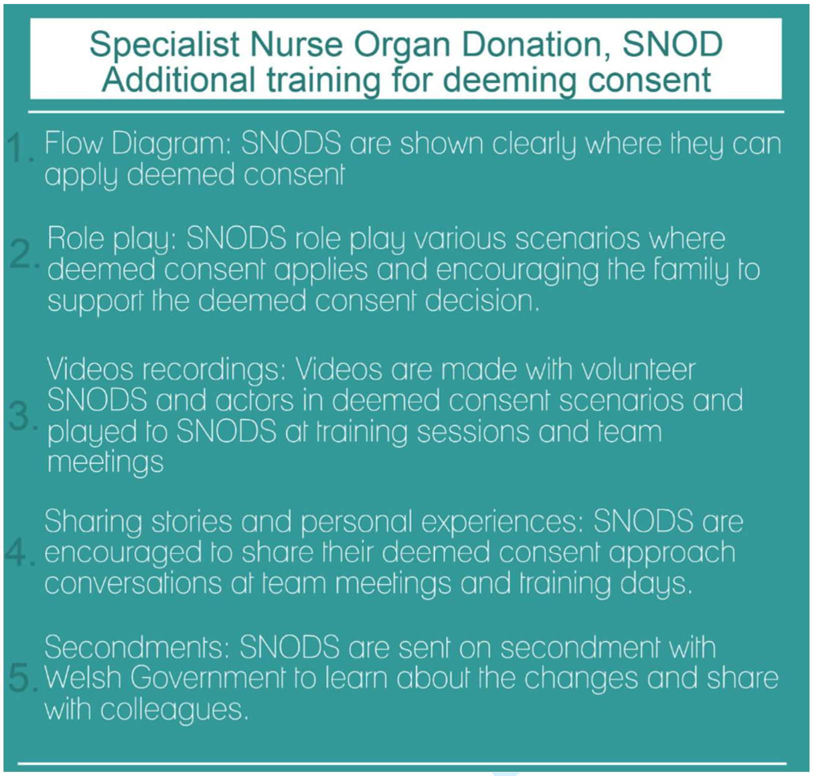

Figure 4. Intervention implementation: Additional training for Specialist Nurses in Organ Donation when approaching families following the implementation of deemed consent. See also Figure 1.

Modifications to the approach conversation under the Act.

The SNOD facilitates an approach conversation with the family at the point indicated in Figure 5.

For peer review only - http://bmjopen.bmj.com/site/about/guidelines.xhtml

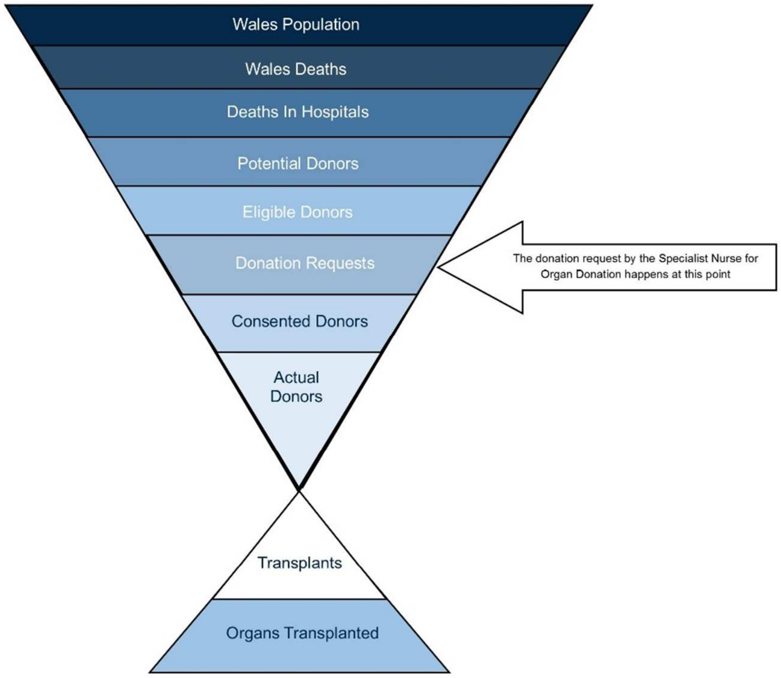

Figure 5. Wales potential organ donor population and identification of the ‘donation request’ stage in the process.

After the 1st December 2015, for deceased people who have not registered to ‘opt-out’ on the ODR, the approach to families will be a presumptive conversation in favour of organ donation. The sequence of obtaining consent for deceased organ donation when the patient has not recorded their decision t on the ODR is shown in Figure 6. Irrespective of whether the deceased person is registered on the ODR or not - the assumption is that family members will put aside their own beliefs if different to the deceased person and support the express decision to donate or by choosing not to register a decision by any means support their relative’s deemed consent.

For peer review only - http://bmjopen.bmj.com/site/about/guidelines.xhtml

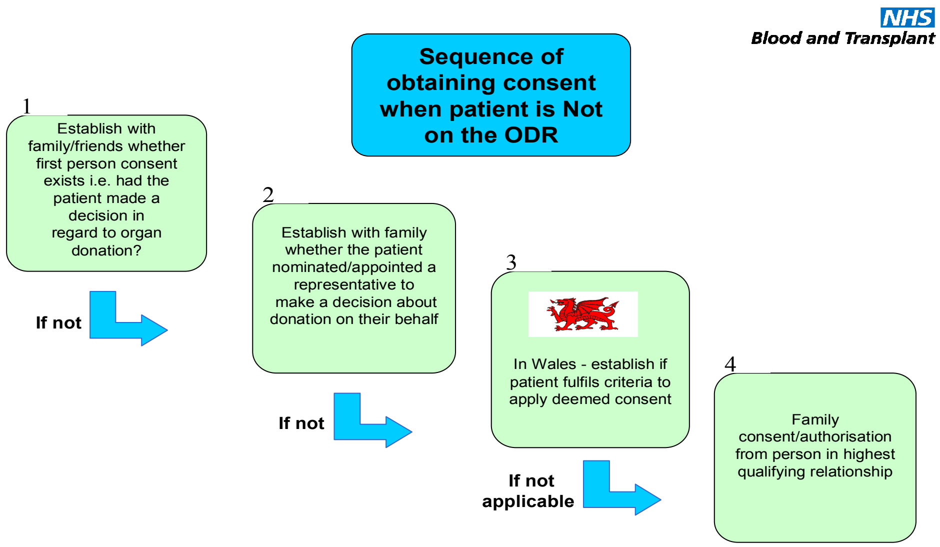

Figure 6. Sequence of obtaining consent when the patient is NOT on the Organ Donor Register (ODR) after 1.12.15. Image reporduced from NHSBT document using 'Fair use' clause.

Prudent healthcare principles

The Act is conceived as a Prudent healthcare policy. Any Prudent health service or intervention is based on the following 4 principles:[13-14]

- Achieve health and wellbeing with the public, patients and professionals as equal partners through co-production. Patient and public contribution is essential to create a patient-centred system for both potential donors and transplant recipients.13-14The soft opt-out system has been developed in close consultation with the people of Wales.[15-18]
- Care for those with the greatest health need first, making the most effective use of all skills and resources. The principles underpinning organ transplantation decisions are founded on caring for those with the greatest health need first, irrespective of ability to pay. There is good evidence that all transplants are cost-effective. For example, the cost benefit of kidney transplantation compared to dialysis over a period of ten years (the median transplant survival time) is £241,000 or £24,100 per year for each year that the patient has a functioning transplanted kidney.[19] Although the Act covers all organs and tissues from which patients may benefit from cost-effective transplants; the case for economic renewal and regeneration is best made in Wales by increasing the number of kidney transplants. Kidney transplants are highly cost-effective particularly in relation to NHS spend, and is the treatment of choice for many patients with end-stage renal failure. Recipients can often engage more productively in the economy once they no longer need dialysis.

For peer review only - http://bmjopen.bmj.com/site/about/guidelines.xhtml

- Do only what is needed, no more, no less; and do no harm. The 'soft opt-out' is designed to make it easier for the people of Wales to become organ donors. Transplantation is designed to offer patients more options for their treatment with increased benefits that outweigh the risks.
- Reduce inappropriate variation using evidence based practices consistently and transparently. Attitudes to organ donation vary across Wales and across social gradients and cultures.[18] The purpose of the neutral media campaign is to reduce this variation by providing the public with high quality accessible information.

Rationale for the study

There is evidence from a UK context describing the multiple converging factors that appear to influence donation decisions under the 'opt-in' system, such as knowledge of the deceased's wishes and the view of families that the deceased person had suffered enough.[13-18] We want to specifically explore the perspectives of organ donor registration and deemed consent with families and close friends who were involved in an organ donation decision.

This study is designed to address a critical gap in understanding by exploring if the Act has changed the views and decisions of families. The research is needed to understand donor family responses, which could have an immediate impact on the design of future interventions to change behaviours. Understanding how and why people in reality respond to the 'soft opt-out' will be vital to contextualising the impact of this Prudent health policy in achieving its aims. We want to explore the perspectives and decisions made by individuals who were involved in an organ donation decision, and to explore whether the donation decision reflected the patient or family view. There is also a potential benefit to participants as the study provides a confidential independent opportunity to talk about their views and experiences, which in turn can be used to benefit future donor families and patients.

Findings will fill a critical gap in knowledge to supplement the Welsh Government impact evaluation and shed light on the mechanisms that prevent or enhance organ and tissue donation under the new 'soft opt-out' system. Undertaking research to better understand these mechanisms and how they work will be vital for policy makers, healthcare professionals working in NHSBT and the NHS in general. It will inform continuous service improvement to realise the intended outcomes of this very complex intervention (the Act, media-based behaviour change interventions, retraining of NHS and NHSBT staff, and the interventions of NHS and NHSBT teams when requesting consent).

For peer review only - http://bmjopen.bmj.com/site/about/guidelines.xhtml

Aim

The aim of the study is to explore the impact of the Act on consent for deceased organ and tissue donation in the new ‘soft opt-out’ system. A secondary aim is to further build research capacity in NHSBT and Patient and Public Involvement (PPI) representatives in Wales.

Research questions

1. What impact and changes has the Act and media campaign had on the views and decisions of families of potential organ donors in Wales?
2. What were the views of the deceased person and how did families take account of the deceased person’s view in the decision-making process?
3. What are the views of families of the deceased person on the shift in relationship with the Government and healthcare services; organ donor registration; deemed consent; express patient decision and role of appointed representatives; and the changed role of families in decision-making in a ‘soft opt-out’ system?

Objectives

1. To ascertain a broad overview of retrospectively anonymised recorded family views, actions and outcomes from organ donation conversations in Wales for an 18 month period following implementation of the Act.
2. To explore in greater depth the perspectives and experiences of families who were involved in a donation conversation.
3. To explore the perspectives of SNODs and their managers covering Wales to contextualise potential donor family views, experiences and decisions.
4. To contextualise findings with publicly available quarterly activity reports on organ donor registration and organ donation in Wales.
5. To further develop research capacity and capability in NHSBT and patient and public representatives in Wales.

METHODS AND ANALYSIS

We consulted widely and extensively with multiple key stakeholders to design an ethically defensible and sensitive study that respects the vulnerability and confidentiality of bereaved potential donor families and the dignity of the deceased family member. The four phase design (Figure 7) combines use of routinely collected and publicly available potential donor audit activity as context to a primary study using shared anonymised and routinely collected NHSBT information on decision-making processes and outcomes of the donation consent process, and interviews with Welsh potential deceased donor families/appointed representatives/close friends and organ donation professionals

For peer review only - http://bmjopen.bmj.com/site/about/guidelines.xhtml

covering Wales.

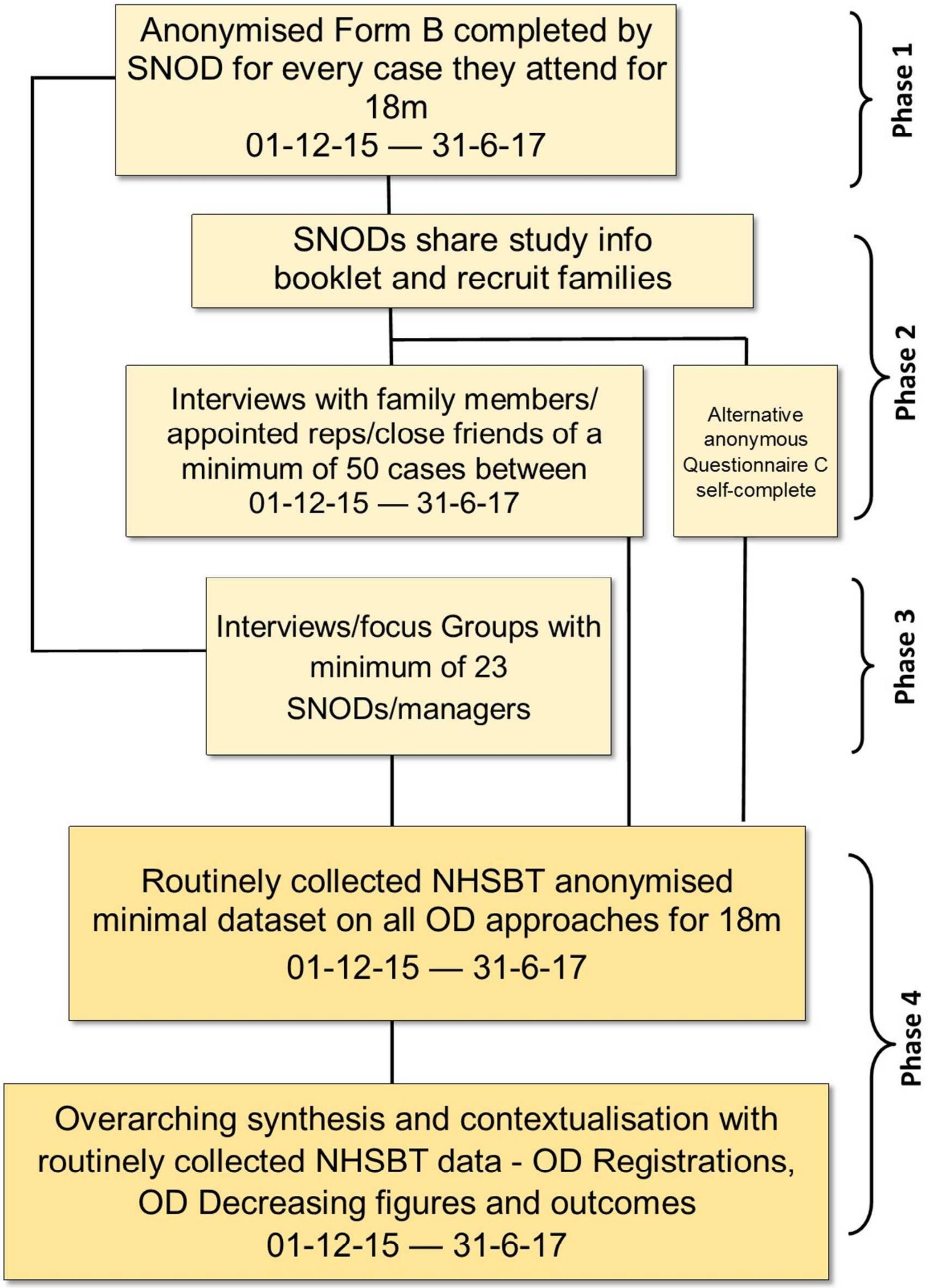

Figure 7. The four phase design. Key: NHSBT: NHS Blood and Transplant. OD: Organ Donation. SNOD(s): Specialist Nurse(s) in Organ Donation.

For peer review only - http://bmjopen.bmj.com/site/about/guidelines.xhtml

Phase 1 – Primary study to gain a high level understanding of the impact on donor family responses (accepting the patient's decision, or consenting to, or not consenting to donation) for 18 months from 1st December 2015.

SNODs will complete an anonymised electronic 1 page form [Appendix.1: Form B] that will be filled out as soon as possible after they have disengaged from speaking with the potential donor family. No participant or patient identifiable information will be recorded. SNODs will complete the form (electronic or paper) using information gathered from their routine conversations with potential donor families.

Phase 2: Primary study with potential deceased donor families/appointed representatives and close friends to ascertain a deeper understanding of their thoughts, experiences and responses to the Act and their decision-making.

Family members/appointed representatives and close friends, directly or indirectly involved in the donation process, will be invited to self-complete an anonymised questionnaire [Appendix 2: Form C] that requires no contact with the research team. There is no restriction on the number of questionnaires per family and the questionnaire will take around 20 minutes to complete. Accompanying the questionnaire will be an invitation to participate in an interview to discuss their views and experiences in greater depth and a contact form to send back to the independent research team to arrange a mutually convenient interview. For those wanting also to participate in an interview, several options will be offered that best suit the individual, such as face to face, telephone or via social media. Mindful that participants have been bereaved, they can select the time that is right for them to be interviewed up until the end of the period of data collection.

Family/close friend/appointed representative recruitment

We will use a range of methods to recruit participants that are sensitive and individually tailored. SNODs will use their discretion and knowledge of the family to select the most appropriate options and times to share information about the study with families/appointed representatives. Recruitment options include via direct contact with families by SNODs (with the option to using consent to contact form), and sharing study information in person; and by sending out a study invitation with information attached to routine follow-up communication by NHSBT; by direct mailing of study invitation and information by NHSBT; via adverts in the media, and through snowball sampling.

If an individual receives more than one letter of invitation we will include a sentence to explain that, if they have already made their decision whether to participate in the study or not, they can ignore

For peer review only - http://bmjopen.bmj.com/site/about/guidelines.xhtml

the letter or pass the invitation onto another family member or close friend of the deceased person, because NHSBT only have one contact name for each family. For participants who would prefer to be interviewed in their first language (Welsh or other language) we have employed a Welsh medium research officer and have built in interpreter costs.

Phase 3: Primary Study with qualitative 1:1 or small or focus group interviews with NHSBT organ donation teams covering Wales (SNODs and managers in our co-productive project) to contextualise potential donor family decision making, reactions and responses to the Act.

SNODs and their managers will be invited by letter with accompanying study information to participate in 1:1 and small or focus group interviews at the end of the study to contextualise the findings. Interviews will be at the end of the study and last approximately 60 minutes.

Inclusion criteria for 'family' participant recruitment

- Any person over 16 years with mental capacity who was involved, either directly or indirectly, in a deceased organ donation conversation or decision in Wales after 1st December 2015.
- Any person over 16 years with mental capacity who was involved, either directly or indirectly, in a deceased organ donation conversation or decision of a Welsh resident who died in a hospital in England after 1st December 2015 and was managed by the NHBT organ donation teams covering North or South Wales.
- Any close friends of the deceased person who may want to share their perspectives on the donation process and outcome
- Any NHSBT SNODs and managers covering Wales.

Exclusion criteria for participant recruitment

- Under 16 years
- Lacking mental capacity
- Welsh resident over 18 years who died in an English hospital not covered by participating SNODs

Sample

Recruitment targets, data capture targets and anticipated samples for each phase are shown in Figure 7.

Phase 4: Comparative analysis and overarching synthesis of stages 1-3.

Study data will be analysed and findings contextualised with data from NHSBT routinely collected audit reports and organ donor registration (opt-in or opt-out). These reports are publicly available

For peer review only - http://bmjopen.bmj.com/site/about/guidelines.xhtml

on the NHSBT website. Anonymised reports pending publication will be obtained directly from NHSBT.

Data Analysis

The Framework approach for analysis of applied policy research will be used for narrative textual data (questionnaires and interviews).[20] The Framework approach will be developed for each separate stream of data (questionnaires, interviews) and to accommodate the separate analysis of Welsh and English data, and then brought together in an overall analysis. First, the initial 5 available verbatim free text in questionnaires and transcripts will be read and reread and key themes and categories will be identified. The definitions and boundaries of each of the emerging themes for each type of evidence (questionnaires and transcripts) will then be discussed to see how these can be developed to form an a priori framework tailored for either questionnaires or transcripts in Welsh and English. Searching for additional themes will continue until all questionnaires and transcripts have been analysed and no new themes are discerned. Following analysis of Welsh language text, themes and relevant quotes will then be translated into English and back translated.

The final themes and their dimensions for questionnaires and interviews will then be further refined and used as the basis of charts (or matrices), which allows for themes to be compared and displayed for questionnaires and interviews, and for variations and deviant cases to be highlighted within each dataset. These charts will be overlaid with key information to preserve the original context. Secondly, these charts will undergo several revisions and further refinements, in an iterative process moving between the charts and the themes identified from questionnaires and interviews, until it is possible to synthesise the key findings across the datasets in a set of overall themes or categories. This stage will involve what is sometimes called the translation of themes from one data source to another. In the process of comparing the themes, we will look for explicit differences in relation to a range of factors that impact on decision-making including gender, relationship of the person to the deceased, age, ethnicity, and whether consent for donation was given or not and whether registration as an organ donor was viewed positively or negatively.

Anonymised record of 'approach conversation'

Completed form Bs will contain some structured options (such as the Yes, No, Uncertain), which will be collated in SPSS version 22 and analysed using descriptive statistics.[20] Narrative statements will be extracted and subject to thematic analysis using the Framework approach for applied policy analysis.[21]

For peer review only - http://bmjopen.bmj.com/site/about/guidelines.xhtml

For peer review only

Bilingual questionnaires

Data from questionnaire C will be entered into SPSS version 22 [20] and analysed using descriptive statistics. Open ended questions containing narrative text will be extracted in Welsh and analysed in English. Original and translated participant quotes to illustrate themes will be used to illustrate findings.[21]

Bilingual Interviews

With consent, interviews will be digitally recorded, transcribed verbatim and translated from Welsh to English.

Comparative analysis and overarching synthesis

We will use Oliver's approach for juxtaposing evidence across phases 1-3 with publicly available activity reports on donor activity and donor register numbers during the 2 year course of the study.[22] This will involve juxtaposing evidence to look for patterns, explanations and disconfirming cases.

Ethics and dissemination

This protocol was approved October 2015 by NHSBT Research, Innovation and Technology Advisory Group (RINTAG). The study was approved by an NHS research ethics committee (IRAS number 190066; Rec Reference 15/WA/0414) and the NHSBT Research and Development Committee (NHSBT ID: AP-15-02).

The design and methods are informed by an ethical framework developed by UK-based researchers for undertaking research with family members who are approached about organ donation and draws on the experiences of researchers working with the bereaved.[23-31] Independent governance will be provided by a steering group.

A key component of the ethical nature of the study will be the professional development and training elements to support SNODs and research officers to conduct the study in a respectful and sensitive way. We will dovetail the bereavement support offered by researchers to participants with the bereavement services offered by intensive care units in NHS Health Boards where potential organ donors are cared for with their families, and information provided on bereavement support services shared by SNODs in NHSBT teams covering Wales. In addition, in any contact with participants we will share a bilingual information leaflet on CRUSE Bereavement Care Cymru, in case families would prefer to access free support and counselling outside of a NHS context. In appreciation of their support, research team members will plan a fundraising activity during the course of the study to make a donation to Cruse Bereavement Care Cymru.

For peer review only - http://bmjopen.bmj.com/site/about/guidelines.xhtml

Additional information concerning the specific ethical and data protection[32] issues, proposed strategies and data sharing agreement can be found in **Appendix 3.**

Patient and Public Involvement

Prior to commencement of the study, contextual baseline engagement with the public has consisted of six discussion groups and seven face to face interviews involving fifty-two participants. This contextual work was undertaken by the Welsh Government.[18] Each group was recruited to include a mix of people in terms of awareness of the NHS Organ Donor Register and included some who had joined the Register and/or carried a donor card. Black and Minority Ethnic people formed part of the sample and included Pakistani, African Caribbean, Nigerian and Chinese participants. Each group contained a mix of men and women and the sample was broadly stratified by age and socioeconomic grouping. Two groups were conducted in the Welsh language. In addition, 1006 members of the public responded to a baseline Welsh Omnibus attitudinal survey.[7] Patient and public involvement representatives were involved in prioritising the question and in deciding to fund the study. The leading charities for supporting deceased organ donor families and people with kidney failure requiring a transplant have helped shape the design and advised on appropriate methods of data collection.

The Welsh Government hosted a conference in September 2015 involving those affected by deceased organ donation and health care professionals involved in the donation process to explore the implementation and implications of the Act from different perspectives and to explore how best to evaluate the Act and what outcomes from different perspectives are important. These perspectives have been incorporated into the study design. PPI will continue during the study through to dissemination.[33-4]

Building research capacity

A secondary aim of the study is to increase the confidence and capacity of NHSBT and PPI representatives to collaborate in future studies in this field.

In following Prudent healthcare principles,[13-14, 34] we will use a co-production approach, which means that the research team will work as equal partners and in collaboration with NHSBT who have a remit to support relevant research activity, and with a range of key professional stakeholders and PPI representatives to conduct the study. The co-production element is critical to the success of the study and will involve a strong research training and capacity building component for NHSBT teams and PPI representatives working in Wales. We have worked closely with policy and clinical leads from Welsh Government and NHSBT to ensure that the proposed co-productive methods of

For peer review only - http://bmjopen.bmj.com/site/about/guidelines.xhtml

data collection and participant recruitment are feasible, sensitive to the needs of potential donor families, and NHS staff, and fulfil the high ethical and data protection requirements for data sharing between two organisations.

Three development opportunities will bring NHSBT staff and PPI representatives together. At the beginning of the study we will facilitate professional development meetings with SNODs and managers to design the data collection tools. At the end of the first year of data collection, we will present initial findings at a professional development meeting with collaborating staff from NHSBT, clinical co-applicants, policy makers and PPI representatives to see what shared learning could be used to further enhance practice development and support study data collection. We will facilitate another meeting at the end of the study to present key findings.

Expected Outcomes

The most important outcome will be a research-informed and clearer, shared understanding of deceased donor consent decisions, and in particular the reasons why people continue to refuse to support consent in a 'soft opt-out' system, to feed back into further policy and practice development. In addition, staff in NHSBT covering Wales and PPI representatives will have developed additional confidence and research capacity and capability to undertake further and equally challenging studies.

Impact and Dissemination

The study has potential for high impact as success of this Prudent health policy is dependent on the people of Wales engaging with the principles of deemed consent and donor registration and honouring the deceased person's donation decision. If sufficient people agree and change their behaviour to favour the principles of the 'soft opt-out', then the policy will likely realise the anticipated benefits for patients. If sufficient people disagree then nothing will change and the anticipated increased number of patients who benefit from cost-effective transplants will not be realised.

Understanding why people do not register on the organ donor register or why family/appointed representatives still contest the decision to donate made by the deceased person will have an impact on the design of future interventions to improve organ and tissue donation rates in Wales.

The main mechanism of dissemination, knowledge transfer and maximising impact is through the uptake of project outputs by policy makers, clinicians and the public through a co-productive continuous quality improvement approach in line with Prudent healthcare principles.[13-14] There are

For peer review only - http://bmjopen.bmj.com/site/about/guidelines.xhtml

key elements known to affect the resources required for managing a successful co-productive dissemination processes and these elements are built into the project design, including:

• motivating change: creating readiness for change and overcoming resistance;

• creating a vision: mission, valued outcomes and conditions, midpoint goals and feedback;

• developing political support: assessing change agent power, identifying key stakeholders, influencing stakeholders;

• feeding back findings and jointly determining their meaning for various stakeholders;

• sustaining momentum: providing resources for professional development and research capacity building, building support systems for change agents, developing new competencies and skills, reinforcing new behaviours.[35]

Other effective elements of knowledge transfer include publication of research results in leading journals, and presentations at local, national and international conferences in the field. The research team has already demonstrated a high quality publication record, and will continue to do so, adopting an open access policy. We will also produce bilingual lay summaries.

REFERENCES

1. Human Transplantation Act (2013) Wales. http://www.legislation.gov.uk/anaw/2013/5/contents/enacted (Accessed 11th April 2017).
2. Welsh Government. Taking Organ Transplantation to 2020 – Wales Action Plan A plan up to 2020 for NHS Wales and its Partners Ensuring every organ donation counts. http://gov.wales/docs/dhss/publications/140107organactionen.pdf (Accessed 11th April 2017).
3. NHSBT. Taking Organ Transplantation to 2020. http://www.nhsbt.nhs.uk/to2020/resources/nhsbt_organ_donor_strategy_long.pdf. (Accessed 11th April 2017).
4. Personal Communication Karen Morgan, Major Health Conditions Policy Team, Directorate of Health Policy, Health and Social Services Group, Welsh Government. (April 2017).
5. Palmer M. (2012) Opt-out systems of organ donation: International evidence review. Social research Number: 44/2012 Welsh Government, Cardiff.
6. Welsh Government. OPTIONS FOR CHANGES TO THE ORGAN DONATION SYSTEM IN WALES CONSULTATION PAPER. 2009 http://www.wales.nhs.uk/sites3/Documents/773/Organ%20Donation%20consultation%201doc%20-%20English.pdf. Accessed 11th April 2017.
7. Welsh Government. Public Attitudes to Organ Donation: Baseline Survey 2012 Social research Number: 38/2012 Knowledge and Analytical Services Welsh Government Welsh Government, Cardiff. http://gov.wales/docs/caecd/research/121019organdonationen.pdf (Accessed 11th April 2017)
8. Palmer M. (2012) The role of families in organ donation: International evidence review. Social research Number: 45/2012, Welsh Government, Cardiff.
9. Human Tissue Act 2004. https://www.hta.gov.uk/policies/human-tissue-act-2004. (Accessed 11th April 2017).
10. Walker W. Broderick A. and Sque M. (2013) Factors influencing bereaved families' decisions about organ donation: An integrative review of the literature. Western Journal of Nursing Research,Doi.10.1177/0193913484987. 35: 10, 1339-1359.
11. MRC Framework for developing and evaluating complex interventions: New Guidance 2006. https://www.mrc.ac.uk/documents/pdf/complex-interventions-guidance/. (Accessed 11th April 2017)

For peer review only - http://bmjopen.bmj.com/site/about/guidelines.xhtml

12. Local Government Association. Changing behaviours in public health To nudge or to shove? Local Government Association. 2013. http://local.gov.uk/sites/default/files/documents/changing-behaviours-publi-e0a.pdf. (Accessed 11th April 2017)
13. Mark Drakeford, Minister for Health and Social Services (2014) Written Statement – Delivering Prudent Healthcare in Wales. http://gov.wales/about/cabinet/cabinetstatements/2014/Prudenthealthcare/?lang=en. (Accessed 11th April 2017)
14. Aylward M, Phillips C, Howson H. (2013) Simply Prudent Healthcare – achieving better care and value for money in Wales – discussion paper. Bevan Commission Simply Prudent Healthcare 4 December 2013.v1. http://www.ctrtraining.co.uk/documents/BevanCommissionSimplyPrudentHealthcarev104122013.pdf. (Accessed 11th April 2017)
15. Organ Donation Wales website. http://organdonationwales.org/FAQs/Organ-donation-from-december-2015/?skip=1&lang=en (Accessed 11th April 2017)
16. McAllister F & Blunt A. Research to support Wales' organ donation opt-out proposal consultation. Qualitative research report Social research Number: 14/2012. Welsh Assembly Government. http://gov.wales/docs/caecd/research/120418organdonationen.pdf. (Accessed 11th April 2017).
17. GfK NOP. Evaluation of the Human Transplantation (Wales) Act: Baseline Survey of NHS Wales Staff. Social research Number: 68/2014 Welsh Assembly Government. http://gov.wales/docs/caecd/research/2014/140707-evaluation-human-transplantation-wales-act-survey-nhs-wales-staff-en.pdf. (Accessed 11th April 2017)
18. Choudhery R. Evaluation of the Human Transplantation (Wales) Act: Qualitative Study of Public Attitudes 87/2014 Welsh Assembly Government. http://gov.wales/docs/caecd/research/2014/141007-human-transplantation-act-qualitative-study-public-attitudes-en.pdf. (Accessed 11th April 2017)
19. NHS Blood and Transfusion (2014) Cost-Effectiveness of Transplantation. http://www.organdonation.nhs.uk/newsroom/fact_sheets/cost_effectiveness_of_transplantation.asp (Accessed 11th April 2017)
20. IBM SPSS Statistics V22.0, https://ibm-spss-statistics-base.en.uptodown.com/windows. (Accessed 11th April 2017)
21. Ritchie J. & Spencer L. (1994) Qualitative data analysis for applied policy research. In: Analysing Qualitative Data (Bryman A. & Burges R. eds.), pp. 173-194.
22. Oliver S, Harden A, Rees R: (2005) An emerging framework for including differenttypes of evidence in systematic reviews for public policy. Evaluation 2005, 11(4):428–446.
23. Jansen N.E. McDonald M. Haase-Kromwijk BJJM. Sque M. and Long-Sutehall T. (2014) When are bereaved family members approached for consent to organ donation? Commentary from 10 European member states. Organs, Tissues and Cells, 17, 101-113.
24. Sque M. Walker W. and Long-Sutehall T. (2014) Research with bereaved families: A framework for ethical decision-making. Nursing Ethics doi:10.1177/0969733014521097.
25. Sque M, Long T, Payne S. (2003) Organ and tissue donation: Exploring the needs of families Summary of the Final. Sponsored by the British Organ Donor Society Funded by the National Lottery Community Fund February 2003. https://eprints.soton.ac.uk/191475/1/BODY_study.pdf (Accessed 11th April 2017)
26. Sque M, Long T, Payne S, Allardyce D. Why relatives do not donate organs for transplants: 'sacrifice' or 'gift of life'? J Adv Nurs. 2008 Jan;61(2):134-44. doi: 10.1111/j.1365-2648.2007.04491.x.
27. Sque M. and Galasinski D. (2013) 'Keeping her whole': bereaved families' accounts of declining a request for organ donation. Cambridge Quarterly of Healthcare Ethics, Special Edition, 22: 1, 53-63.
28. Jansen NE. Haase-Kromwijk BJJM. McDonald M. and Sque M. (2013) Elements of the European jigsaw: the timing of the approach to bereaved families for consent to DBD or DCD donation and the implications for effective donation campaigns. In Randhawa G. and Schicktanz S. (Eds.) Public engagement in organ donation and transplantation. Lengerich, Germany: PABST Science Publishers, 161-169.
29. Long-Sutehall T. Winstanley E. Clarkson A. and Sque M. (2012) Evaluation of the experiences of family members whose deceased relative donated tissues at the NHSBT dedicated donation facility in Speke, Liverpool. Cells and Tissue Banking, 13: 4, 537-546.
30. Sque M. and Long T. (2011) Bereavement, decision-making and the family in organ donation. In Farrell AM. Price D. and Quigley M. (Eds.) Organ shortage: principles, pragmatism and practice, Cambridge: Cambridge University Press, 67-85.
31. Sque M. and Payne. S. (Eds.) (2007) Organ and tissue donation: an evidence base for practice. Maidenhead: Open University. Australian Journal of Medical Science 29: 1, 20.

For peer review only - http://bmjopen.bmj.com/site/about/guidelines.xhtml

32. The Data Protection Act 1998. Reviewed March 2017 http://www.legislation.gov.uk/ukpga/1998/29/contents (Accessed 11th April 2017)
33. NIHR Involve (2014) Policy on payment of fees and expenses for members of the public actively involved with INVOLVE August 2014 http://www.invo.org.uk/wp-content/uploads/2014/11/INVOLVE-internal-payment-policy-FINAL-August2014.pdf. (Accessed 11th April 2017)
34. Jung T, Harrow J and Pharoah C (2012) Co-producing research: working together or falling apart? CGAP Briefing Note 8.http://www.cgap.org.uk/uploads/Briefing%20Papers/CGAP%20Briefing%20Note%208%20-%20Co-producing%20research.pdf. (Accessed 11th April 2017)
35. French, W. and Bell, C. (1999) Organization Development. New Jersey, USA: Prentice Hall.

List of Figures

Figure 1. Nuffield Council of Bioethics ladder of intervention.
Figure 2. Intervention implementation: Multi-facetted media-based strategy to inform the public of the Act and changes to consenting to organ donation.
Figure 3. Intervention and required behaviours following introduction of the Act
Figure 4. Intervention implementation: Additional training for Specialist Nurses in Organ Donation when approaching families following the implementation of deemed consent.
Figure 5. . Wales potential organ donor population and identification of the 'donation request' stage in the process.
Figure 6 Sequence of obtaining consent when the patient is NOT on the Organ Donor Register (ODR).
Figure 7. The four phase study design.

Supplementary Files: List of Appendices

Appendix 1.FORM B: Completed by Specialist Nurse in Organ donation**.**
Appendix 2.FORM C: Questionnaire completed by family members/close friends.
Appendix 3.Ethical issues and strategies

Author contributions

Jane Noyes – Chief Investigator conceptualised the idea, put the team together, designed the study and procedures, and drafted the protocol.

Michael Stephens - Consultant Transplant and Organ Retrieval Surgeon, Clinical Lead for Transplantation, Cardiff and Vale Health Board - advised on key research team members and stakeholders to bring into the research team, proposed changes in the law and key research questions to address.

Karen Morgan – Formerly Regional Manager South Wales and South West, NHSBT and now Major Health Conditions Policy Team, Directorate of Health Policy, Health and Social Services Group, Welsh Government – advised on key changes to policy and practice, study design and processes, data collection tools and implementation of the study.

Phillip Walton – Regional Manager South Wales NHSBT advised on on changes to policy and practice, study design and processes, data collection tools, and implementation of the study.

Abigail Roberts – Specialist Nurse in Organ Donaiton NHSBT advised on the role of the Specialist Nurse in Organ Donation, study design and processes, data collection tools and implementation of the study.

For peer review only - http://bmjopen.bmj.com/site/about/guidelines.xhtml

Leah Mclaughlin – Research Officer – finalised study procedures and data collection processes, designed the study documentation and logos and supported production of applications to the NHS REC and NHSBT R&D committees.

All authors agreed the final manuscript.

Acknowledgments

Fiona Wellington – Head of Operations NHSBT for supporting the study.

Christian Brailsford – NHSBT provided advice and support to agree a mutual data sharing agreement and negotiate NHS ethics and NHSBT RINTAG and NHSBT R&D processes.

Pat Vernon (Policy Lead Welsh Government), Ian Jones (Research and Evaluation Lead) Caroline Lewis (Organ Donation Policy Manager) provided a Government perspective and shared research carried out prior to implementation of the Act.

Donald Fraser – Lead of the Wales Kidney Research Unit supported development of the funding application and serves as independent Chair of the steering group.

Jo Mitchell – research support officer

North West NHSBT Team: Ben Armstrong, Adam Barley Angela Campion-Sheen, Laura Ellis-Morgan, Rebecca Gallagher, Sharon Hallam, Phil Jones, Andrew Mawson, Abi Roberts, Tracey Rhodes, Helen Bullock, Andrea Jones, Kathryn Alletson, Jane Monks, Emma Thirlwall, Dawn Lee, Nicky Hargreaves, Lisa Welsh, Gill Drisma, Sue Duncalf.

South Wales NHSBT Team: Angharad Griffiths, Lucy Barnes, Charlotte Goodwin, Guy Heathcote, Gail Melvin, Michael Tobin, Lisa Morgan, Nicola Newbound, Michelle Powell Stephen Regan, Fiona Rogers, Susie Cambray, Kathy Rumbleow, Lynne Woolcocks, Janet Woodley, Beth Moss, Louise Colson.

NHSBT Staff: Sian Griffin (Consultant Nephrologist, Department of Nephrology and Transplantation) Katja Empson, Sam Sandow, Carl Stephenson (Clinical Leads Organ Donation) Francesca Stevens (Tissue Services NHSBT), Maggie Stratton (PR Officer NHSBT).

Jeanette & CRUSE Bereavement Care – who provided leaflets signposting bereavement support for participants.

Patient and Public representatives: Sarah Thomas, Janet Thickpenny, Gethin Rhys, Michael Rhys, Maria Mesa, Roon Adams, Michael Houlston, Maria Battle, Anna Bates for providing guidance and advice on the focus of the study.

Janet Williams and Gloria Owens for providing advice and feedback on processes, participant facing documents and the detailed funding application.

Patient and Public representative organisations: Flintshire Deaf Association, Llanelli Multi Cultural Network, Big Lottery, Churches Together in Wales, Women Connect First, BAWSO, Race Equality First, Believe, Donor Family Network, Rita's Café for helping to set up the patient and public network and supported the study proposal.

For peer review only - http://bmjopen.bmj.com/site/about/guidelines.xhtml

Gareth Wyn Roberts - Consultant Nephrologist, Cardiff and Vale University Health Board provided detailed advice on clinical processes and the new Act.

Catherine Robinson – Former Head of School of Social Sciences, Bangor University, supported submission of the funding application following high level discussions and commented on a section of the application.

Funding statement

The study was funded by Health and Care Research Wales. Project Reference 1129.

Competing interests statement

There are no known competing interests.

For peer review only

For peer review only - http://bmjopen.bmj.com/site/about/guidelines.xhtml

FORM B Organ Donation Study: Questionnaire for SNOD to document outcome of all approach conversations

Record of conversation with (tick all that apply): spouse or partner ☐ **parent or child** ☐ **brother or sister** ☐ **grandparent or grandchild** ☐ **niece or nephew** ☐ **stepfather or stepmother** ☐ **half-brother or half-sister** ☐ **friend of long standing** ☐ **appointed representative** ☐ (referred to as 'family' hereafter)

Age range of deceased person: ☐ **0-18** ☐ **19-35** ☐ **36-50** ☐ **51-70** ☐ **>71**

What region in Wales did family members/friends come from (tick all that apply): ☐ Isle of Anglesey ☐ Conwy ☐ Gwynedd ☐ Denbighshire ☐ Flintshire ☐ Wrexham ☐ Powys ☐ Ceredigion ☐ Pembrokeshire ☐ Carmarthenshire ☐ Swansea ☐ Neath Port Talbot ☐ Bridgend ☐ Rhondda Cynon Taff ☐ Merthyr Tydfil ☐ Blaenau ☐ Torfaen ☐ Monmouthshire ☐ Caerphilly ☐ Newport ☐ Cardiff ☐ Vale of Glamorgan ☐ Outside of Wales

Clear Form

For peer review only

1. Was a SNOD involved in the donation conversation?
   ☐ Yes ☐ No

2. Did the patient die in hospital in:
   ☐ Wales (go to Q3b) ☐ England (go to Q3)

3. For Welsh patients that died in England what was the patient registered decision on ODR? ☐ Opt In ☐ Opt Out ☐ Appoint Representative ☐ Not Registered

   Would the patient have fitted deemed consent criteria if they had died in Wales? ☐ Yes ☐ No

   Did the family think that deemed consent would apply?
   ☐ Yes ☐ No ☐ Uncertain ☐ Non Applicable (N/A)

   Was consent obtained ? ☐ Yes ☐ No ☐ N/A

   Had the family heard or read about the Act from the media campaign (Radio, TV, newspapers, postal leaflet etc)?
   ☐ Yes ☐ No ☐ Uncertain ☐ N/A

   Were the family positive about the new Act?
   ☐ Yes ☐ No ☐ Uncertain ☐ N/A (go to Q.19)

3b. For Welsh patients who died in Wales, what was their registered decision on the ODR? ☐ Opt In ☐ Opt Out
   Appoint Representative ☐ Not Registered ☐

4. If patient was under 18 was their decision recorded on ODR?
   ☐ Yes ☐ No ☐ Non Applicable (N/A)

5. For patients of any age, did the family know of any other expressed decision?
   ☐ Yes ☐ No ☐ Uncertain ☐ N/A

6. Did the family agree with the patient's expressed donation decision? ☐ Yes ☐ No ☐ Uncertain ☐ N/A

7. Was there disagreement between one or more family members about the patient's expressed decision?
   ☐ Yes ☐ No ☐ Uncertain ☐ N/A

8. Was the patient's registered or expressed donation decision supported? ☐ Yes ☐ No ☐ N/A

9. Only for patients who appointed a representative (AR).

   Did the AR give consent? ☐ *Yes* ☐ *No*

   Did the AR support new Act? ☐ *Yes* ☐ *No* ☐ *Uncertain*

   Were the family aware of AR? ☐ *Yes* ☐ *No* ☐ *Uncertain*

   Did the family agree with the decision of the AR?
   ☐ *Yes* ☐ *No* ☐ *Uncertain* ☐ *N/A*

10. Did deemed consent apply? ☐ Yes ☐ No ☐ N/A

11. Did the family agree with the deemed consent of the patient?
   ☐ Yes ☐ No ☐ N/A
   If no, did you feel able to get the family to support the deemed consent? ☐ Yes ☐ No ☐ N/A

12. Was there disagreement within the family and close friends involved in the deemed consent conversation?
   ☐ Yes ☐ No ☐ Uncertain ☐ N/A
   If yes, did you feel able to get the group to support the deemed consent? ☐ Yes ☐ No ☐ N/A

13. Was consent obtained? ☐ Yes ☐ No ☐ N/A

14. Who consented? ☐ family member ☐ friend ☐ other ☐ N/A

15. Had the family heard or read about the Act from the media campaign (Radio, TV, newspapers, postal leaflet etc)?
   ☐ Yes ☐ No ☐ Uncertain ☐ N/A

16. Did the family have a correct understanding of the Act BEFORE you spoke to them? ☐ Yes ☐ No ☐ Uncertain ☐ N/A

17. Did the family have a correct understanding of the Act AFTER you spoke to them? ☐ Yes ☐ No ☐ Uncertain ☐ N/A

18. Had the new Act changed the family views of organ donation?
   ☐ Yes ☐ No ☐ Uncertain ☐ N/A
   If yes, were they positive about the new Act? ☐ Yes ☐ No

19. Did any person suggest that they would register their decision in light of your conversation?
   ☐ Yes ☐ No ☐ N/A
   If yes, would they: ☐ Opt in ☐ Opt out ☐ Appoint Representative ☐ Do nothing

20. Has this Act influenced your approach conversation with this family? ☐ Yes ☐ No ☐ Uncertain ☐ N/A

Anything else that could be helpful (e.g. Was there a pre-approach, a complaint or anything in this case that impacted upon you getting consent under the new law?)

Return by email to organdonationstudys@nhsbt.nhs.uk (for South Wales team) as soon as possible.

Submit

FORM C: Organ Donation: Questionnaire for Family/ Close Friends/ Appointed Representatives

Thank you for agreeing to fill out this questionnaire. Anybody involved in the donation decision can choose to fill out a questionnaire. The questions are quite short but feel free to add more details at the bottom. There is an option complete this online if you prefer: https://bangor.onlinesurveys.ac.uk/organ-donation-project-questionnaire. There is no limit to the number of questionnaires per family. By returning the completed questionnaire to the research team at Bangor University, it will be assumed that you have given your consent for the researchers to analyse and use the data.

1. Are you: spouse or partner ☐ parent or child ☐ brother or sister ☐ grandparent or grandchild ☐ niece or nephew ☐ stepfather or stepmother ☐ half-brother or half-sister ☐ friend of long standing ☐ Appointed Representative ☐

(*An appointed representative is a person appointed by your loved one or close friend during their lifetime to convey their organ donation decision after their death.)*

2. Did your loved one or close friend pass away in a hospital in:
Wales ☐ (go to Q.4) England ☐ (go to Q.3)

3. Did you know that the changes to consenting to organ donation in Wales did not apply in England? Yes ☐ No ☐ Uncertain ☐

4. On the organ donor register, did your loved one or close friend:
Opt In ☐ Opt Out ☐ Appoint a Representative ☐ Do Nothing ☐

5. Did you know about your loved one or close friend's decision on the organ donor register?
Yes ☐ No ☐ Uncertain ☐ Non Applicable ☐

6. Did your loved one or close friend ever discuss their donation decision with you?
Yes ☐ No ☐ Uncertain ☐ Non Applicable ☐

7. Did you support your loved one or close friend's organ donation decision after they passed away?
Yes ☐ No ☐ Uncertain ☐ Non Applicable ☐

8. Did you feel able to support the deemed consent of your loved one or close friend when the changes to consenting to organ donation in Wales were explained by a specialist nurse in organ donation?
Yes ☐ No ☐ Uncertain ☐ Non Applicable ☐

9. Do you consider yourself the decision maker on behalf of your loved one or friend who passed away? Yes ☐ No ☐ (go toQ.11)

10. As the decision maker, did the changes to consenting to organ donation in Wales help you at this difficult time?
Yes ☐ No ☐ Uncertain ☐

For peer review only - http://bmjopen.bmj.com/site/about/guidelines.xhtml

Please turn over the page now

11. Was there any form of disagreement between anybody involved in the donation discussion?
    Yes ☐ No ☐ Uncertain ☐ Non Applicable ☐
    If yes, did the changes to consenting to organ donation in Wales help to reach agreement?
    Yes ☐ No ☐ Uncertain ☐ Non Applicable ☐

12. When somebody sadly passes away and they meet very specific criteria in Wales, if they have NOT registered a decision on the organ donor register or have NOT discussed their organ donation decision with family and friends, it means that their consent to organ donation can be deemed.
    On a scale of 1 to 10 (1 = not at all and 10 = fully understood)

    How well would you say you understood the changes to consenting to organ donation in Wales BEFORE you spoke to a specialist nurse in organ donation?
    1 ☐ 2 ☐ 3 ☐ 4 ☐ 5 ☐ 6 ☐ 7 ☐ 8 ☐ 9 ☐ 10 ☐

    How well would you say you understood the changes to consenting to organ donation in Wales AFTER you spoke to a specialist nurse in organ donation?
    1 ☐ 2 ☐ 3 ☐ 4 ☐ 5 ☐ 6 ☐ 7 ☐ 8 ☐ 9 ☐ 10 ☐

13. Had you heard or read about the changes to consenting to organ donation in Wales from the media campaign (Radio, TV, newspapers, postal leaflet etc.)?
    Yes ☐ No ☐ Uncertain ☐

14. Have the changes to consenting to organ donation encouraged you to register your decision on the organ donation register?
    Yes ☐ No ☐ Uncertain ☐ Non Applicable ☐

Other comments Please tell us anything else that you think is important.

____
____
____
____
____
____
____
____
____
____
____
____
____

Thank you kindly for your responses at this difficult time. If you would like to share your stories with us in more detail, please fill out Form D (consent to be contacted for interview) and return with this questionnaire in the pre-paid envelope. We look forward to hearing from you.

For peer review only

Appendix 3. Ethical issues and strategies

Undertaking research with vulnerable participants: Grieving families, close friends and appointed representatives are vulnerable and considered to be most affected by the Act. It appears appropriate, with the right support and safeguards in place, to provide an opportunity for them to participate in a study to elicit their perspectives. Our patient representatives have written a letter of support indicating that families will want the option to be able to talk to someone outside of the NHSBT Team, to share their positive or negative views about how their role has changed under the new Act and whether they agreed with the decision of the deceased person or not.

Although they have been recently bereaved, potential donor families, close friends and appointed representatives may want an opportunity and may benefit from expressing their views, which in turn may inform development of practice in a continuous improvement cycle. To ensure that an appropriate approach is made to potential participants at an appropriate time, we will ask the Specialist Nurses in Organ Donation (SNODs) to use their judgement as to the most appropriate time to share information on the study. SNODs will have spent a lot of time with the people involved in the donation conversation and can use their professional judgement to select from a range of methods to recruit participants that are individually tailored for each situation, including: via direct contact with families by SNODs; by sending study invitation and information attached to routine follow up communication by SNODs; by direct mailing of study invitation and information by NHS Blood and Transplant. In addition we will place three adverts in the national media at staged intervals and recruit through snowball sampling. If a family member, close friend or appointed representative knows of another person involved in the organ donation process who may want to be interviewed, we will ask them to share a letter of invitation and study information with them and ask the person to contact us directly.

We will follow the sensitive ethical framework[2] and practical strategies shown in Table 1. Participants who return self complete anonymised questionnaires and who wish to themselves remain anonymous will be made aware that returning a completed questionnaire to the research team will constitute consent to use the data. For all other research procedures such as interviews informed written consent will be required. All participants will be over 16 years with mental capacity to consent. Participants can choose to be interviewed at any point that is appropriate and convenient for them after their bereavement up until the end point of data collection. If an individual receives more than one letter of invitation we will include a sentence to explain that, if they have already made their decision whether to participate in the study or not, to ignore the letter or to pass the invitation on to another family member or close friend of the deceased person, because NHSBT only have one contact name for each case. Researchers will follow a ‘Distress Protocol’ (see below) when conducting interviews and if participants become unduly distressed they will use the three step approach in the protocol to safeguard the wellbeing of participants.

CRUSE Bereavement Care Cymru aim to reach out and support all bereaved people in Wales. We will share their client information with participants and the contact numbers for CRUSE bereavement care and support. If the research officer has any serious concerns

For peer review only

about the safety of any participant in the study, they will follow the standard protocol of NHSBT for Safeguarding Vulnerable People.

Anonymity of Professionals: Interviews with professionals will be conducted at a venue of their choice, and they will be assigned a code. Where there is only one role in the organisation care will be taken not to identify participants by using their quotes without permission. Participants will be offered a choice of 1:1 or small group or focus group interviews if they do not wish their experiences to be shared with colleagues.

Support for researchers undertaking sensitive interviews: The research team is large (5 core members) to provide a mutually supportive context and debriefing will be offered after interviews. Researchers have been recruited with skills and experience of undertaking interviews on sensitive topics with vulnerable people. The research team is also working in partnership with NHSBT teams who provide an additional supportive environment and peer to peer support and mentorship concerning sensitive issues, should research team members need it. We have built in three joint professional development opportunities for support, shared learning and reflection. Researchers can also contact CRUSE bereavement care for additional confidential and independent support.

Good clinical practice. Core research team members have undertaken Good Clinical Practice in research training and those who will have contact with family members have been subject to screening under the Disclosure and Barring Service.

Data Protection and Data Sharing Agreement

The research requires anonymised data to be collected and shared between NHSBT and Bangor University. Bangor University has entered into a standard data sharing agreement with NHSBT. Data collected directly by the research team will be recorded on encrypted digital recorders, regularly downloaded, assigned a unique code and transcribed, anonymised and stored securely on password protected university servers and laptops. Data on digital recorders will then be deleted. Any paper based patient identifiable information will be stored under lock in a secure place with access controlled by the research team.

The following process has been previously adopted in other studies to share non-patient identifiable information. The process is designed to enable the research team at Bangor University to have access to anonymised information recorded by SNODs that sheds light on decision-making by families/appointed representative(s) (especially when they say no to organ donation). Patient and Public representatives from partner non-government organisations who support bereaved families have been consulted and support this approach. The structured communication process used by SNODs during the donation request stage will be amended for introduction of the new Act and documented in NHSBT Standard Operating Procedures and Management Process Descriptions. The 'approach' conversation is highly structured and follows a ladder of issues for families to think about, consider and discuss. The SNOD is trained to explore and clarify any issues or questions that are raised and to facilitate and listen to reasons and concerns when considering giving or declining consent to donation. To capture this information in a structured and anonymised way that falls outside of the Data Protection Act, we have agreed a plan with the two NHSBT donor teams covering Wales, to jointly develop a standard proforma for SNODs to record

For peer review only

key anonymised information from the 'approach' conversation for research purposes as soon as practical after it has concluded and once they have disengaged from the family. The anonymised information recorded on the proforma will be returned electronically to the researchers in batches so that it is not possible to link any single proforma with any specific death. We will also be respectful of professional anonymity and the proformas will be designed in such a way that the identity of the hospital or person who completed it will not be known to researchers.

Table 1. Study framework for ethical decision-making.[2]

Ethical considerations	Practical strategies
	**Participant identification and recruitment**
Access, confidentiality Regard	Formally obtain the support of a key person to undertake the role of identifying potential participants and disseminating pre-prepared recruitment packs on behalf of the research team.<br>Recruit potential participants in a serial manner, for example, send out a maximum of five recruitment packs at any one time so that participants are not kept waiting for long periods before the research interview.
Respect, relevance	Consider participant inclusion criteria of bereaved no less than 3 months and no more than 12 months at the time of recruitment to the study. * *We will ask Specialist Nurses in Organ Donation to share study information at the time of bereavement and will offer the option of a self-complete questionnaire that involves no face to face contact with researchers and an interview at a time when the participant feels that it is appropriate for them. We have built in a 3 month time lag to collect data after the last participant has been contacted.*
Compassion	Include a covering letter that introduces the study in a personalised way by taking familiarity into consideration.
Informed choice	Provide clear written and web-based information about the researchers and the study.<br>Include an invitation to contact the researcher.<br>Demonstrate timely responsiveness to any potential questions or queries.
Non-coercion	Provide a minimum of 10 days for participants to decide about joining the study.
	**The research interview**
Choice, respect	Agree a convenient date, time and venue for the research interview. Avoid dates that coincide with any significant family events or anniversaries.
Safety	Implement a study site policy for researchers working alone in advance of the interview encounter.
Safety, support	Competent researcher with experience of conducting sensitive research interviews and supporting the bereaved.
Choice, privacy	Provide the option of an interview face to face or remotely, for example, via telephone.
Informed consent	Provide an overview of the study and present opportunity for participants to ask questions. Explain how the interview will proceed. Obtain written agreement to audio-record the interview and to use anonymous quotes in any presentation of the research. Provide participants with a copy of the signed consent form to keep.
Support	Discuss and agree avenues of post-interview support prior to the interview commencing<br>Observe/listen for signs of distress during the interview.<br>Discuss the option of pausing the recording or stopping the interview. Plan a natural break for refreshments.
Confidentiality, anonymity	Ensure audio-recordings and transcripts are securely stored and electronic data are password protected. Assign a study code at the point of transcription.
	**Post-interview follow-up care**

For peer review only

Support	Arrange a convenient time to telephone the participant (normally in 24–48 h) to check on any issues the interview may have raised and to answer any questions.<br>Compile information about local support organisations. Offer this to participants if they consider it helpful and/or direct them to appropriate professionals to discuss any issues of concern.<br>Establish if participants wish their general practitioner (GP) to be informed about their participation in the study and obtain written consent to proceed. Provide GP with information about the study at the time of notification.
Appreciation	Send participants a personal thank-you letter and offer an executive summary of the research findings.
Involvement	Provide participants with an opportunity to evaluate their experience of participating in bereavement research.
Researcher Support	Determine support for the researcher from an individual with whom they feel comfortable and who is suitably qualified to provide support. Plan a debriefing session after each interview encounter. Utilise reflexive notes to guide the discussion.

Distress Protocol

During instances of bereaved participants becoming distressed during the interview process, the subsequent protocol will be followed.

Identifying Distress

The interviewer will be mindful of signs of distress in the participants throughout the interviews. Signs of distress to look out for will include:

- Exhibition of behaviours that indicate that the discussion has become too upsetting for them, including crying and an inability to continue for example.
- The participant verbally communicating that they are experiencing distress during the interview.

Response Stage 1

- The interview will be stopped.
- The participant will be offered a break, have a drink of water/ tea etc.
- The participant will then be asked if they would like to continue the interview or if they would prefer to discontinue. Should they wish to go on, the interview will resume.

For peer review only

Response Stage 2

If the participant elects to discontinue the interview,

- The interview will not continue.
- The interviewer will signpost where support can be obtained such as from the SNOD or the bereavement support service at the hospital where their relative/friend died, or from their GP.
- The participants will also be reminded again of the contact details for Cruse Bereavement Care,' an organisation which provides support for bereaved people.

Response Stage 3

- At a later date, the interviewer will follow up with the participant with a courtesy call (with the participant's consent). If the participant feels strongly that they would still like to have their views and experiences heard – the interviewer will go through options (wait a while before rearranging, explore other methods rather than face to face etc.

References

1. The Data Protection Act 1998. Reviewed March 2017 http://www.legislation.gov.uk/ukpga/1998/29/contents (Accessed March 2017).
2. Sque M. Walker W. and Long-Sutehall T. (2014) Research with bereaved families: A framework for ethical decision-making. Nursing Ethics doi:10.1177/0969733014521097.

For peer review only

Family attitudes, actions, decisions and experiences following implementation of deemed consent and the Human Transplantation (Wales) Act 2013: Mixed-method study protocol

Journal:	*BMJ Open*
Manuscript ID	bmjopen-2017-017287.R1
Article Type:	Protocol
Date Submitted by the Author:	11-Jul-2017
Complete List of Authors:	Noyes, Jane; School of Social Sciences<br>Morgan, Karen ; Welsh Government , Major Health Conditions Policy Team, Directorate of Health Policy, Health and Social Services Group, Welsh Government, Cardiff, Wales, UK.<br>Walton, Philip; NHS Blood and Transplant , Organ Donation Department, Unit 3 Cae Gwyrdd, Greenmeadow Springs Business Park, Tongwynlais, Cardiff, UK.<br>Roberts, Abigail; NHS Blood and Transplant<br>Mc Laughlin, Leah; School of Social Sciences<br>Stephens, Micheal; Cardiff and Vale University Health Board, University Hospital of Wales, Department of Nephrology and Transplantation, Heath Park, Cardiff, UK.
**Primary Subject Heading**:	Health services research
Secondary Subject Heading:	Research methods, Surgery, Nursing
Keywords:	Change management < HEALTH SERVICES ADMINISTRATION & MANAGEMENT, Health policy < HEALTH SERVICES ADMINISTRATION & MANAGEMENT, Organisational development < HEALTH SERVICES ADMINISTRATION & MANAGEMENT, Transplant medicine < INTERNAL MEDICINE, LAW (see Medical Law), Renal transplantation < NEPHROLOGY

SCHOLARONE™
Manuscripts

TITLE PAGE

Family attitudes, actions, decisions and experiences following implementation of deemed consent and the Human Transplantation (Wales) Act 2013: Mixed-method study protocol

Corresponding Author: Jane Noyes,
Address: Room 107, Neuadd Ogwen, School of Social Sciences, Bangor University, Wales, LL57 2DG, UK
Email: jane.noyes@bangor.ac.uk
Phone:+44 (0) 1248 388519

Authors
Jane Noyes[1]
Karen Morgan[2]
Phillip Walton[3]
Abigail Roberts[4]
Leah Mclaughlin [1]
Michael Stephens[5]

1. School of Social Sciences, Bangor University, Bangor, Wales, UK.
2. Major Health Conditions Policy Team, Directorate of Health Policy, Health and Social Services Group, Welsh Government, Cardiff, Wales, UK.
3. NHS Blood and Transplant, Organ Donation Department, Unit 3 Cae Gwyrdd, Greenmeadow Springs Business Park, Tongwynlais, Cardiff, UK.
4. NHS Blood and Transplant, North West Regional Office, 14 Estuary Banks, Speke, Liverpool, UK.
5. Cardiff and Vale University Health Board, University Hospital of Wales, Department of Nephrology and Transplantation, Heath Park, Cardiff, UK.

For peer review only

Abstract

Introduction

The Human Transplantation (Wales) Act 2013 (the Act) introduced a ‘soft opt-out’ system of organ donation on 1/12/15. Citizens are encouraged to make their organ donation decision known during their lifetime. In order to work, the Act and media campaign need to create a context whereby organ donation becomes the norm, and create a mechanism for people to behave as intended (formally register their decision; consider appointing a representative; convey their donation decision to their families and friends, or do nothing – deemed consent). In addition, family members/appointed representatives need to be able to put their own views aside to support the decision of their loved one. The aim of this study is to evaluate initial implementation, outcomes, and impact on families and appointed representatives who were approached about organ donation during the first 18 months.

Methods and analysis

Prospective mixed-method co-productive study undertaken with National Health Service Blood and Transplant (NHSBT), and multiple patient/public representatives. The study is designed to collect

For peer review only - http://bmjopen.bmj.com/site/about/guidelines.xhtml

information on all cases who meet specified criteria (≥18 yrs, deceased person voluntarily resident in Wales and died in Wales or England) whose family were approached between 1/12/15-31/6/17). Data for analysis includes: NHSBT routinely collected anonymised audit data on all cases; Specialist Nurse in Organ Donation (SNOD) completed anonymised form for all cases documenting their perception of the families' understanding of the Act, media campaign, and outcome of the donation approach; Questionnaires and depth interviews with any family member or appointed representative (minimum 50 cases). Additional focus groups and interviews with SNODs. Anonymised donation outcomes and registration activity reports for Wales provide additional context.

Ethics and dissemination

Approved by NHSBT Research, Innovation and Technology Advisory Group (RINTAG) on 23/10/15; Wales Research Ethics Committee 5 (IRAS190066; Rec Reference 15/WA/0414) on 25/11/2015, and NHSBT R&D Committee (NHSBT ID: AP-15-02) on 24/11/2015.

Registration

The protocol is registered on the Health and Care Research Wales Clinical Research Portfolio. Study ID number 34396, www.ukctg.nihr.ac.uk

Key Words: Law, Organ Donation, Implementation, Mixed-method evaluation, Deemed Consent, Wales

Strengths and limitations of this study

- The study is a large scale prospective mixed-method evaluation of the immediate impact of the Act using multiple data sources.
- Previous studies have struggled to recruit family members involved in an organ donation approach.
- The success of this study is dependent on the multiple recruitment strategies and the engagement of NHS Blood and Transplant staff who will primarily recruit participants
- NHS Blood and Transplant is an equal partner in this co-productive study.
- Many Patient and Public representatives and organisations are supporting the study.
- CRUSE Bereavement Care Cymru is supplying bereavement support information for bereaved families who participate in the study.

For peer review only - http://bmjopen.bmj.com/site/about/guidelines.xhtml

For peer review only

For peer review only - http://bmjopen.bmj.com/site/about/guidelines.xhtml

Family attitudes, actions, decisions and experiences following implementation of deemed consent and the Human Transplantation (Wales) Act 2013: Mixed-method study protocol

INTRODUCTION

The Human Transplantation (Wales) Act 2013 introduced a 'soft opt-out' system of organ donation.[1] In an 'opt-out' system presumed consent means that unless the deceased person has expressed a wish in life *not* to be an organ donor then consent will be assumed (or deemed in Wales). There are two types of 'opt-out' system: a 'hard opt-out' where the family are not consulted or a 'soft opt-out' where the family are consulted.[2]

The purpose of the Act is to make it easier for people to donate their organs to benefit patients. The Act is central to the Wales Action Plan[3], which sets out a programme of continuous improvement on all aspects of organ donation and transplantation to deliver the NHS Blood and Transplant (NHSBT) strategy 'Taking Organ Transplantation to 2020'.[4] NHSBT is a Special Health Authority in England and Wales (accountable to the Department of Health) that is responsible for promoting tissue and organ donation to the public and managing organ donation and transplantation. The overall target of the strategy is to increase United Kingdom (UK) consent rates to 80% by 2020. Under the former 'opt-in' system, in 2012/13, 2013/14 and 2014/15 only 50.3%, 53.6% and 48.5% of families consented to deceased donation in Wales.[5] In contrast the consent rate in Spain, which operates an 'opt-out' system in which all citizens are automatically registered for organ donation unless they choose to state otherwise, ranged between 80-85%.[4,6.]

Wales has a devolved parliamentary legislature within the United Kingdom and a population of just over three million people. Responsibility for healthcare legislation is devolved to the Welsh Government. The Human Transplantation (Wales) Act 2013 constitutes one of the biggest changes to the partnership and social contract between the Welsh Government and the people of Wales. The Act is however controversial and not everyone consulted agreed with the 'soft opt-out' system and its principle of deemed consent.[7-8]

Potential donor families are considered to be most affected by the Act as, unlike the old 'opt-in' system, their role in the 'soft opt-out' system remains essential but changed by deemed consent.[9] Under the previous 'opt-in' system, which came under the Human Tissue Act 2004[10], if the individual's consent had not been indicated by the deceased person or a nominated representative, consent was sought from the person who was in a 'qualifying relationship' with the deceased person

For peer review only - http://bmjopen.bmj.com/site/about/guidelines.xhtml

immediately before their death (usually a family member). If the decision regarding donation was unknown then families were less likely to give consent.[9,11] If those close to the deceased person objected to organ donation, for whatever purpose, when the deceased person (or their nominated representative) had explicitly consented, they did not have the legal right to revoke the consent, however the existence of appropriate, valid consent permitted donation to proceed, but did not mandate that it must. The final decision about whether to proceed rested with the medical team when family members did not support donation.

How the intervention is intended to work

In a research context the Act and implementation strategy is conceptualised as a complex behaviour change intervention.[12] The Act changes the principles of consent to deceased organ donation from one of 'opt-in' to a 'soft opt-out' for adults who are 18 years or over; voluntarily resident for 12 months or more in Wales; who have not made an express decision regarding organ donation; and is competent to understand the notion of deemed consent. The individual must also die in Wales for the Act to apply.

NHSBT employ teams of Specialist Nurses in Organ Donation (SNODs) who work across regions to support the organ donation process.[13] The choices individuals now have in either expressing their organ donation decision or choosing to do nothing and having their consent deemed (criteria apply) have impacted on the approach to the family by the SNODs. Once the SNODs have ascertained that the individual has not recorded their organ donation decision on the Organ Donor Register (ODR) and has not appointed a representative to make the decision on their behalf, the conversation with the family is presumptive in favour of organ donation, informing them if applicable their relatives' consent will be deemed to have been given. During the conversation the family are able to inform the SNODs that their relative did not want to be an organ donor. In this circumstance the family are required to produce clear evidence that the person did not want to be an organ donor. The Act is permissive in the sense that it allows for consent to be deemed in certain circumstances, however it does not mandate that organ donation goes ahead in such cases. If an individual has registered a decision or informed someone that they did not want to donate organs prior to their death, their decision will be respected unless the family is able to produce clear evidence that the individual had changed their mind.

For peer review only - http://bmjopen.bmj.com/site/about/guidelines.xhtml

Intended behaviour change

The success of the Act depends on behaviour change (public and professional) to work as intended. The theory is that the neutral media campaigns supporting implementation will facilitate five behaviours:

(1) People will register to 'opt-in' on the organ donor register and appoint a patient representative,

(2) or they register to 'opt-out';

(3) People will discuss their donation decision with families and friends;

(4) People can do nothing and it will be assumed that they do not object to organ donation (deemed consent)

(5) in making the donation decision, families will put aside their own views on donation and respect the decision of the deceased person.

Overall this complex intervention addresses four components of behaviour change as outlined in the Nuffield Council of Bioethics ladder of intervention (Figure 1).[14] The Act and implementation strategy were designed to change the default position so that organ donation became the norm. The Government-led media campaign was however presented in a neutral way to provide people with information to make an informed choice. Nudge theory was also used to underpin behaviour change – such as exposing the population of Wales to a series of 'nudge alerts' via email, Royal Mail, and the media to do specific things such as making their organ donation decision known and 'opting in' or 'out' on the organ donation register.[14] The media did however generally present organ donation as having positive benefits (eg: giving the gift of life).

In addition to the public media campaign, there was an accompanying implementation strategy for NHS and NHSBT staff, which required amending clinical protocols and procedures and retraining large numbers of staff and all SNODs covering Wales. The multiple elements of this complex intervention are shown visually in Figures 2,3,4.

For peer review only - http://bmjopen.bmj.com/site/about/guidelines.xhtml

Modifications to the approach conversation under the Act.

The SNOD facilitates an approach conversation with the family at the point indicated in Figure 5. After the 1st December 2015, for deceased people who have not registered to 'opt-out' on the ODR, the approach to families will be a presumptive conversation in favour of organ donation. The sequence of obtaining consent for deceased organ donation when the patient has not recorded their decision on the ODR is shown in Figure 6. Irrespective of whether the deceased person is registered on the ODR or not - the assumption is that family members will put aside their own beliefs if different to the deceased person and support the express decision to donate or by choosing not to register a decision by any means support their relative's deemed consent.

Prudent healthcare principles

The Act is conceived as a Prudent healthcare policy. Any Prudent health service or intervention is based on the following 4 principles:[15-16]

- Achieve health and wellbeing with the public, patients and professionals as equal partners through co-production. Patient and public contribution is essential to create a patient-centred system for both potential donors and transplant recipients.[15-16] The soft opt-out system has been developed in close consultation with the people of Wales.[17-20]
- Care for those with the greatest health need first, making the most effective use of all skills and resources. The principles underpinning organ transplantation decisions are founded on caring for those with the greatest health need first, irrespective of ability to pay. There is good evidence that all transplants are cost-effective. For example, the cost benefit of kidney transplantation compared to dialysis over a period of ten years (the median transplant survival time) is £241,000 or £24,100 per year for each year that the patient has a functioning transplanted kidney.[21] Although the Act covers all organs and tissues from which patients may benefit from cost-effective transplants; the case for economic renewal and regeneration is best made in Wales by increasing the number of kidney transplants. Kidney transplants are highly cost-effective particularly in relation to NHS spend, and is the treatment of choice for many patients with end-stage renal failure. Recipients can often engage more productively in the economy once they no longer need dialysis.
- Do only what is needed, no more, no less; and do no harm. The 'soft opt-out' is designed to make it easier for the people of Wales to become organ donors. Transplantation is designed to offer patients more options for their treatment with increased benefits that outweigh the risks.

For peer review only - http://bmjopen.bmj.com/site/about/guidelines.xhtml

- Reduce inappropriate variation using evidence based practices consistently and transparently. Attitudes to organ donation vary across Wales and across social gradients and cultures.[20] The purpose of the neutral media campaign is to reduce this variation by providing the public with high quality accessible information.

Rationale for the study

There is evidence from a UK context describing the multiple converging factors that appear to influence donation decisions under the 'opt-in' system, such as knowledge of the deceased's wishes and the view of families that the deceased person had suffered enough.[15-20] We want to specifically explore the perspectives of organ donor registration and deemed consent with families and close friends who were involved in an organ donation decision.

This study is designed to address a critical gap in understanding by exploring if the Act has changed the views and decisions of families. The research is needed to understand donor family responses, which could have an immediate impact on the design of future interventions to change behaviours. Understanding how and why people in reality respond to the 'soft opt-out' will be vital to contextualising the impact of this Prudent health policy in achieving its aims. We want to explore what happened and ascertain the perspectives and decisions made by individuals who were involved in an organ donation decision, and to explore whether the donation decision reflected the patient or family view. There is also a potential benefit to participants as the study provides a confidential independent opportunity to talk about their views and experiences, which in turn can be used to benefit future donor families and patients.

Findings will fill a critical gap in knowledge to supplement the Welsh Government impact evaluation and shed light on the mechanisms that prevent or enhance organ and tissue donation under the new 'soft opt-out' system. Undertaking research to better understand these mechanisms and how they work will be vital for policy makers, healthcare professionals working in NHSBT and the NHS in general. It will inform continuous service improvement to realise the intended outcomes of this very complex intervention (the Act, media-based behaviour change interventions, retraining of NHS and NHSBT staff, and the interventions of NHS and NHSBT teams when requesting consent).

For peer review only - http://bmjopen.bmj.com/site/about/guidelines.xhtml

Aim

The aim of the study is to explore the impact of the Act on consent for deceased organ and tissue donation in the new 'soft opt-out' system. A secondary aim is to further build research capacity in NHSBT and Patient and Public Involvement (PPI) representatives in Wales.

Research questions

1. What impact and changes has the Act and media campaign had on the views and decisions of families of potential organ donors in Wales?
2. What were the views of the deceased person and how did families take account of the deceased person's view in the decision-making process?
3. What are the views of families of the deceased person on the shift in relationship with the Government and healthcare services; organ donor registration; deemed consent; express patient decision and role of appointed representatives; and the changed role of families in decision-making in a 'soft opt-out' system?

Objectives

1. To ascertain a broad overview of anonymised family views, actions and outcomes from organ donation conversations in Wales for an 18 month period following implementation of the Act.
2. To explore in greater depth the perspectives and experiences of families who were involved in a donation conversation.
3. To explore the perspectives of SNODs and their managers covering Wales to contextualise potential donor family views, experiences and decisions.
4. To contextualise findings with Welsh Government survey data and contemporaneous and pervious NHSBT activity reports on organ donor registration and organ donation in Wales.
5. To further develop research capacity and capability in NHSBT and patient and public representatives in Wales.

METHODS AND ANALYSIS

We consulted widely and extensively with multiple key stakeholders to design an ethically defensible and sensitive study that respects the vulnerability and confidentiality of bereaved potential donor families and the dignity of the deceased family member. The four phase design (Figure 7) combines use of routinely collected donor audit activity and national attitudinal surveys as context to a primary study using shared anonymised and routinely collected NHSBT information on decision-making processes and outcomes of the donation consent process, and interviews with Welsh potential deceased donor families/appointed representatives/close friends and organ donation professionals

For peer review only - http://bmjopen.bmj.com/site/about/guidelines.xhtml

covering Wales (See Supplemental File Appendix 1 for a summary of all data sources contributing to the analysis). Recruitment and data capture targets for each phase are shown in Figure 7.

Phase 1 – Primary study to gain a high level understanding of the impact on donor family responses (accepting the patient's decision, or consenting to, or not consenting to donation) for 18 months from 1st December 2015.

SNODs will complete an anonymised electronic 1 page form [Supplemental File Appendix.2: Form B] for every approach conversation that will be filled out as soon as possible after they have disengaged from speaking with the potential donor family. No participant or patient identifiable information will be recorded. SNODs will complete the form (electronic or paper) using information gathered from their routine conversations with potential donor families.

Phase 2: Primary study with potential deceased donor families/appointed representatives and close friends to ascertain a deeper understanding of their thoughts, experiences and responses to the Act and their decision-making.

Family members/appointed representatives and close friends, directly or indirectly involved in the donation process, will be invited to self-complete an anonymised questionnaire [Supplemental File Appendix 3: Form C] that requires no contact with the research team. There is no restriction on the number of questionnaires per family and the questionnaire will take around 20 minutes to complete. Accompanying the questionnaire will be an invitation to participate in an interview to discuss their views and experiences in greater depth and a contact form to send back to the independent research team to arrange a mutually convenient interview (Supplemental File Appendix 4: Family interview schedule). For those wanting also to participate in an interview, several options will be offered that best suit the individual, such as face to face, telephone or via social media. Mindful that participants have been bereaved, they can select the time that is right for them to be interviewed up until the end of the period of data collection.

Recruitment of family/close friend/appointed representative

We will use a range of methods that are sensitive and individually tailored to recruit participants involved in a minimum of 50 potential organ donation cases, with maximum variation to cover all donation pathways and outcomes. SNODs will use their discretion and knowledge of the family to select the most appropriate options and times to share information about the study with families/appointed representatives. Recruitment options include via direct contact with families by SNODs (with the option to using consent to contact form), and sharing study information in person;

For peer review only - http://bmjopen.bmj.com/site/about/guidelines.xhtml

and by sending out a study invitation with information attached to routine follow-up communication by NHSBT; by direct mailing of study invitation and information by NHSBT; via adverts in the media, and through snowball sampling.

If an individual receives more than one letter of invitation we will include a sentence to explain that, if they have already made their decision whether to participate in the study or not, they can ignore the letter or pass the invitation onto another family member or close friend of the deceased person, because NHSBT only have one contact name for each family. For participants who would prefer to be interviewed in their first language (Welsh or other language) we have employed a Welsh medium research officer and have built in interpreter costs.

Inclusion criteria for 'family' participant recruitment

- Any person over 16 years with mental capacity who was involved, either directly or indirectly, in a deceased organ donation conversation or decision in Wales after 1st December 2015.
- Any person over 16 years with mental capacity who was involved, either directly or indirectly, in a deceased organ donation conversation or decision of a Welsh resident who died in a hospital in England after 1st December 2015 and was managed by the NHSBT organ donation teams covering North or South Wales.
- Any close friends of the deceased person who may want to share their perspectives on the donation process and outcome
- Any NHSBT SNODs and managers covering Wales.

Exclusion criteria for 'family' participant recruitment

- Under 16 years
- Lacking mental capacity
- Potential donor was a Welsh resident over 18 years who died in an English hospital not covered by participating SNODs

For peer review only

Phase 3: Primary Study with qualitative 1:1 or small or focus group interviews with NHSBT organ donation teams covering Wales (SNODs and managers in our co-productive project) to contextualise potential donor family decision making, reactions and responses to the Act.

SNODs and their managers will be invited by letter with accompanying study information to participate in 1:1 and small or focus group interviews at the end of the study to contextualise the findings. Interviews will be at the end of the study and last approximately 60 minutes.

Phase 4: Comparative analysis and overarching synthesis of stages 1-3.

Study data will be analysed and findings contextualised with descriptive numerical data and additional nattative data shared by NHSBT and Welsh Government (See Supplemental File Appendix 1 for a summary of data sources).

Data Analysis

Narrative textual data

With consent, interviews will be digitally recorded and transcribed verbatim in the original language. The Framework approach for analysis of applied policy research will be used for all narrative textual data from questionnaires, focus groups and interviews).[22]. We will use NVivo software version 11[23] to facilitate the Framework analysis.[22] First, the initial 5 available verbatim free text in questionnaires and interview transcripts will be read and reread and key themes and categories will be identified. The definitions and boundaries of each of the emerging themes for each type of evidence from questionnaires, focus group and interviews will then be discussed to see how these can be developed to form an a priori framework tailored for either questionnaires or transcripts in Welsh and English. Searching for additional themes will continue until all text from questionnaires and interview transcripts have been analysed and no new themes are discerned. Following analysis of Welsh language text, themes and relevant quotes will then be translated into English and back translated.

The final themes and their dimensions for text from questionnaires and interviews will then be further refined and used as the basis of charts (or matrices), which allows for themes to be compared and displayed for questionnaires and interviews, and for variations and deviant cases to be highlighted within each dataset. These charts will be overlaid with key information to preserve the original context. Secondly, these charts will undergo several revisions and further refinements, in an iterative process moving between the charts and the themes identified from questionnaires and

For peer review only - http://bmjopen.bmj.com/site/about/guidelines.xhtml

interviews, until it is possible to synthesise the key findings across the datasets in a set of overall themes or categories. This stage will involve what is sometimes called the translation of themes from one data source to another. In the process of comparing the themes, we will look for explicit differences in relation to a range of factors that impact on decision-making including gender, relationship of the person to the deceased, age, ethnicity, and whether consent for donation was given or not and whether registration as an organ donor was viewed positively or negatively.

Categorical questionnaire data

Completed form B and Cs will contain structured categorical options (such as the Yes, No, Uncertain), which will be collated in SPSS version 22 and analysed using descriptive statistics.[24] Results will be displayed as numbers and percentages.

Comparative analysis and overarching synthesis

We will use Oliver's synthesis framework for juxtaposing evidence across phases 1-3 with Welsh Government omnibus surveys and contemporaneous and previous NHSBT activity reports listed in Supplemental File Appendix 1.[25] We will organise data by donation decision (opt in opt out on organ donation register; expressed decision, deemed consent) mapped against whether families supported the donation decision and why. We will layer the descriptive numerical and narrative findings onto the framework to synthesize findings across the different types of evidence, working within each of the spheres of influence (the patient decision, family, NHSBT, NHS and clinical care, the law, the media campaign, previous comparative data etc). Juxtaposing different numerical, narrative and temporal evidence in this way on the same phenomenon of interest will enable us to look for patterns, explanations, mechanisms and disconfirming cases.

As there is not a specific reporting guideline for mixed-method studies, we will draw on new guidance for reporting mixed-method syntheses[26] and the Consolidated Criteria for Reporting Qualitative Research (COREQ) guidelines.[27]

Ethics and dissemination

This protocol was approved on 23/10/15 by NHSBT Research, Innovation and Technology Advisory Group (RINTAG). The study was approved by the Wales Research Ethics Committee 5 NHS research ethics committee (IRAS number 190066; Rec Reference 15/WA/0414 on 25/11/2015) and the NHSBT Research and Development Committee (NHSBT ID: AP-15-02 on 24/11/2015).

For peer review only - http://bmjopen.bmj.com/site/about/guidelines.xhtml

The design and methods are informed by an ethical framework developed by UK-based researchers for undertaking research with family members who are approached about organ donation and draws on the experiences of researchers working with the bereaved.[28-36] Independent governance will be provided by a steering group.

A key component of the ethical nature of the study will be the professional development and training elements to support SNODs and research officers to conduct the study in a respectful and sensitive way. We will dovetail the bereavement support offered by researchers to participants with the bereavement services offered by intensive care units in NHS Health Boards where potential organ donors are cared for with their families, and information provided on bereavement support services shared by SNODs in NHSBT teams covering Wales. In addition, in any contact with participants we will share a bilingual information leaflet on CRUSE Bereavement Care Cymru, in case families would prefer to access free support and counselling outside of a NHS context. In appreciation of their support, research team members will plan a fundraising activity during the study to make a donation to Cruse Bereavement Care Cymru.

Additional information concerning the specific ethical and data protection[37] issues, proposed strategies and data sharing agreement can be found in Supplemental File Appendix 5.

Patient and Public Involvement

Prior to commencement of the study, contextual baseline engagement with the public has consisted of six discussion groups and seven face to face interviews involving fifty-two participants. This contextual work was undertaken by the Welsh Government.[19] Each group was recruited to include a mix of people in terms of awareness of the NHS Organ Donor Register and included some who had joined the Register and/or carried a donor card. Black and Minority Ethnic people formed part of the sample and included Pakistani, African Caribbean, Nigerian and Chinese participants. Each group contained a mix of men and women and the sample was broadly stratified by age and socioeconomic grouping. Two groups were conducted in the Welsh language. In addition, 1006 members of the public responded to a baseline Welsh Omnibus attitudinal survey.[8] Patient and public involvement representatives were involved in prioritising the question and in deciding to fund the study. The leading charities for supporting deceased organ donor families and people with kidney failure requiring a transplant have helped shape the design and advised on appropriate methods of data collection.

The Welsh Government hosted a conference in September 2015 involving those affected by deceased organ donation and health care professionals involved in the donation process to explore the implementation and implications of the Act from different perspectives and to explore how best

For peer review only - http://bmjopen.bmj.com/site/about/guidelines.xhtml

to evaluate the Act and what outcomes from different perspectives are important. These perspectives have been incorporated into the study design. PPI will continue during the study through to dissemination.[38-9]

Building research capacity

A secondary aim of the study is to increase the confidence and capacity of NHSBT and PPI representatives to collaborate in future studies in this field.

In following Prudent healthcare principles,[15-6, 39] we will use a co-production approach, which means that the research team will work as equal partners and in collaboration with NHSBT who have a remit to support relevant research activity, and with a range of key professional stakeholders and PPI representatives to conduct the study. The co-production element is critical to the success of the study and will involve a strong research training and capacity building component for NHSBT teams and PPI representatives working in Wales. We have worked closely with policy and clinical leads from Welsh Government and NHSBT to ensure that the proposed co-productive methods of data collection and participant recruitment are feasible, sensitive to the needs of potential donor families, and NHS staff, and fulfil the high ethical and data protection requirements for data sharing between two organisations.

Three development opportunities will bring NHSBT staff and PPI representatives together. At the beginning of the study we will facilitate professional development meetings with SNODs and managers to design the data collection tools. At the end of the first year of data collection, we will present initial findings at a professional development meeting with collaborating staff from NHSBT, clinical co-applicants, policy makers and PPI representatives to see what shared learning could be used to further enhance practice development and support study data collection. We will facilitate another meeting at the end of the study to present key findings.

Expected Outcomes

The most important outcome will be a research-informed and clearer, shared understanding of deceased donor consent decisions, and in particular the reasons why people continue to refuse to support consent in a ‘soft opt-out’ system, to feed back into further policy and practice development. In addition, staff in NHSBT covering Wales and PPI representatives will have developed additional confidence and research capacity and capability to undertake further and equally challenging studies.

For peer review only - http://bmjopen.bmj.com/site/about/guidelines.xhtml

Impact and Dissemination

The study has potential for high impact as success of this Prudent health policy is dependent on the people of Wales engaging with the principles of deemed consent and donor registration and honouring the deceased person's donation decision. If sufficient people agree and change their behaviour to favour the principles of the 'soft opt-out', then the policy will likely realise the anticipated benefits for patients. If sufficient people disagree then nothing will change and the anticipated increased number of patients who benefit from cost-effective transplants will not be realised.

Understanding why people do not register on the organ donor register or why family/appointed representatives still contest the decision to donate made by the deceased person will have an impact on the design of future interventions to improve organ and tissue donation rates in Wales.

The main mechanism of dissemination, knowledge transfer and maximising impact is through the uptake of project outputs by policy makers, clinicians and the public through a co-productive continuous quality improvement approach in line with Prudent healthcare principles.[15-16] There are key elements known to affect the resources required for managing a successful co-productive dissemination processes and these elements are built into the project design, including:

- motivating change: creating readiness for change and overcoming resistance;
- creating a vision: mission, valued outcomes and conditions, midpoint goals and feedback;
- developing political support: assessing change agent power, identifying key stakeholders, influencing stakeholders;
- feeding back findings and jointly determining their meaning for various stakeholders;
- sustaining momentum: providing resources for professional development and research capacity building, building support systems for change agents, developing new competencies and skills, reinforcing new behaviours.[40]

Other effective elements of knowledge transfer include publication of research results in leading journals, and presentations at local, national and international conferences in the field. The research team has already demonstrated a high quality publication record, and will continue to do so, adopting an open access policy. We will also produce bilingual lay summaries.

For peer review only

For peer review only - http://bmjopen.bmj.com/site/about/guidelines.xhtml

REFERENCES

1. Human Transplantation Act (2013) Wales. http://www.legislation.gov.uk/anaw/2013/5/contents/enacted (Accessed 11th April 2017).
2. Bramhall S. Presumed consent for organ donation: a case against. Ann R Coll Surg Engl. 2011 May; 93(4): 270–272. doi: 10.1308/147870811X571136b
3. Welsh Government. Taking Organ Transplantation to 2020 – Wales Action Plan A plan up to 2020 for NHS Wales and its Partners Ensuring every organ donation counts. http://gov.wales/docs/dhss/publications/140107organactionen.pdf (Accessed 11th April 2017).
4. NHSBT. Taking Organ Transplantation to 2020. http://www.nhsbt.nhs.uk/to2020/resources/nhsbt_organ_donor_strategy_long.pdf. (Accessed 11th April 2017).
5. Personal Communication Karen Morgan, Major Health Conditions Policy Team, Directorate of Health Policy, Health and Social Services Group, Welsh Government. (April 2017).
6. Palmer M. (2012) Opt-out systems of organ donation: International evidence review. Social research Number: 44/2012 Welsh Government, Cardiff.
7. Welsh Government. OPTIONS FOR CHANGES TO THE ORGAN DONATION SYSTEM IN WALES CONSULTATION PAPER. 2009 http://www.wales.nhs.uk/sites3/Documents/773/Organ%20Donation%20consultation%201doc%20-%20English.pdf. Accessed 11th April 2017.
8. Welsh Government. Public Attitudes to Organ Donation: Baseline Survey 2012 Social research Number: 38/2012 Knowledge and Analytical Services Welsh Government Welsh Government, Cardiff. http://gov.wales/docs/caecd/research/121019organdonationen.pdf (Accessed 11th April 2017)
9. Palmer M. (2012) The role of families in organ donation: International evidence review. Social research Number: 45/2012, Welsh Government, Cardiff.
10. Human Tissue Act 2004. https://www.hta.gov.uk/policies/human-tissue-act-2004. (Accessed 11th April 2017).
11. Walker W. Broderick A. and Sque M. (2013) Factors influencing bereaved families' decisions about organ donation: An integrative review of the literature. Western Journal of Nursing Research,Doi.10.1177/0193913484987. 35: 10, 1339-1359.
12. MRC Framework for developing and evaluating complex interventions: New Guidance 2006. https://www.mrc.ac.uk/documents/pdf/complex-interventions-guidance/. (Accessed 11th April 2017)
13. NICE clinical guideline 135 Organ donation for transplantation: improving donor identification and consent rates for deceased organ donation. https://www.nice.org.uk/guidance/cg135/evidence/full-guideline-184994893 (Accessed 20th June 2017).
14. Local Government Association. Changing behaviours in public health To nudge or to shove? Local Government Association. 2013. http://local.gov.uk/sites/default/files/documents/changing-behaviours-publi-e0a.pdf. (Accessed 11th April 2017)
15. Mark Drakeford, Minister for Health and Social Services (2014) Written Statement – Delivering Prudent Healthcare in Wales. http://gov.wales/about/cabinet/cabinetstatements/2014/Prudenthealthcare/?lang=en. (Accessed 11th April 2017)
16. Aylward M, Phillips C, Howson H. (2013) Simply Prudent Healthcare – achieving better care and value for money in Wales – discussion paper. Bevan Commission Simply Prudent Healthcare 4 December 2013.v1. http://www.ctrtraining.co.uk/documents/BevanCommissionSimplyPrudentHealthcarev104122013.pdf. (Accessed 11th April 2017)
17. Organ Donation Wales website. http://organdonationwales.org/FAQs/Organ-donation-from-december-2015/?skip=1&lang=en (Accessed 11th April 2017)
18. McAllister F & Blunt A. Research to support Wales' organ donation opt-out proposal consultation. Qualitative research report Social research Number: 14/2012. Welsh Assembly Government. http://gov.wales/docs/caecd/research/120418organdonationen.pdf. (Accessed 11th April 2017).
19. GfK NOP. Evaluation of the Human Transplantation (Wales) Act: Baseline Survey of NHS Wales Staff. Social research Number: 68/2014 Welsh Assembly Government. http://gov.wales/docs/caecd/research/2014/140707-evaluation-human-transplantation-wales-act-survey-nhs-wales-staff-en.pdf. (Accessed 11th April 2017)

For peer review only - http://bmjopen.bmj.com/site/about/guidelines.xhtml

20. Choudhery R. Evaluation of the Human Transplantation (Wales) Act: Qualitative Study of Public Attitudes 87/2014 Welsh Assembly Government. http://gov.wales/docs/caecd/research/2014/141007-human-transplantation-act-qualitative-study-public-attitudes-en.pdf. (Accessed 11th April 2017)
21. NHS Blood and Transfusion (2014) Cost-Effectiveness of Transplantation. http://www.organdonation.nhs.uk/newsroom/fact_sheets/cost_effectiveness_of_transplantation.asp (Accessed 11th April 2017)
22. Ritchie J. & Spencer L. (1994) Qualitative data analysis for applied policy research. In: Analysing Qualitative Data (Bryman A. & Burges R. eds.), pp. 173-194.
23. NVivo version 11. http://www.qsrinternational.com/nvivo-product (Accessed 20th June 2017)
24. IBM SPSS Statistics V22.0, https://ibm-spss-statistics-base.en.uptodown.com/windows. (Accessed 11th April 2017)
25. Oliver S, Harden A, Rees R: (2005) An emerging framework for including different types of evidence in systematic reviews for public policy. Evaluation 2005, 11(4):428–446.
26. Flemming K et al (2017 in press) Cochrane Qualitative and Implementation Methods Group Guidance Paper 5: Reporting guidelines for qualitative, implementation and process evaluation evidence syntheses. Journal of Clinical Epidemiology.
27. Tong A, Sainsbury P, Craig J. Consolidated criteria for reporting qualitative research (COREQ): a 32-item checklist for interviews and focus groups. Int J Qual Health Care. 2007;19(6):349-357.
28. Jansen N.E. McDonald M. Haase-Kromwijk BJJM. Sque M. and Long-Sutehall T. (2014) When are bereaved family members approached for consent to organ donation? Commentary from 10 European member states. Organs, Tissues and Cells, 17, 101-113.
29. Sque M. Walker W. and Long-Sutehall T. (2014) Research with bereaved families: A framework for ethical decision-making. Nursing Ethics doi:10.1177/0969733014521097.
30. Sque M, Long T, Payne S. (2003) Organ and tissue donation: Exploring the needs of families Summary of the Final. Sponsored by the British Organ Donor Society Funded by the National Lottery Community Fund February 2003. https://eprints.soton.ac.uk/191475/1/BODY_study.pdf (Accessed 11th April 2017)
31. Sque M, Long T, Payne S, Allardyce D. Why relatives do not donate organs for transplants: 'sacrifice' or 'gift of life'? J Adv Nurs. 2008 Jan;61(2):134-44. doi: 10.1111/j.1365-2648.2007.04491.x.
32. Sque M. and Galasinski D. (2013) ‘Keeping her whole’: bereaved families' accounts of declining a request for organ donation. Cambridge Quarterly of Healthcare Ethics, Special Edition, 22: 1, 53-63.
33. Jansen NE. Haase-Kromwijk BJJM. McDonald M. and Sque M. (2013) Elements of the European jigsaw: the timing of the approach to bereaved families for consent to DBD or DCD donation and the implications for effective donation campaigns. In Randhawa G. and Schicktanz S. (Eds.) Public engagement in organ donation and transplantation. Lengerich, Germany: PABST Science Publishers, 161-169.
34. Long-Sutehall T. Winstanley E. Clarkson A. and Sque M. (2012) Evaluation of the experiences of family members whose deceased relative donated tissues at the NHSBT dedicated donation facility in Speke, Liverpool. Cells and Tissue Banking, 13: 4, 537-546.
35. Sque M. and Long T. (2011) Bereavement, decision-making and the family in organ donation. In Farrell AM. Price D. and Quigley M. (Eds.) Organ shortage: principles, pragmatism and practice, Cambridge: Cambridge University Press, 67-85.
36. Sque M. and Payne. S. (Eds.) (2007) Organ and tissue donation: an evidence base for practice. Maidenhead: Open University. Australian Journal of Medical Science 29: 1, 20.
37. The Data Protection Act 1998. Reviewed March 2017 http://www.legislation.gov.uk/ukpga/1998/29/contents (Accessed 11th April 2017)
38. NIHR Involve (2014) Policy on payment of fees and expenses for members of the public actively involved with INVOLVE August 2014 http://www.invo.org.uk/wp-content/uploads/2014/11/INVOLVE-internal-payment-policy-FINAL-August2014.pdf. (Accessed 11th April 2017)
39. Jung T, Harrow J and Pharoah C (2012) Co-producing research: working together or falling apart? CGAP Briefing Note 8.http://www.cgap.org.uk/uploads/Briefing%20Papers/CGAP%20Briefing%20Note%208%20-%20Co-producing%20research.pdf. (Accessed 11th April 2017)
40. French, W. and Bell, C. (1999) Organization Development. New Jersey, USA: Prentice Hall.

For peer review only - http://bmjopen.bmj.com/site/about/guidelines.xhtml

List of Figures

Figure 1. Nuffield Council of Bioethics ladder of intervention.
Figure 2. Intervention implementation: Multi-facetted media-based strategy to inform the public of the Act and changes to consenting to organ donation.
Figure 3. Intervention and required behaviours following introduction of the Act
Figure 4. Intervention implementation: Additional training for Specialist Nurses in Organ Donation when approaching families following the implementation of deemed consent.
Figure 5. . Wales potential organ donor population and identification of the 'donation request' stage in the process.
Figure 6 Sequence of obtaining consent when the patient is NOT on the Organ Donor Register (ODR).
Figure 7. The four phase study design.

Supplementary Files: List of Appendices

Supplemental File Appendix 1. Summary of datasets and evidence contributing to the mixed-method evaluation
Supplemental File Appendix 2. FORM B: Completed by Specialist Nurse in Organ donation**.**
Supplemental File Appendix 3. FORM C: Questionnaire completed by family members/close friends.
Supplemental File Appendix 4. Interview schedule for families.
Supplemental File Appendix 5. Ethical issues and strategies.

Author contributions

Jane Noyes – Chief Investigator conceptualised the idea, put the team together, designed the study and procedures, and drafted the protocol.

Michael Stephens - Consultant Transplant and Organ Retrieval Surgeon, Clinical Lead for Transplantation, Cardiff and Vale Health Board - advised on key research team members and stakeholders to bring into the research team, proposed changes in the law and key research questions to address.

Karen Morgan – Formerly Regional Manager South Wales and South West, NHSBT and now Major Health Conditions Policy Team, Directorate of Health Policy, Health and Social Services Group, Welsh Government – advised on key changes to policy and practice, study design and processes, data collection tools and implementation of the study.

Phillip Walton – Regional Manager South Wales NHSBT advised on on changes to policy and practice, study design and processes, data collection tools, and implementation of the study.

Abigail Roberts – Specialist Nurse in Organ Donaiton NHSBT advised on the role of the Specialist Nurse in Organ Donation, study design and processes, data collection tools and implementation of the study.

Leah Mclaughlin – Research Officer – finalised study procedures and data collection processes, designed the study documentation and logos and supported production of applications to the NHS REC and NHSBT R&D committees.

For peer review only - http://bmjopen.bmj.com/site/about/guidelines.xhtml

All authors agreed the final manuscript.

Acknowledgments

Fiona Wellington – Head of Operations NHSBT for supporting the study.

Christian Brailsford – NHSBT provided advice and support to agree a mutual data sharing agreement and negotiate NHS ethics and NHSBT RINTAG and NHSBT R&D processes.

Pat Vernon (Policy Lead Welsh Government), Ian Jones (Research and Evaluation Lead) Caroline Lewis (Organ Donation Policy Manager) provided a Government perspective and shared research carried out prior to implementation of the Act.

Donald Fraser – Lead of the Wales Kidney Research Unit supported development of the funding application and serves as independent Chair of the steering group.

Jo Mitchell – research support officer

North West NHSBT Team: Ben Armstrong, Adam Barley Angela Campion-Sheen, Laura Ellis-Morgan, Rebecca Gallagher, Sharon Hallam, Phil Jones, Andrew Mawson, Abi Roberts, Tracey Rhodes, Helen Bullock, Andrea Jones, Kathryn Alletson, Jane Monks, Emma Thirlwall, Dawn Lee, Nicky Hargreaves, Lisa Welsh, Gill Drisma, Sue Duncalf.

South Wales NHSBT Team: Angharad Griffiths, Lucy Barnes, Charlotte Goodwin, Guy Heathcote, Gail Melvin, Michael Tobin, Lisa Morgan, Nicola Newbound, Michelle Powell Stephen Regan, Fiona Rogers, Susie Cambray, Kathy Rumbleow, Lynne Woolcocks, Janet Woodley, Beth Moss, Louise Colson.

NHSBT Staff: Sian Griffin (Consultant Nephrologist, Department of Nephrology and Transplantation) Katja Empson, Sam Sandow, Carl Stephenson (Clinical Leads Organ Donation) Francesca Stevens (Tissue Services NHSBT), Maggie Stratton (PR Officer NHSBT).

Jeanette & CRUSE Bereavement Care – who provided leaflets signposting bereavement support for participants.

Patient and Public representatives: Sarah Thomas, Janet Thickpenny, Gethin Rhys, Michael Rhys, Maria Mesa, Roon Adams, Michael Houlston, Maria Battle, Anna Bates for providing guidance and advice on the focus of the study.

Janet Williams and Gloria Owe for providing advice and feedback on processes, participant facing documents, and the detailed funding application which contained this protocol.

Patient and Public representative organisations: Flintshire Deaf Association, Llanelli Multi Cultural Network, Big Lottery, Churches Together in Wales, Women Connect First, BAWSO, Race Equality First, Believe, Donor Family Network, Rita's Café for helping to set up the patient and public network and supported the study proposal.

Gareth Wyn Roberts - Consultant Nephrologist, Cardiff and Vale University Health Board provided detailed advice on clinical processes and the new Act.

Catherine Robinson – Former Head of School of Social Sciences, Bangor University, supported submission of the funding application following high level discussions and commented on a section of the application.

For peer review only

Funding statement

The study was funded by Health and Care Research Wales. Project Reference 1129.

Competing interests statement

There are no known competing interests.

For peer review only

For peer review only - http://bmjopen.bmj.com/site/about/guidelines.xhtml

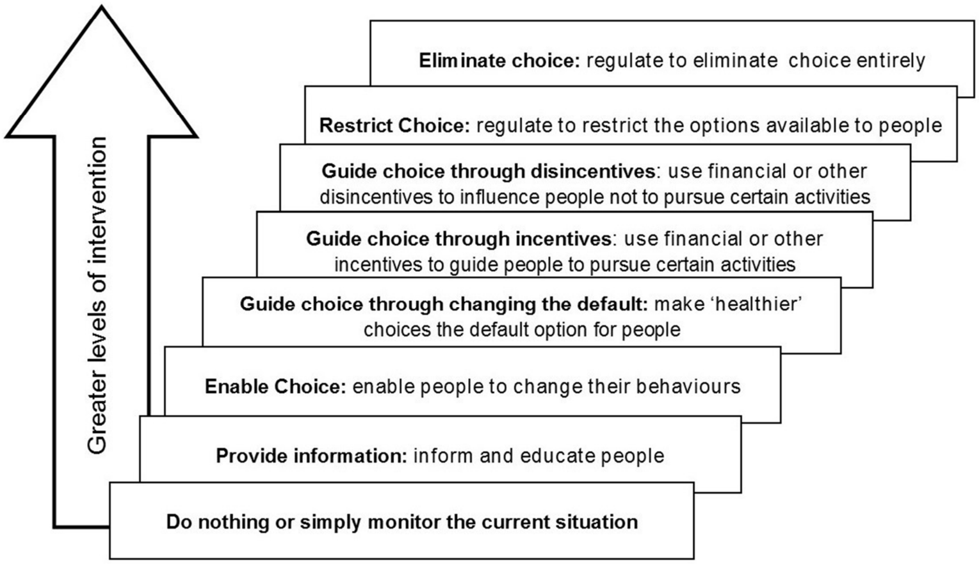

Figure 1. Redrawn from original. Nuffield Council of Bioethics ladder of intervention.

197x132mm (300 x 300 DPI)

view only

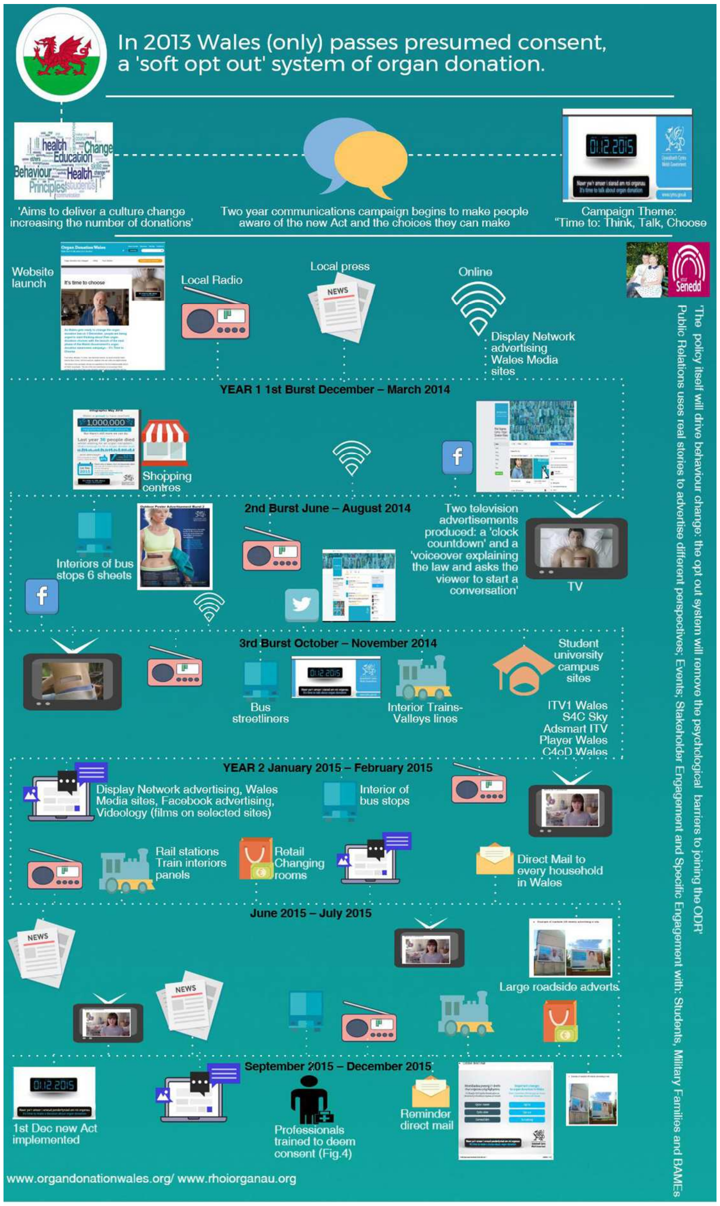

Figure 2. Multi-faceted media-based implementation strategy.

402x677mm (300 x 300 DPI)

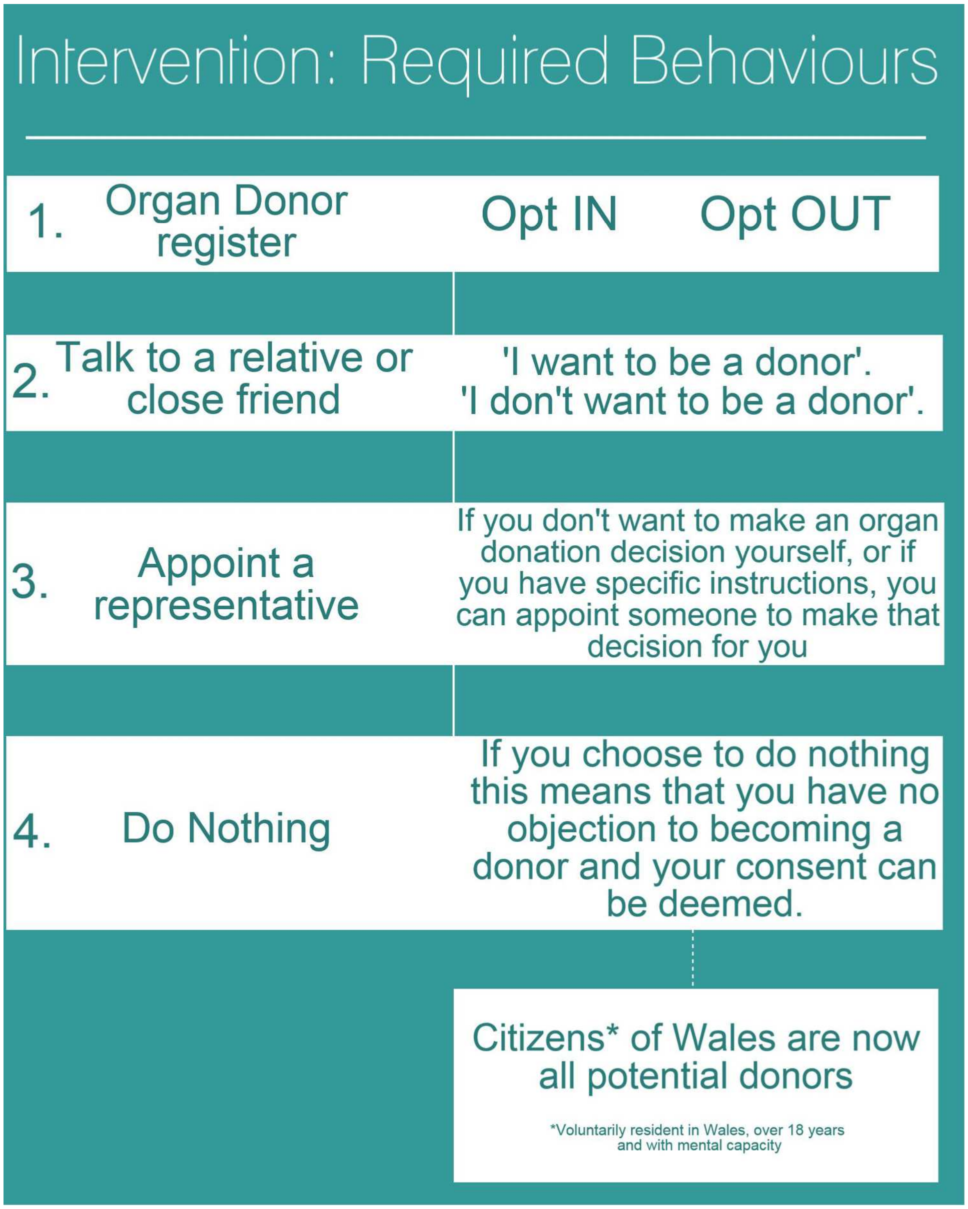

Figure 3. Intervention and required behaviours following introduction of deemed consent.

474x591mm (300 x 300 DPI)

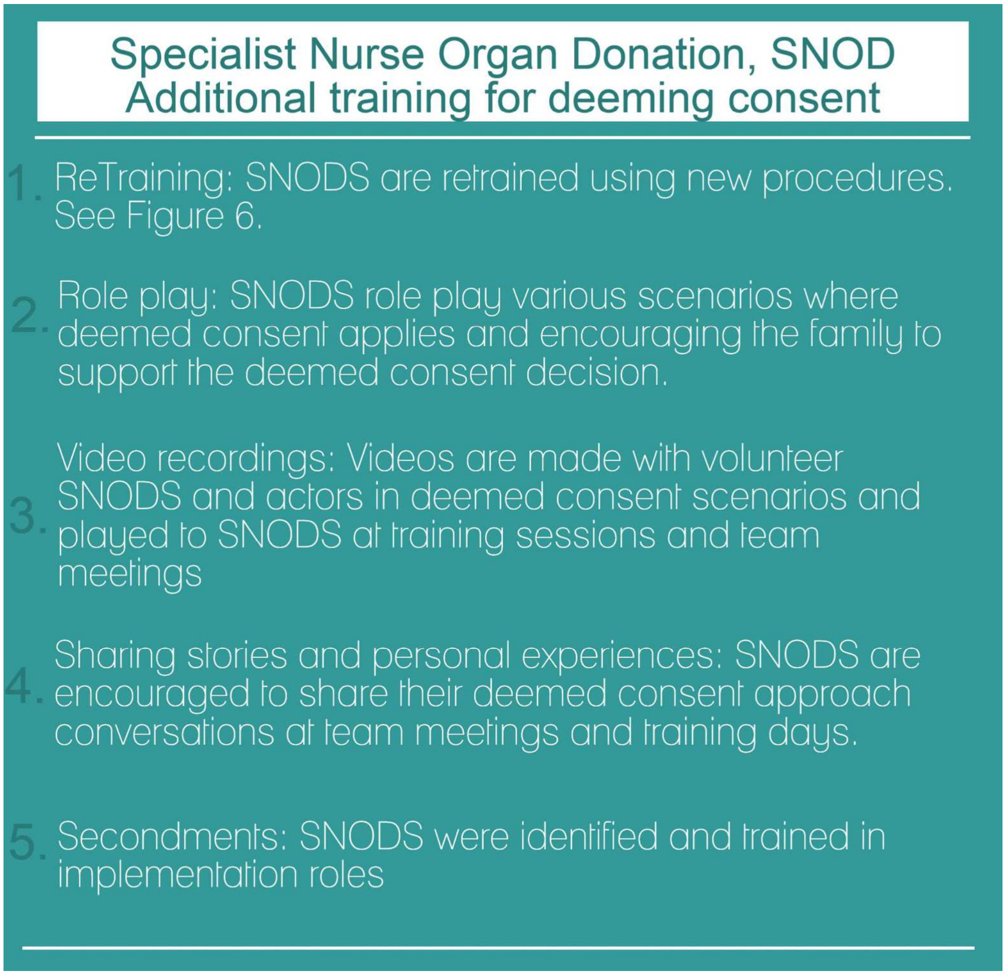

Figure 4. Specialist Nurse in organ Donation additional training.

541x522mm (300 x 300 DPI)

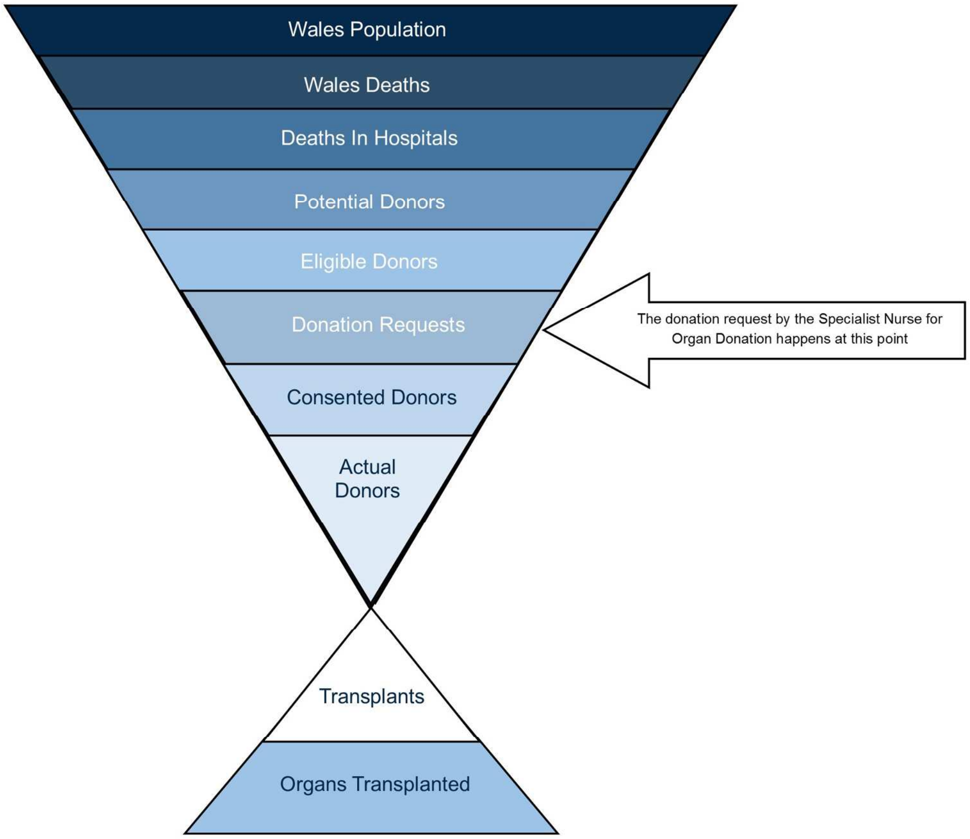

Figure 5. Wales potential organ donor population and identification of the 'donation request' stage in the process.

345x296mm (300 x 300 DPI)

only

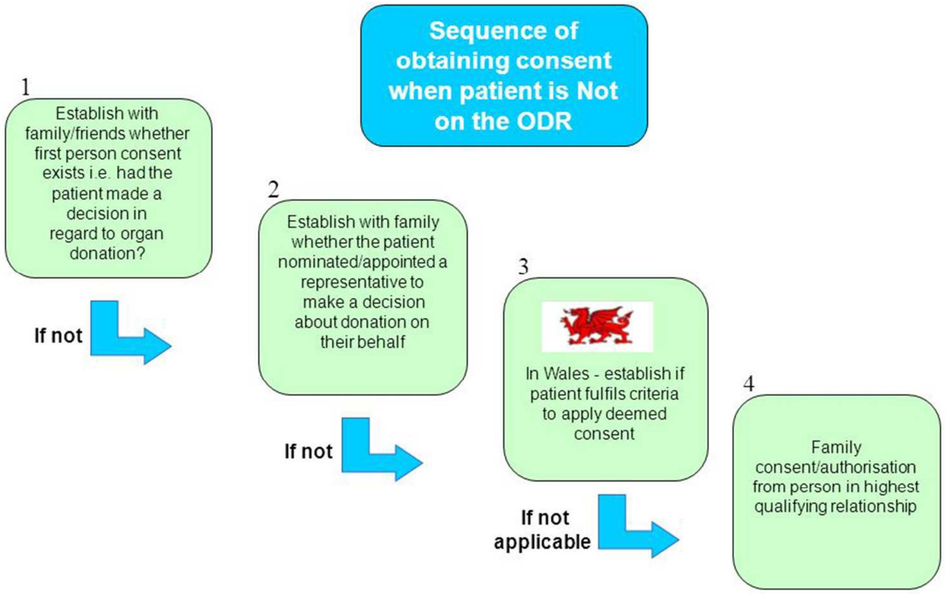

Figure 6. Sequence of obtaining consent when the patient is NOT on the Organ Donor Register (ODR) after 1.12.15. Image reproduced from NHSBT document using 'Fair use' clause.

175x131mm (600 x 600 DPI)

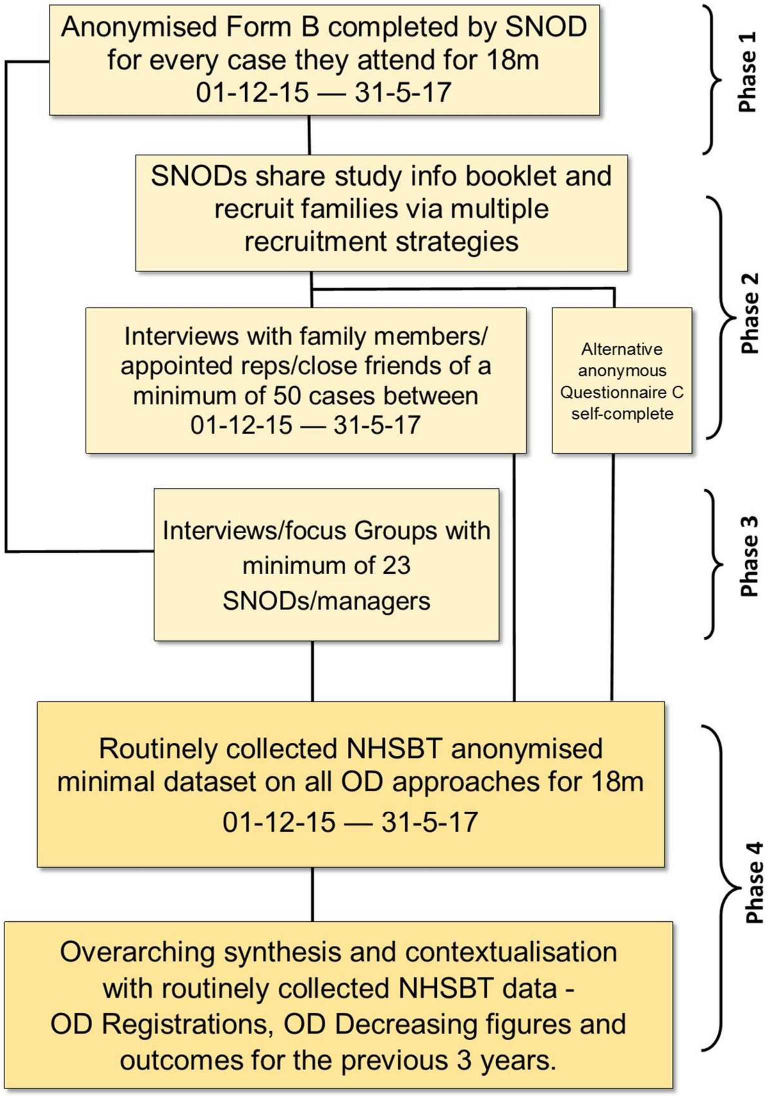

Figure 7. The four phase design. Key: NHSBT: NHS Blood and Transplant. OD: Organ Donation. SNOD(s): Specialist Nurse(s) in Organ Donation.

209x297mm (300 x 300 DPI)

Appendix 1. Summary of datasets and evidence contributing to the mixed-method evaluation

	Dataset/Evidence	Content
		**Anonymous data shared by NHSBT under data sharing agreement**
1	**Anonymised NHSBT Log.**	The log records details of all approach conversations that Specialist Nurses in Organ Donation had with a potential donor family for whom the Act applied over the data collection period (18 months) 01/12/15 -31/05/17. It was created specially to capture specific details of the consent conversation after the law changed in Wales. The log includes:<br>1. A record of whether the deceased died via a Donation by Brain Stem Death (DBD) or Donation by Circulatory Death (DCD).<br>2. The deceased person's registered status on the Organ Donor Register (ODR) – Registered In/out or no registration found.<br>3. Type of Consent – Organ Donation Register In/Out, Expressed Consent In/Out, Deemed Consent and family consent (for those who did not fulfil the criteria to have their consent deemed).<br>4. Patients expressed decision – donate all organs, does not want to donate, no decision made.<br>5. Who the SNOD had the conversation with.<br>6. Did the family accept the known decision of the deceased person.<br>7. Reason why family objected to the known decision or the deemed consent.<br>8. If organ donation proceeded – the comments in number 11 will document if the donation stood down due to a medical reason or via the influences of the family, see number 11.<br>9. Who undertook the donation conversation.<br>10. Did family know about the Welsh Legislation.<br>11. Comments (to include evidence/information provided by families who are unable to support known decision/deemed consent).<br>12. Feedback/additional training requirements to staff – did this particular case highlight any areas for further professional development training.<br><br>Descriptive statistics report totals for categorical data.
2	**Summary statistics from NHSBT for the 18 month data collection window for Wales only.**	NHSBT summary of descriptive statistics specially prepared for the research team to cover the data collection window (01/12/15-31/05/17). Includes summary data on: organ donation registration; consent and deemed consent numbers; age range; ethnicity and reasons why donation not proceeded.
		**Routinely collected and publicly available NHSBT data**
3	**Publicly Available NHSBT Audit Data (Wales).**	NHSBT annual audit data runs from 01st April – 31st March and is available online for current and previous years. Relevant data mapped onto this study includes: Organ Donation Registration data; Number of deceased donors; Consent rates and deemed consent rates.

	**Continuous annual audit.**	
4	**Publicly Available NHSBT Audit Data (UK). Continuous annual audit.**	As above data also includes UK figures for:<br>Reasons why consent not given/decision not supported<br>Age, Ethnicity, gender of donors.
5	**Organ Donation Register UK.**	A new UK organ donor register was introduced in July 2015. People have the opportunity to Opt in, Opt Out and appoint a representative. Registration behaviour figures and trends will be used to contextualise study findings.
		**Publicly Available Welsh Government Commissioned Research**
6	**Focus groups with SNODS**	Welsh Government commissioned three sequential focus groups with SNODS, before, immediately after implementation and a year after the changes were introduced. Final focus group findings shared ahead of publication.
7	**Ombudsman Surveys**	Welsh Government commissioned 12 sequential public opinion surveys undertaken with the Welsh public in the years before and after the law changed. Wave 10 of the survey focused on monitoring awareness levels and understanding of the change in law and included additional questions to measure awareness and recall of publicity campaign material. Wave 11 and 12 focused on awareness and understanding as well as attitudes and behaviour.
8	**Literature reviews**	Systematic reviews of the literature on family attitudes to organ donation and reasons why donation is declined.
		**Additional data collection by the research team**
9	**Anonymous Family, Questionnaire FORM C.**	Families are sent a questionnaire capturing basic information on their understanding of the changes and their feelings about supporting their loved one's donation decision. Appendix 3. FORM C: Questionnaire completed by family members/close friends.
10	**Interviews with families**	Depth Interviews with families of a minimum of 50 cases to explore their views on organ donation, the Act, the media campaign and their donation experience. **Appendix 4**. Family interview schedule.
11	**Anonymous SNOD Questionnaire Form B.**	SNODS complete a questionnaire after each approach conversation to document information on the family's understanding of their role, their attitudes and behaviours and the outcome of the process. Appendix 1. FORM B: Completed by Specialist Nurse in Organ donation**.**
12<br>12.1<br>12.2	**Focus Groups with SNODS.<br>Interviews with Specialist Requesters.<br>Interviews with**	Focus groups with key SNODS, managers and specialist requesters in the North West team and South Wales team to explore SNODS experiences of implementing the act in practice. Minimum 23 participants.

	**team and regional managers**	
13	**Field Notes from interviews**	Researchers and transcribers document their thoughts and views from interview.
14	**Interim feedback from Patient and Public representatives, (PPI's), SNOD's, Managers, NHSBT, NHS, Clinical Leads Organ donation, and other key stakeholders.**	A two day interim findings conference was held in Birmingham on the 9th and 10th November 2016. The purpose was to present interim findings to a key group of 50 NHSBT staff, NHS staff, Welsh Government representatives and PPIs. Feedback was collected on 10 presentations reviewing the various datasets thus far.
15	**Research team perspectives**	Weekly team meetings and monthly data analysis meetings are recorded to capture the ongoing analysis and interpretation of data and to put findings into wider context and highlight issues needing further attention.
		**Additional contextual data produced by the research team to situate the evaluation findings**
16	**Update of the literature**	Update of the systematic reviews in 8.
17	**Discourse Analysis of the press & media**	The discourse analysis will include the public media campaign, press articles and news stories promoting the changes.

For peer review only

FORM B Organ Donation Study: Questionnaire for SNOD to document outcome of all approach conversations

Record of conversation with (tick all that apply): spouse or partner ☐ **parent or child** ☐ **brother or sister** ☐ **grandparent or grandchild** ☐ **niece or nephew** ☐ **stepfather or stepmother** ☐ **half-brother or half-sister** ☐ **friend of long standing** ☐ **appointed representative** ☐ (referred to as 'family' hereafter)

Age range of deceased person: ☐ **0-18** ☐ **19-35** ☐ **36-50** ☐ **51-70** ☐ **>71**

What region in Wales did family members/friends come from (tick all that apply): ☐ Isle of Anglesey ☐ Conwy ☐ Gwynedd ☐ Denbighshire ☐ Flintshire ☐ Wrexham ☐ Powys ☐ Ceredigion ☐ Pembrokeshire ☐ Carmarthenshire ☐ Swansea ☐ Neath Port Talbot ☐ Bridgend ☐ Rhondda Cynon Taff ☐ Merthyr Tydfil ☐ Blaenau ☐ Torfaen ☐ Monmouthshire ☐ Caerphilly ☐ Newport ☐ Cardiff ☐ Vale of Glamorgan ☐ Outside of Wales

Clear Form

1. Was a SNOD involved in the donation conversation?
☐ Yes ☐ No

2. Did the patient die in hospital in:
☐ Wales (go to Q3b) ☐ England (go to Q3)

3. For Welsh patients that died in England what was the patient registered decision on ODR? ☐ Opt In ☐ Opt Out ☐ Appoint Representative ☐ Not Registered

Would the patient have fitted deemed consent criteria if they had died in Wales? ☐ Yes ☐ No

Did the family think that deemed consent would apply?
☐ Yes ☐ No ☐ Uncertain ☐ Non Applicable (N/A)

Was consent obtained ? ☐ Yes ☐ No ☐ N/A

Had the family heard or read about the Act from the media campaign (Radio, TV, newspapers, postal leaflet etc)?
☐ Yes ☐ No ☐ Uncertain ☐ N/A

Were the family positive about the new Act?
☐ Yes ☐ No ☐ Uncertain ☐ N/A (go to Q.19)

3b. For Welsh patients who died in Wales, what was their registered decision on the ODR? ☐ Opt In ☐ Opt Out
Appoint Representative ☐ Not Registered ☐

4. If patient was under 18 was their decision recorded on ODR?
☐ Yes ☐ No ☐ Non Applicable (N/A)

5. For patients of any age, did the family know of any other expressed decision?
☐ Yes ☐ No ☐ Uncertain ☐ N/A

6. Did the family agree with the patient's expressed donation decision? ☐ Yes ☐ No ☐ Uncertain ☐ N/A

7. Was there disagreement between one or more family members about the patient's expressed decision?
☐ Yes ☐ No ☐ Uncertain ☐ N/A

8. Was the patient's registered or expressed donation decision supported? ☐ Yes ☐ No ☐ N/A

9. Only for patients who appointed a representative (AR).

Did the AR give consent? ☐ *Yes* ☐ *No*

Did the AR support new Act? ☐ *Yes* ☐ *No* ☐ *Uncertain*

Were the family aware of AR? ☐ *Yes* ☐ *No* ☐ *Uncertain*

Did the family agree with the decision of the AR?

☐ *Yes* ☐ *No* ☐ *Uncertain* ☐ *N/A*

10. Did deemed consent apply? ☐ Yes ☐ No ☐ N/A

11. Did the family agree with the deemed consent of the patient?
☐ Yes ☐ No ☐ N/A
If no, did you feel able to get the family to support the deemed consent? ☐ Yes ☐ No ☐ N/A

12. Was there disagreement within the family and close friends involved in the deemed consent conversation?
☐ Yes ☐ No ☐ Uncertain ☐ N/A
If yes, did you feel able to get the group to support the deemed consent? ☐ Yes ☐ No ☐ N/A

13. Was consent obtained? ☐ Yes ☐ No ☐ N/A

14. Who consented? ☐ family member ☐ friend ☐ other ☐ N/A

15. Had the family heard or read about the Act from the media campaign (Radio, TV, newspapers, postal leaflet etc)?
☐ Yes ☐ No ☐ Uncertain ☐ N/A

16. Did the family have a correct understanding of the Act BEFORE you spoke to them? ☐ Yes ☐ No ☐ Uncertain ☐ N/A

17. Did the family have a correct understanding of the Act AFTER you spoke to them? ☐ Yes ☐ No ☐ Uncertain ☐ N/A

18. Had the new Act changed the family views of organ donation?
☐ Yes ☐ No ☐ Uncertain ☐ N/A
If yes, were they positive about the new Act? ☐ Yes ☐ No

19. Did any person suggest that they would register their decision in light of your conversation?
☐ Yes ☐ No ☐ N/A
If yes, would they: ☐ Opt in ☐ Opt out
☐ Appoint Representative ☐ Do nothing

20. Has this Act influenced your approach conversation with this family? ☐ Yes ☐ No ☐ Uncertain ☐ N/A

Anything else that could be helpful (e.g. Was there a pre-approach, a complaint or anything in this case that impacted upon you getting consent under the new law?)

Return by email to organdonationstudys@nhsbt.nhs.uk (for South Wales team) as soon as possible.

Submit

For peer review only - http://bmjopen.bmj.com/site/about/guidelines.xhtml

FORM C: Organ Donation: Questionnaire for Family/ Close Friends/ Appointed Representatives

Thank you for agreeing to fill out this questionnaire. Anybody involved in the donation decision can choose to fill out a questionnaire. The questions are quite short but feel free to add more details at the bottom. There is an option complete this online if you prefer: https://bangor.onlinesurveys.ac.uk/organ-donation-project-questionnaire. There is no limit to the number of questionnaires per family. By returning the completed questionnaire to the research team at Bangor University, it will be assumed that you have given your consent for the researchers to analyse and use the data.

1. Are you: spouse or partner ☐ parent or child ☐ brother or sister ☐ grandparent or grandchild ☐ niece or nephew ☐ stepfather or stepmother ☐ half-brother or half-sister ☐ friend of long standing ☐ Appointed Representative ☐

(*An appointed representative is a person appointed by your loved one or close friend during their lifetime to convey their organ donation decision after their death.)*

2. Did your loved one or close friend pass away in a hospital in:
Wales ☐ (go to Q.4) England ☐ (go to Q.3)

3. Did you know that the changes to consenting to organ donation in Wales did not apply in England? Yes ☐ No ☐ Uncertain ☐

4. On the organ donor register, did your loved one or close friend:
Opt In ☐ Opt Out ☐ Appoint a Representative ☐ Do Nothing ☐

5. Did you know about your loved one or close friend's decision on the organ donor register?
Yes ☐ No ☐ Uncertain ☐ Non Applicable ☐

6. Did your loved one or close friend ever discuss their donation decision with you?
Yes ☐ No ☐ Uncertain ☐ Non Applicable ☐

7. Did you support your loved one or close friend's organ donation decision after they passed away?
Yes ☐ No ☐ Uncertain ☐ Non Applicable ☐

8. Did you feel able to support the deemed consent of your loved one or close friend when the changes to consenting to organ donation in Wales were explained by a specialist nurse in organ donation?
Yes ☐ No ☐ Uncertain ☐ Non Applicable ☐

9. Do you consider yourself the decision maker on behalf of your loved one or friend who passed away? Yes ☐ No ☐ (go toQ.11)

10. As the decision maker, did the changes to consenting to organ donation in Wales help you at this difficult time?
Yes ☐ No ☐ Uncertain ☐

Please turn over the page now

For peer review only

11. Was there any form of disagreement between anybody involved in the donation discussion?
Yes ☐ No ☐ Uncertain ☐ Non Applicable ☐
If yes, did the changes to consenting to organ donation in Wales help to reach agreement?
Yes ☐ No ☐ Uncertain ☐ Non Applicable ☐

12. When somebody sadly passes away and they meet very specific criteria in Wales, if they have NOT registered a decision on the organ donor register or have NOT discussed their organ donation decision with family and friends, it means that their consent to organ donation can be deemed.
On a scale of 1 to 10 (1 = not at all and 10 = fully understood)

How well would you say you understood the changes to consenting to organ donation in Wales BEFORE you spoke to a specialist nurse in organ donation?
1 ☐ 2 ☐ 3 ☐ 4 ☐ 5 ☐ 6 ☐ 7 ☐ 8 ☐ 9 ☐ 10 ☐

How well would you say you understood the changes to consenting to organ donation in Wales AFTER you spoke to a specialist nurse in organ donation?
1 ☐ 2 ☐ 3 ☐ 4 ☐ 5 ☐ 6 ☐ 7 ☐ 8 ☐ 9 ☐ 10 ☐

13. Had you heard or read about the changes to consenting to organ donation in Wales from the media campaign (Radio, TV, newspapers, postal leaflet etc.)?
Yes ☐ No ☐ Uncertain ☐

14. Have the changes to consenting to organ donation encouraged you to register your decision on the organ donation register?
Yes ☐ No ☐ Uncertain ☐ Non Applicable ☐

Other comments Please tell us anything else that you think is important.

____
____
____
____
____
____
____
____
____
____
____
____
____

Thank you kindly for your responses at this difficult time. If you would like to share your stories with us in more detail, please fill out Form D (consent to be contacted for interview) and return with this questionnaire in the pre-paid envelope. We look forward to hearing from you.

For peer review only

Overview of the Interview protocol

The interview provides an opportunity for the 'family member' to talk about their decisions and experiences of organ donation and deemed consent (if applicable) at a time that suits them after their bereavement.

The new way of consenting to organ donation came into law in Wales on the 1st December 2015. Families who have experienced a bereavement after 1st of December are the most affected by this change. The interview with the family enables greater clarity of the very personal experience of the new way of consenting to organ donation. The stories the family provide and the meanings they attribute to their beliefs, decisions and actions will support overall understanding of the ways the new Act impacts upon organ donation and transplantation in Wales.

Context for interviews

The interview will be semi-structured and available to families in Welsh or English through any mechanism the family chooses for example face to face, telephone, skype or social media.

The topic guide (see below) provides a loose structure to anchor the interview on the implementation of the Act. Participants will be free to talk in detail about their views, experiences and personal meaning in greater depth. This will elicit what the Act and its implications (such as registration on the organ donor register) and their behaviours and actions meant for them.

The interview guide will need adapting for each unique situation and the questioning will change depending on if the interviewee and others involved in the conversation supported the donation decision of their loved one or close friend or not.

Data arising from the interview will support and enrich the data analyzed in the SNOD & family questionnaires. Outcomes from the interview will help focus the study as it begins to reach conclusions from early analysis of data, and steer the remaining phases and support key findings.

English and Welsh will be analyzed separately and then brought back into the overall analysis.

Interview arrangement process

1. An agreed convenient date, time and venue will be agreed by the family's preference.

2. The family will choose what format the interview will take for example face to face, telephone, skype.

3. The researchers will follow the distress protocol (in your pack) when interviewing.

4. Check all kit is working prior to interview

Taking consent

5. The researcher will provide an overview of the study to the family using the 'overview' above as a guide and referring to the study information booklet.

6. The researcher will explain the recording equipment briefly and how it will be used.

7. The researcher will explain how the interview will proceed.

For peer review only

8. The participants will be given opportunity to ask questions.

9. The researcher will ask family to fill out two copies of the consent to interview form (in your pack).

10. The researcher will provide participants with a copy of the signed consent form to keep.

11. After the interview, if considered appropriate, the researcher will share a pamphlet of CRUSE Bereavement Care Literature and explain who they are and how they can help.

Beginning the interview

12. Following the Distress Protocol - continue to observe for signs of stress before, during and after the interview

13. Discuss options of pausing or stopping the interview and remind family of why you are here for example 'I am here to listen to your vitally important stories. My input will be minimal i.e. 'less talking and more listening'. The experience should be informal and relatively relaxed. If it becomes anything else than this then it is time to pause and you must let me know.'

14. Start recording

15. Begin by stating date / time / location / and people present

*For Phone or other remote interviews consent will be obtained through the post ideally before the interview or as soon as possible after the interview. We cannot use the data given until written consent is obtained.

Introductory conversation:

Researcher should offer their condolences. Ask a bit more about them and ask a bit more about the person that died.

Say up front that there are 4 specific areas that you would like to cover in the interview:
1. General views about organ donation.
2. The new way of consenting to organ donation in Wales.
3. The media campaign promoting the new way of consenting to organ donation.
4. Their personal experiences of the organ donation process.

For peer review only

1. Topic area 1, general views about organ donation. (contextual questions)

1.1 - Have you had any previous experiences of organ donation before your loved one or close friend recently passed away?
1.2 - Do you have a personal view about organ donation?
1.3 - Has your personal view changed over time?
1.4 - Are you registered on the ODR? If so – what decision is registered?
1.5 - How do you generally feel about Organ Donation now after your loved one or close friend recently passed away?

Prompts:
Tell me more about..?
Can we go back to and talk further about?

2. Topic area 2, the new way of consenting to organ donation in Wales. (core questions)

'Changes to consenting in Wales came in on 1st December 2015. The law has changed to make it easier for people to donate their organs.'

2.1 - Are you normally resident in Wales or England?
2.2 - Had you heard about the new way of consenting to organ donation before your friend or loved one passed away?
2.3 - Can you explain in your own words what are the new changes to consenting to organ donation in Wales?

Probe and prompt as appropriate.

Researcher then follow with gently providing a clear context to facilitate clarification and further discussion:

'The new Law gives the decision about organ donation to the person to make during their lifetime. During their lifetime, people normally resident in Wales are asked to opt out or opt in to donation by registering a decision on the organ donation register or by discussing it with family and friends, or they can do nothing. If they do nothing and they meet specific criteria - it is assumed that they have no objection to being an organ donor. Citizens of Wales are actively encouraged to discuss their organ donation decisions with their friends and families. They can also appoint a representative to convey their decision on their behalf.'

2.4 - Is registering a decision on the organ donor register important to you, why?
2.5 - Did you know that you can register your decision through a conversation with a family member, not just by registering on the ODR?
2.6 - What does 'doing nothing' mean to you?
2.7 - Did you realise that 'doing nothing' is actually a choice? That is if you 'do nothing' then you support organ donation?
2.8 - What does deemed consent mean to you?
2.9- How do you feel about these changes to consent to organ donation now?
2.10 (If the deceased person was a child – did the new Act impact on their views and decision-making? If so how?)
2.11 (If the deceased person was normally resident in Wales and died in England - did the new Act impact on their views and decision-making? If so, how?)
2.12 If appropriate (for example if family are very positive about donation, proactively support donation are enthusiastic to express their views on the changes etc…) explore further families understanding of decision making especially deemed consent through 'what if' scenario. E.g. If you didn't know

the decision of your loved one would you have accepted deemed consent? Having been through the donation process would it be easier or harder to go through the donation process if you didn't know your loved ones wishes and consent was deemed, why? In your opinion does deemed consent have an equal status with a registered or a verbal opt in decision, why? In your opinion what needs to be improved in the changes to legislation?

Prompts
Tell me more about that…?
What was your understanding of…?
What does that mean for you…?
How do you feel about….?
You mentioned…could you tell me a bit more about that?

3. Topic area 3, organ donation media campaigns in Wales (core questions)

'Thank you, can we move on and focus on hearing more about what you saw in the media about the changes to consenting to organ donation in Wales prior to 1st December.'

3.1 - Had you seen any of the organ donation advertising campaign (television advert, billboards, bus campaign, every home in Wales got several letters, emails etc.)?
    3.1(a) - If so which advertising material had you seen?
3.2 - Can you remember the key messages from the advertising material?
3.3 - What did you think about the messages?
3.4 - Did you understand the key messages?
3.5 - What did you do as a result of seeing the advertising campaign?'

Prompt
Focus specifically on their actions to the media: opt in, opt out on ODR, do nothing with assumption of agreement to organ donation, discuss donation and donation decision with family, appoint a representative.

Following this discussion about what they remembered the researcher should now gently show the interviewee some of the organ donation changes advertising materials (from interviewer pack). These props may help to further frame this question and further jog memory. Note which one interviewer showing to assist transcription).

Focus again specifically on their actions as a result - Opt in, opt out, do nothing, discuss with family, appoint a representative.

Prompts:
Do you remember seeing any of these?
What did you think when you saw it first?
What would have made that difference to make you notice it more?
What do the messages mean to you now?
Can we go back to and talk further about…?

4. Topic guide 4, their detailed experience of the organ donation process.

Specific questions for family members and close friends involved in the organ donation approach conversation with professionals.

4.1 Establish which elements of consent applied in this case:

Prompts

Had your loved one or close friend registered an organ donation decision on the ODR (ask if opt in or opt out?)

OR Did your loved one or close friend 'do nothing'?

Had your loved one or close friend expressed an organ donation decision during their lifetime? If so what did they say and to whom?

Did Deemed consent apply? (explain deemed consent again if appropiate)

(Be specific as to what deceased persons donation decision was - Opt in opt out, expressed decision during lifetime, do nothing and assume consent, appoint AR)

4.2 - Do you know if (deceased person's) view changed over time?

4.2(a) If yes, explore fully when and why.

4.3 - What was the organ donation decision? Consent or no consent?

4.3(a) If decision different to deceased persons decision, how did you go about overturning the decision?

4.4- How well prepared did you feel for how very ill your relative really was?

4.5 - Did anyone talk to you about palliative or end of life care for your relative or close friend?

4.6 - When did you realise that they may not survive?

4.7 - In terms of NHS processes what helped at this time?

4.8 - Is there anything else that might have helped you?

Prompts

How did that make you feel?

Can you tell me more about those moments?

You mentioned…can you tell me more about it?

Can we go back to and talk further about…?

4.9 - Can you remember your first thoughts when you were approached about organ donation when your loved one was very ill?

4.10 - Who first approached you about organ donation (doctor, nurse, SNOD)?

4.11 - How did the organ donation conversation play out in your mind?

4.12 - Who was involved in the donation discussion (e.g. Family members, friends, others)

Prompts, focus on role of SNOD, roles of local clinicians. Unpack how many meetings/conversations were had and with whom.

4.13 - Did you feel that you could express your views about your loved ones organ donation decision during the conversation?

4.14 - Did anyone or anything influence your view on organ donation and your relative/close friend?

4.15 - Other than the professionals - who did you talk to in detail about your relative/close friend and organ donation?

Prompts

Unpack other family/friend & other positive and negative influences

4.16 - Was there agreement amongst those involved?

4.16(a) - If there was disagreement – what was the disagreement about?

4.16(b) - Was the disagreement resolved and how?

4.17- In the end, did everyone support the donation decision of your loved one or close friend?

4.17(a) - If not, whose opinion counted most?

4.18 - Was it easier just to say no/yes?

4.19 - Who conveyed the decision to the professionals?

For peer review only

4.20 - Is there anything else you think that the professionals could have helped you with in this situation?

Prompts
How did you feel about it?
Can you tell me more about…?
Can we go back to where you mentioned and talk further about…? *(Unpack if there were any specific cultural influences that impacted on the role and influence of the interviewee on the decision-making process)*

4.21 **- If deceased persons decision was changed -**
– 'For you, was the primary consideration more about making things easier for you and the family looking forward, rather than honouring the donation decision of your loved one or close friend?'
4.22(a) - Do you think there are any negative consequences and impacts of not supporting the deceased person's decision.
4.22(b) - Do you think there are any positive consequences and impacts of not supporting the deceased person's decision.
(unpack if interviewee would act in the same way now as they did then)

Signal disengagement and end of interview coming up.

4.22 - Is there anything else you would like to share that hasn't been covered?
4.23 – Finally, would you accept a transplant if you needed one?

'This has been very valuable'
'Your insights and stories have really helped'

Leave time for an open discussion.

Bring discussion back to a positive place to conclude the interview.

Thank you very much. We have come to the end of the interview.

Check that you have covered/know the following details

Record of conversation with:
spouse or partner parent or child brother or sister grandparent or grandchild niece or nephew stepfather or stepmother half-brother or half-sister friend of long standing
Age Range of Interviewee 0-18 19-35 36-50 51-70 >71
Age Range of Deceased Person 0-18 19-35 36-50 51-70 >71
Number of people present at interview
Time passed since death
Was death expected
Area where interviewee resides
Area where person died
Area where deceased person resided
Was deceased person BME

Post Interview Arrangements

16. Researcher should thank the interviewee for their time

17. Give the interviewee contact details (on the back of the information booklet) should they wish to follow anything up.

For peer review only

18. Ask if they can follow up the interview with a courtesy phone call in the near future

19. Reintroduce the support literature while preparing to leave.

Uploading and storing data

20. Researcher should upload data to ‘U: Drive’ ASAP after the interview
21. Ensure data is properly uploaded after the interview before deleting from recording device.
22. Begin transcribing ASAP after the interview.
23. Assign a study code at point of transcription.

Follow up protocol

24. Researcher should follow up with a courtesy call after 6 weeks from date of interview (if permission given).
25. Researcher should assess if family member feels satisfied that they have communicated everything they wanted to.
26 If family member still wants to give their views to researcher, researcher should explore other options such as a quick conversation there and then on the phone, or a follow up email correspondence. Researcher should also if appropriate direct family member to CRUSE bereavement care for long term professional support.

* Researchers should follow protocols where relevant set out in Safety Code of Practice, Lone Working Policy & Field Work Safety Documents available from Bangor University and the project folder on University U Drive.

For peer review only

For peer review only

Appendix 3. Ethical issues and strategies

Undertaking research with vulnerable participants: Grieving families, close friends and appointed representatives are vulnerable and considered to be most affected by the Act. It appears appropriate, with the right support and safeguards in place, to provide an opportunity for them to participate in a study to elicit their perspectives. Our patient representatives have written a letter of support indicating that families will want the option to be able to talk to someone outside of the NHSBT Team, to share their positive or negative views about how their role has changed under the new Act and whether they agreed with the decision of the deceased person or not.

Although they have been recently bereaved, potential donor families, close friends and appointed representatives may want an opportunity and may benefit from expressing their views, which in turn may inform development of practice in a continuous improvement cycle. To ensure that an appropriate approach is made to potential participants at an appropriate time, we will ask the Specialist Nurses in Organ Donation (SNODs) to use their judgement as to the most appropriate time to share information on the study. SNODs will have spent a lot of time with the people involved in the donation conversation and can use their professional judgement to select from a range of methods to recruit participants that are individually tailored for each situation, including: via direct contact with families by SNODs; by sending study invitation and information attached to routine follow up communication by SNODs; by direct mailing of study invitation and information by NHS Blood and Transplant. In addition we will place three adverts in the national media at staged intervals and recruit through snowball sampling. If a family member, close friend or appointed representative knows of another person involved in the organ donation process who may want to be interviewed, we will ask them to share a letter of invitation and study information with them and ask the person to contact us directly.

We will follow the sensitive ethical framework[2] and practical strategies shown in Table 1. Participants who return self complete anonymised questionnaires and who wish to themselves remain anonymous will be made aware that returning a completed questionnaire to the research team will constitute consent to use the data. For all other research procedures such as interviews informed written consent will be required. All participants will be over 16 years with mental capacity to consent. Participants can choose to be interviewed at any point that is appropriate and convenient for them after their bereavement up until the end point of data collection. If an individual receives more than one letter of invitation we will include a sentence to explain that, if they have already made their decision whether to participate in the study or not, to ignore the letter or to pass the invitation on to another family member or close friend of the deceased person, because NHSBT only have one contact name for each case. Researchers will follow a 'Distress Protocol' (see below) when conducting interviews and if participants become unduly distressed they will use the three step approach in the protocol to safeguard the wellbeing of participants.

CRUSE Bereavement Care Cymru aim to reach out and support all bereaved people in Wales. We will share their client information with participants and the contact numbers for CRUSE bereavement care and support. If the research officer has any serious concerns

For peer review only

about the safety of any participant in the study, they will follow the standard protocol of NHSBT for Safeguarding Vulnerable People.

Anonymity of Professionals: Interviews with professionals will be conducted at a venue of their choice, and they will be assigned a code. Where there is only one role in the organisation care will be taken not to identify participants by using their quotes without permission. Participants will be offered a choice of 1:1 or small group or focus group interviews if they do not wish their experiences to be shared with colleagues.

Support for researchers undertaking sensitive interviews: The research team is large (5 core members) to provide a mutually supportive context and debriefing will be offered after interviews. Researchers have been recruited with skills and experience of undertaking interviews on sensitive topics with vulnerable people. The research team is also working in partnership with NHSBT teams who provide an additional supportive environment and peer to peer support and mentorship concerning sensitive issues, should research team members need it. We have built in three joint professional development opportunities for support, shared learning and reflection. Researchers can also contact CRUSE bereavement care for additional confidential and independent support.

Good clinical practice. Core research team members have undertaken Good Clinical Practice in research training and those who will have contact with family members have been subject to screening under the Disclosure and Barring Service.

Data Protection and Data Sharing Agreement

The research requires anonymised data to be collected and shared between NHSBT and Bangor University. Bangor University has entered into a standard data sharing agreement with NHSBT. Data collected directly by the research team will be recorded on encrypted digital recorders, regularly downloaded, assigned a unique code and transcribed, anonymised and stored securely on password protected university servers and laptops. Data on digital recorders will then be deleted. Any paper based patient identifiable information will be stored under lock in a secure place with access controlled by the research team.

The following process has been previously adopted in other studies to share non-patient identifiable information. The process is designed to enable the research team at Bangor University to have access to anonymised information recorded by SNODs that sheds light on decision-making by families/appointed representative(s) (especially when they say no to organ donation). Patient and Public representatives from partner non-government organisations who support bereaved families have been consulted and support this approach. The structured communication process used by SNODs during the donation request stage will be amended for introduction of the new Act and documented in NHSBT Standard Operating Procedures and Management Process Descriptions. The 'approach' conversation is highly structured and follows a ladder of issues for families to think about, consider and discuss. The SNOD is trained to explore and clarify any issues or questions that are raised and to facilitate and listen to reasons and concerns when considering giving or declining consent to donation. To capture this information in a structured and anonymised way that falls outside of the Data Protection Act, we have agreed a plan with the two NHSBT donor teams covering Wales, to jointly develop a standard proforma for SNODs to record

For peer review only

key anonymised information from the ‘approach’ conversation for research purposes as soon as practical after it has concluded and once they have disengaged from the family. The anonymised information recorded on the proforma will be returned electronically to the researchers in batches so that it is not possible to link any single proforma with any specific death. We will also be respectful of professional anonymity and the proformas will be designed in such a way that the identity of the hospital or person who completed it will not be known to researchers.

Table 1. Study framework for ethical decision-making.[2]

Ethical considerations	Practical strategies
	**Participant identification and recruitment**
Access, confidentiality Regard	Formally obtain the support of a key person to undertake the role of identifying potential participants and disseminating pre-prepared recruitment packs on behalf of the research team.<br>Recruit potential participants in a serial manner, for example, send out a maximum of five recruitment packs at any one time so that participants are not kept waiting for long periods before the research interview.
Respect, relevance	Consider participant inclusion criteria of bereaved no less than 3 months and no more than 12 months at the time of recruitment to the study. * *We will ask Specialist Nurses in Organ Donation to share study information at the time of bereavement and will offer the option of a self-complete questionnaire that involves no face to face contact with researchers and an interview at a time when the participant feels that it is appropriate for them. We have built in a 3 month time lag to collect data after the last participant has been contacted.*
Compassion	Include a covering letter that introduces the study in a personalised way by taking familiarity into consideration.
Informed choice	Provide clear written and web-based information about the researchers and the study.<br>Include an invitation to contact the researcher.<br>Demonstrate timely responsiveness to any potential questions or queries.
Non-coercion	Provide a minimum of 10 days for participants to decide about joining the study.
	**The research interview**
Choice, respect	Agree a convenient date, time and venue for the research interview. Avoid dates that coincide with any significant family events or anniversaries.
Safety	Implement a study site policy for researchers working alone in advance of the interview encounter.
Safety, support	Competent researcher with experience of conducting sensitive research interviews and supporting the bereaved.
Choice, privacy	Provide the option of an interview face to face or remotely, for example, via telephone.
Informed consent	Provide an overview of the study and present opportunity for participants to ask questions.<br>Explain how the interview will proceed. Obtain written agreement to audio-record the interview and to use anonymous quotes in any presentation of the research. Provide participants with a copy of the signed consent form to keep.
Support	Discuss and agree avenues of post-interview support prior to the interview commencing<br>Observe/listen for signs of distress during the interview.<br>Discuss the option of pausing the<br>recording or stopping the interview. Plan a natural break for refreshments.
Confidentiality, anonymity	Ensure audio-recordings and transcripts are securely stored and electronic data are password<br>protected. Assign a study code at the point of transcription.
	**Post-interview follow-up care**

For peer review only

Support	Arrange a convenient time to telephone the participant (normally in 24–48 h) to check on any issues the interview may have raised and to answer any questions.<br>Compile information about local support organisations. Offer this to participants if they consider it helpful and/or direct them to appropriate professionals to discuss any issues of concern.<br>Establish if participants wish their general practitioner (GP) to be informed about their participation in the study and obtain written consent to proceed. Provide GP with information about the study at the time of notification.
Appreciation	Send participants a personal thank-you letter and offer an executive summary of the research findings.
Involvement	Provide participants with an opportunity to evaluate their experience of participating in bereavement research.
Researcher Support	Determine support for the researcher from an individual with whom they feel comfortable and who is suitably qualified to provide support. Plan a debriefing session after each interview encounter. Utilise reflexive notes to guide the discussion.

Distress Protocol

During instances of bereaved participants becoming distressed during the interview process, the subsequent protocol will be followed.

Identifying Distress

The interviewer will be mindful of signs of distress in the participants throughout the interviews. Signs of distress to look out for will include:

- Exhibition of behaviours that indicate that the discussion has become too upsetting for them, including crying and an inability to continue for example.
- The participant verbally communicating that they are experiencing distress during the interview.

Response Stage 1

- The interview will be stopped.
- The participant will be offered a break, have a drink of water/ tea etc.
- The participant will then be asked if they would like to continue the interview or if they would prefer to discontinue. Should they wish to go on, the interview will resume.

Response Stage 2

If the participant elects to discontinue the interview,

- The interview will not continue.
- The interviewer will signpost where support can be obtained such as from the SNOD or the bereavement support service at the hospital where their relative/friend died, or from their GP.
- The participants will also be reminded again of the contact details for Cruse Bereavement Care,' an organisation which provides support for bereaved people.

Response Stage 3

- At a later date, the interviewer will follow up with the participant with a courtesy call (with the participant's consent). If the participant feels strongly that they would still like to have their views and experiences heard – the interviewer will go through options (wait a while before rearranging, explore other methods rather than face to face etc.

References

1. The Data Protection Act 1998. Reviewed March 2017 http://www.legislation.gov.uk/ukpga/1998/29/contents (Accessed March 2017).
2. Sque M. Walker W. and Long-Sutehall T. (2014) Research with bereaved families: A framework for ethical decision-making. Nursing Ethics doi:10.1177/0969733014521097.

For peer review only
